# Parasitic Light Absorption, Rate Laws and Heterojunctions in the Photocatalytic Oxidation of Arsenic(III) Using Composite TiO_2_/Fe_2_O_3_

**DOI:** 10.1002/chem.202104181

**Published:** 2022-02-24

**Authors:** Jay C. Bullen, Hany F. Heiba, Andreas Kafizas, Dominik J. Weiss

**Affiliations:** ^1^ Department of Earth Science and Engineering Faculty of Engineering Imperial College London London SW7 2BX UK; ^2^ Department of Chemistry White City Campus Imperial College London London W12 OBZ UK; ^3^ London Centre for Nanotechnology London SW7 2AZ UK; ^4^ Marine Chemistry Department Environmental Division National Institute of Oceanography and Fisheries NIOF) Egypt; ^5^ The Grantham Institute Faculty of Natural Sciences Imperial College London London SW7 2AZ UK; ^6^ Civil and Environmental Engineering, E-Quad Princeton University Princeton USA

**Keywords:** arsenic, composite, photocatalysis, photocatalytic oxidation, water treatment

## Abstract

Composite photocatalyst‐adsorbents such as TiO_2_/Fe_2_O_3_ are promising materials for the one‐step treatment of arsenite contaminated water. However, no previous study has investigated how coupling TiO_2_ with Fe_2_O_3_ influences the photocatalytic oxidation of arsenic(III). Herein, we develop new hybrid experiment/modelling approaches to study light absorption, charge carrier behaviour and changes in the rate law of the TiO_2_/Fe_2_O_3_ system, using UV‐Vis spectroscopy, transient absorption spectroscopy (TAS), and kinetic analysis. Whilst coupling TiO_2_ with Fe_2_O_3_ improves total arsenic removal by adsorption, oxidation rates significantly decrease (up to a factor of 60), primarily due to the parasitic absorption of light by Fe_2_O_3_ (88 % of photons at 368 nm) and secondly due to changes in the rate law from disguised zero‐order kinetics to first‐order kinetics. Charge transfer across this TiO_2_‐Fe_2_O_3_ heterojunction is not observed. Our study demonstrates the first application of a multi‐adsorbate surface complexation model (SCM) towards describing As(III) oxidation kinetics which, unlike Langmuir‐Hinshelwood kinetics, includes the competitive adsorption of As(V). We further highlight the importance of parasitic light absorption and catalyst fouling when designing heterogeneous photocatalysts for As(III) remediation.

## Graphical abstract

**The mechanisms** that control the kinetics of arsenic(III) photocatalytic oxidation when meso‐TiO_2_ is coupled with an Fe_2_O_3_ adsorbent phase are investigated herein. Parasitic light absorption is the primary factor, followed by changes in rate law (from disguised zero‐order to first order). Charge transfer across the heterojunction in our material is negligible.

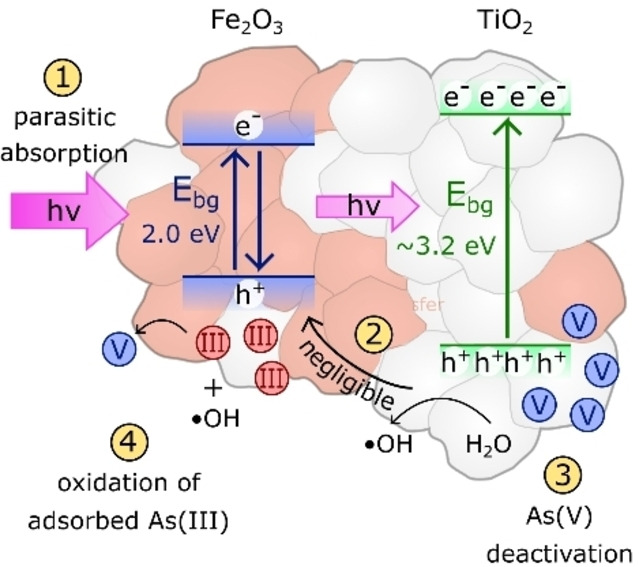

## Introduction

Arsenic is a carcinogen responsible for an estimated 30–40,000 deaths per year in Bangladesh alone,^[1]^ with tens of millions more people at risk.^[2]^ In the anoxic and reducing groundwaters of Bangladesh and West Bengal in India, arsenic is present as inorganic As(III) (*arsenite*), with the formula H_3_AsO_3_. Certain studies suggest that As(III) is up to 60 times more toxic than As(V)^[3]^ and, due to its neutral charge, As(III) is more difficult to remove than As(V) (*arsenate*, which is found as HAsO_4_^2‐^ and H_2_AsO_4_^−^ in oxic groundwaters).^[4]^ Adsorption onto iron oxides (with positive surface charge at neutral pH) is the preferred method for the removal of As(V).^[5]^ However, the removal of As(III) via adsorption is limited due to the competitive adsorption of oxyanions such as phosphate (PO_4_^3−^) and sulfate (SO_4_^2−^).^[6]^ This limits the lifetimes of arsenic filter devices, resulting in the failure of many arsenic mitigation schemes: replacing the sorbent media when saturated can be prohibitively expensive (exacerbated by the lack of market availability in rural communities) and users lack the confidence and know‐how to regenerate the saturated sorbent media independently.[^7^, ^8^] Oxidation of As(III) to As(V) is therefore needed to increase adsorption capacity, improving filter device lifetimes for the more effective remediation of As(III).^[9]^

The application of single‐phase heterogeneous photocatalysts such as TiO_2_ for the oxidation of As(III) has been explored over the past two decades.^[10]^ Unlike chemical oxidants such as chlorine, ozone and Fenton's reagent, heterogeneous photocatalysts are not associated with the risk of toxic disinfection by‐products (DBPs) such as trihalomethanes (THM).[^11^, ^12^] (In fact, heterogeneous photocatalysts may play a role in the degradation of DBPs).^[13]^ Regardless of the approach towards oxidation, a second step is required to remove the As(V) that is produced, for example adsorption or coprecipitation.

Coupling photocatalysts with an adsorbent material possessing affinity for the adsorption of As(V) is a promising strategy for the one‐step remediation of As(III). This approach simplifies the oxidation‐adsorption treatment, by using a single material in a single reactor, which may be advantageous given that many arsenic treatment plants fail due to maintenance issues.[^7^, ^8^, ^14^, ^15^] A variety of different composite photocatalyst‐sorbent materials have been investigated.[^16^, ^17^, ^18^] Titania (TiO_2_) coupled with an iron oxide phase is the most popularly studied combination, since TiO_2_ and iron oxides are benchmark materials for the photocatalytic oxidation of As(III) and adsorption of As(V) respectively. Examples include TiO_2_ and iron oxide on slag iron;^[19]^ Fe_2_O_3_ on commercial TiO_2_ powders;^[20]^ TiO_2_/γ‐Fe_2_O_3_ nanoparticles;^[21]^ TiO_2_/Fe_2_O_3_ porous ceramic beads;^[22]^ TiO_2_/Fe_3_O_4_ nanoparticles;^[23]^ and TiO_2_/Fe_3_O_4_ nanosheets.^[24]^ Recent tertiary composites incorporating a conducting organic polymer with the aim of improving charge carrier separation include γ‐Fe_2_O_3_/polyaniline/TiO_2_ ^[25]^ Fe_3_O_4_/polyparaphenylene diamine/TiO_2_ ^[26]^ and TiO_2_/polythiophene/γ‐Fe_2_O_3_.^[27]^

Titania‐iron oxide composites are stable and reusable: previous studies have used NaOH to regenerate TiO_2_/Fe_2_O_3_ composites after the UV‐assisted remediation of As(III), with efficiency decreasing by only 7.5 % after 9 cycles,^[28]^ 12.5 % after 5 cycles,^[21]^ and 20 % after 5 cycles.^[22]^ Elsewhere, dissolution studies have shown that in the dark <8 μg L^−1^ TiO_2_ is dissolved after one month of immersion at pH 3 (40 m^2^ L^−1^ TiO_2_)^[29]^ and <0.24 mg L^−1^ Fe_2_O_3_ is dissolved after 20 h of immersion at pH 2.4 (5 g L^−1^ Fe_2_O_3_).^[30]^ The reductive dissolution of iron oxides is promoted under ultraviolet illumination, but only at acidic pH (e. g. pH 3) and in the presence of Fe(II) complexing ligands;[^31^, ^32^] conditions neither present in our experimental work nor in naturally occurring As(III)‐contaminated groundwaters.[^33^, ^34^, ^35^]

Despite significant research activity into these materials, no previous study has investigated how the photocatalytic oxidation of As(III) is affected when the TiO_2_ photocatalyst is coupled to an iron oxide sorbent phase (e. g. Fe_2_O_3_). For instance, no study has considered how the rate equation might change and the reasons why. Although iron oxides are established adsorbents for arsenic remediation,^[36]^ as single‐phase materials they are typically poor photocatalysts due to fast electron‐hole recombination kinetics^[37]^ and show negligible activity towards the oxidation of As(III).^[18]^ In composite photocatalysts, incorporation of an iron oxide phase may improve photocatalytic activity beyond the sum of its parts due to charge carrier transfer across the semiconductor heterojunction (which improves charge carrier lifetimes by reducing electron‐hole recombination rates).[^38^, ^39^] Alternatively, iron oxide phases may reduce reaction rates due to the parasitic absorption of incident photons, suppressing the photoexcitation of TiO_2_.^[40]^ There is also evidence that sorbent‐coupling prevents photocatalyst deactivation (catalyst fouling).^[41]^ Given the potential for iron oxides to both enhance and suppress TiO_2_ photocatalytic oxidation rates, it is essential to characterize the mechanisms by which material coupling influences the photocatalytic oxidation of As(III), to engineer effective treatments using these new composite photocatalyst‐sorbent materials. To date, no previous study has investigated the influence of parasitic absorption, charge transfer across the heterojunction, or changes to the rate law in the photocatalytic oxidation of As(III) using titania‐iron oxide composite photocatalysts.[^28^, ^42^, ^43^, ^44^]

We previously developed new adsorption models for TiO_2_/Fe_2_O_3_ composites, showing how the monolayer adsorption of As(V) onto TiO_2_/Fe_2_O_3_ obeys component additivity, i. e. the reaction proceeds as adsorption onto non‐interacting TiO_2_ and Fe_2_O_3_ surface components, unaffected when TiO_2_ and Fe_2_O_3_ are coupled into a composite structure.^[45]^ In contrast, As(III) adsorption is not component additive when there are differences between the morphology of (i) the TiO_2_/Fe_2_O_3_ composite and (ii) the single‐component TiO_2_ and Fe_2_O_3_ reference samples, since surface morphology influences the extent of multilayer As(III) adsorption. We also developed a kinetic adsorption model to explore how a treatment based upon simultaneous photocatalytic oxidation‐adsorption using TiO_2_/Fe_2_O_3_ would be best designed, finding that high concentrations of the media (compared with photocatalysis‐only reactors) are required to achieve sufficient sorbent lifetimes.^[46]^

Having previously established the adsorption mechanisms, the aim of the present study was to understand the photocatalytic oxidation of As(III) using TiO_2_/Fe_2_O_3_ composites, determining for the first time how coupling TiO_2_ with an Fe_2_O_3_ sorbent phase affects the photocatalytic oxidation of As(III), considering both the reaction kinetics and mechanisms. To this end, new hybrid experimental‐modelling approaches were developed.

We first synthesized and characterized TiO_2_ and TiO_2_/Fe_2_O_3_ photocatalysts, using the synthesis procedure described by Zhou et al.^[28]^ without further optimization, as an example of the typical TiO_2_/Fe_2_O_3_ composites prepared by precipitation to produce discrete TiO_2_ and Fe_2_O_3_ phases.[^20^, ^22^, ^38^, ^42^, ^45^, ^46^] Previous studies of TiO_2_/Fe_2_O_3_ composites have only considered arsenic removal at high As concentrations and/or in blank media.[^19^, ^20^, ^21^, ^28^, ^45^] We thus verify that coupling TiO_2_ with the Fe_2_O_3_ sorbent phase truly improves total arsenic removal under environmentally relevant conditions (the groundwaters in South Asia rarely exceed 1 mg L^−1^ arsenic,^[47]^ i. e. lower concentrations than those under which composite photocatalysts have been previously tested,[^24^, ^28^] and these waters are typically rich in competitor ions such as phosphate).^[33]^

Secondly, we studied the key mechanisms by which coupling TiO_2_ with Fe_2_O_3_ might influence the photocatalytic oxidation of As(III), i. e. parasitic absorption of ultraviolet light by Fe_2_O_3_, enhanced charge carrier separation across the TiO_2_−Fe_2_O_3_ heterojunction, and photocatalyst deactivation. We determined the extent of parasitic absorption using UV‐Vis spectroscopy and component additivity (the linear combination of experimental data collected using single‐phase TiO_2_ and Fe_2_O_3_ reference samples^[45]^). We then developed a new model to predict transient absorption spectroscopy (TAS) decay kinetics using component additivity and evaluate the extent of charge transfer across the heterojunction. We thus establish, for the first time, a mechanistic explanation for the differences in As(III) oxidation kinetics observed after mesoporous TiO_2_ is coupled with Fe_2_O_3_.

Thirdly, we determined the kinetic rate laws governing As(III) oxidation using meso‐TiO_2_ and meso‐TiO_2_/Fe_2_O_3_ photocatalysts, developing new models to reconcile the results of initial rates analysis with data at later times. Previous studies have been limited to modelling the photocatalytic oxidation of As(III) using zero‐order,^[48]^ pseudo‐first order (PFO)[^49^, ^50^] and Langmuir‐Hinshelwood (LH) kinetics.[^51^, ^52^] Due to the capability of adsorbed As(V) to deactivate TiO_2_ ^[41]^ we developed new kinetic models constrained using the multi‐surface, multi‐sorbate surface complexation model (SCM) reported in our previous work,^[45]^ incorporating the competitive adsorption of As(V) and As(III) into a model of oxidation kinetics for the first time. We performed these kinetic experiments at pH 7.3±0.1, representing As(III) contaminated waters in South Asia.[^33^, ^34^, ^35^]

## Results and Discussion

### Materials characterisation

The synthesised meso‐TiO_2_ was off‐white in colour. Fe_2_O_3_ and the meso‐TiO_2_/Fe_2_O_3_ composite were both red, with Fe_2_O_3_ being darker in color than the composite.

XRD patterns are provided in Supporting Information Figure S3, revealing the presence of both anatase (*I*4_1_/ *amd*) and rutile (*P*4_2_/ *mnm*) crystal phases of TiO_2_. Application of the Spurr and Myers equation^[53]^ indicates that anatase was the dominant phase of TiO_2_ (∼84 % anatase and ∼16 % rutile by mass in meso‐TiO_2_, and ∼86 % anatase, ∼14 % rutile in meso‐TiO_2_/Fe_2_O_3_). Hematite (α‐Fe_2_O_3_, *R*‐3*cH*) was the only crystalline iron oxide phase identified. Application of the Scherrer equation to the XRD pattern of meso‐TiO_2_ gave average crystallite diameters of 10.0±0.4 nm for the anatase component and 16.8±2.5 nm for the rutile component.

For meso‐TiO_2_/Fe_2_O_3_, average crystallite diameters of 13±2.1 nm were observed for the anatase component, 16±3.5 nm for the rutile component, and 18.5±3.1 nm for the Fe_2_O_3_ component. This agrees with transmission electron microscopy (TEM) analysis (Supporting Information Figure S4). Crystallites were largest in the Fe_2_O_3_ reference sample, with an average diameter of 20.5±5 nm. Scanning electron microscopy (SEM) indicated that the crystallites of meso‐TiO_2_ and meso‐TiO_2_/Fe_2_O_3_ were aggregated into larger particles between ∼0.5 and 50 μm in size (Supporting Information Figure S5). In contrast, the Fe_2_O_3_ reference sample was coarser, with many particles being up to 100 μm in size.

Using meso‐TiO_2_ and Fe_2_O_3_ as end‐member reference samples, XRF analysis indicated that meso‐TiO_2_/Fe_2_O_3_ was 44.4±2.4 % meso‐TiO_2_ and 55.6±3.0 % Fe_2_O_3_ by mass (section Supporting Information 2.4). This is close to the theoretical 50 : 50 mass ratio based upon the quantity of reagents used during synthesis. The BET‐specific surface areas of meso‐TiO_2_, meso‐TiO_2_/Fe_2_O_3_ and Fe_2_O_3_ were 146, 116, and 103 m^2^ g^−1^ respectively, and the average pore sizes were 8.9, 8.8 and 11.1 nm, as determined from the BJH analysis of N_2_ adsorption‐desorption isotherms (Supporting Information Figures S8–9).

We also previously characterized meso‐TiO_2_/Fe_2_O_3_ using scanning transmission electron microscopy energy‐dispersive X‐ray spectroscopy (STEM‐EDS), finding that the Fe_2_O_3_ phase of meso‐TiO_2_/Fe_2_O_3_ is present both as a thin surface coating (i. e. <10 nm thickness) and as more discrete surface‐bound nanoparticle structures.^[45]^ Low energy ion scattering (LEIS) revealed that Fe_2_O_3_ covered 68±1 % of the surface, whilst only 32±1 % was TiO_2_.

Previous analysis of the near‐surface using X‐ray photoelectron spectroscopy (XPS) showed Ti(IV) with binding energies of 459.14 and 464.90 eV for Ti2p3/2 and Ti2p1/2 respectively and Fe(III) with binding energies of 711.46 and 724.81 eV for Fe2p3/2 and Fe2p1/2 respectively.^[45]^ No other oxidation states were found for these transition metals (i. e. no Fe(II) with Fe2p3/2 at 722.6 eV and Fe2p1/2 at 709.0 eV)^[54]^ in agreement with characterization of the bulk material using XRD.

### Improved total arsenic removal under environmental conditions using the composite photocatalyst‐adsorbent approach for a one‐step treatment

**Figure 1:**
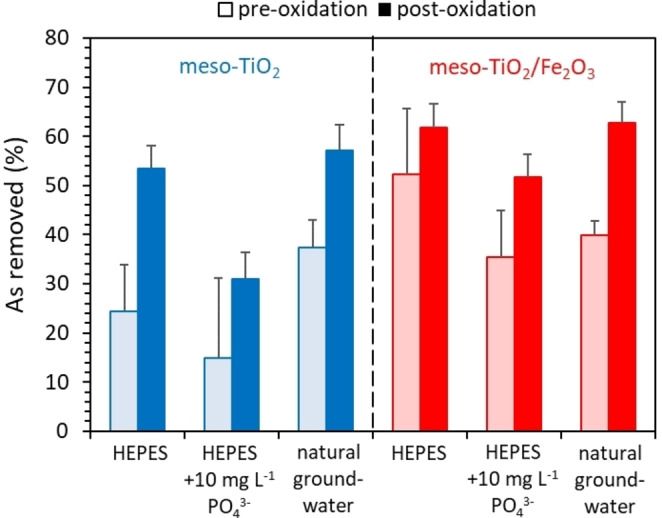
Comparison of total arsenic removal before (light bars) and after (dark bars) photooxidation, using meso‐TiO_2_ (blue) and meso‐TiO_2_/Fe_2_O_3_ (red) photocatalysts in a variety of media. The experimental conditions were 1 mg L^−1^ initial As(III) (adsorbed in the dark overnight), 0.1 g L^−1^ catalyst loading, pH 7.3±0.1. Meso‐TiO_2_ suspensions were irradiated for a minimum of 30 minutes, and meso‐TiO_2_/Fe_2_O_3_ suspensions for a minimum of 120 minutes, after which <20 μg L^−1^ aqueous As(III) remained (i. e. ≥98 % oxidation). Error bars indicate the uncertainty calculated from the standard error between 2 and 3 repeat ASV measurements.

Under environmentally relevant conditions (1 mg L^−1^ total As, and with 10 mg L^−1^ phosphate or in natural groundwater) (1) photocatalytic oxidation significantly improves total arsenic removal, and (2) addition of the Fe_2_O_3_ sorbent phase to the meso‐TiO_2_ photocatalyst improves total arsenic removal (Figure 1). These effects are cumulative. We previously found that the adsorption capacity of meso‐TiO_2_/Fe_2_O_3_ is comparable with that of the commercial goethite (FeOOH) sorbent *Bayoxide E33*.^[45]^ These increases in total arsenic removal highlight the potential for the composite photocatalyst‐sorbent system to improve As(III) remediation by combining oxidation capabilities with high As(V) adsorption capacities in a single‐step treatment.

### Quantifying parasitic light absorption by Fe_2_O_3_ using UV‐Vis spectroscopy and component additivity

**Figure 2:**
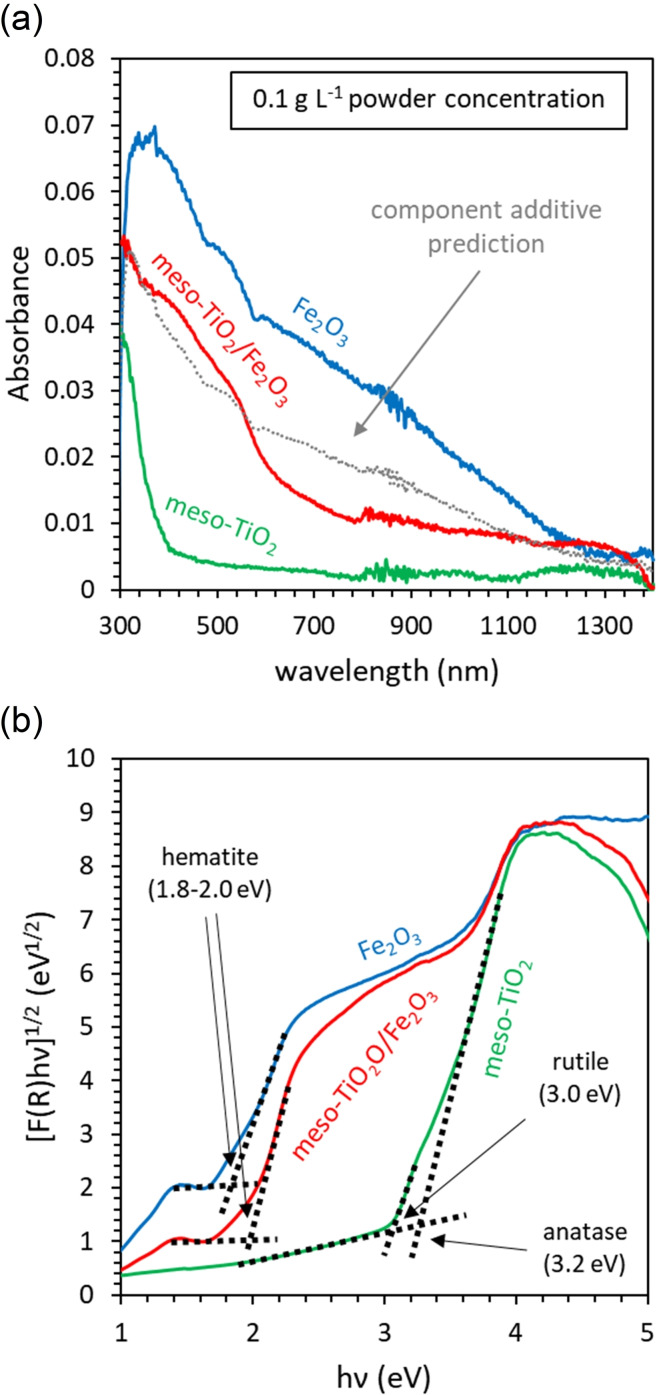
UV‐Vis spectroscopy used to evaluate parasitic light absorption. (a) UV‐Vis absorbance of meso‐TiO_2_, meso‐TiO_2_/Fe_2_O_3_, and Fe_2_O_3_ powders (0.1 g L^−1^, suspended in Milli‐Q water). The dashed grey‐line indicates the component additive prediction (using the meso‐TiO_2_ and Fe_2_O_3_ spectra as end‐members, weighted according to the XRF mass ratio). The path length was 2 mm. (b) Bandgap determination using diffuse reflectance spectra of dry powders (converted to F(R) using the Kubelka‐Munk function). The bandgap was identified by extrapolating the steep linear regions of the Tauc plot to where the background absorption is intercepted.

The UV‐Vis absorption spectra of powders suspended in water are presented in Figure 2a. Fe_2_O_3_ shows a characteristic absorption band edge from 600 nm to lower wavelengths. Meso‐TiO_2_ shows a sharper band edge, characteristic of this material, from around 380 nm to lower wavelengths. The UV‐Vis absorbance spectra of meso‐TiO_2_/Fe_2_O_3_ is dominated by the influence of Fe_2_O_3_. The single‐component Fe_2_O_3_ sample shows a broad absorption from 600 nm to 1200 nm due to residual scattering not captured by the integrating sphere. This scattering is not present in the other samples and indicates that larger particles are present in the Fe_2_O_3_ sample^[55]^ in agreement with SEM and DLS analysis.^[45]^

The bandgaps of meso‐TiO_2_, meso‐TiO_2_/Fe_2_O_3_ and Fe_2_O_3_ powder samples were estimated using a Tauc plot.^[56]^ As absorption could not be determined from the dry powders, F(R), a parameter that is proportional to the extinction coefficient, was determined from diffuse reflectance data using the Kubelka‐Munk function.^[57]^ An exponential power of 1/2 was used in the Tauc plot given that anatase TiO_2_ has an indirect allowed bandgap^[58]^ whilst Fe_2_O_3_ shows both direct and indirect transitions at nearly equivalent energies.^[59]^ The bandgap of the majority anatase phase within meso‐TiO_2_ is 3.2 eV, though a weaker absorbance corresponding to the minority rutile phase is also seen at 3.0 eV (Figure 2b) (cf. literature values of 3.03 and 3.20 eV respectively).^[60]^ The only bandgap that can be identified in the Tauc plot of composite meso‐TiO_2_/Fe_2_O_3_ is that of Fe_2_O_3_ at 2.0 eV (cf. literature values of 1.9–2.2 eV).^[61]^

A linear combination of the UV‐Vis absorbance spectra recorded for meso‐TiO_2_ and Fe_2_O_3_ [weighted 44 % and 56 % respectively as per XRF analysis, Equation (2)] successfully predicts the absorbance curve and extinction coefficient of meso‐TiO_2_/Fe_2_O_3_ at wavelengths shorter than 540 nm (Figure 2a), suggesting component additivity. Using Equation (3), the component additive prediction of the absorption coefficient ϵ is 2.2 g^−1^ cm^−1^ for meso‐TiO_2_/Fe_2_O_3_ (at λ=368 nm and 0.1 g L^−1^), which is marginally greater than the experimentally observed 2.0 g^−1^ cm^−1^. The ultraviolet absorption of composite meso‐TiO_2_/Fe_2_O_3_ can therefore be treated as the mass‐weighted linear combination of non‐interacting meso‐TiO_2_ and Fe_2_O_3_ components.

Using Equation (4), component additivity predicts that only 12 % of incident photons are absorbed by the meso‐TiO_2_ component at λ=368 nm (the wavelength of the UV lamp used in our photocatalysis experiments). The remaining 88 % of incident photons are absorbed by the Fe_2_O_3_ component. This is an important finding, given that (a) single‐component Fe_2_O_3_ shows negligible activity towards the photocatalytic oxidation of As(III) compared with TiO_2_ ^[18]^ and (b) the transfer of charge carriers from Fe_2_O_3_ to TiO_2_ is energetically unfavourable (see following section). The Fe_2_O_3_ phase is therefore anticipated to parasitically absorb the majority of incident light without promoting the oxidation of As(III). To the author's knowledge, this is the first time that the simple approach of mass‐weighted component additivity has been used to approximate parasitic light absorption within composite photocatalysts for As(III) oxidation.

### Identifying the role of the TiO_2_/Fe_2_O_3_ heterojunction using transient absorption spectroscopy (TAS) and component additivity

**Figure 3:**
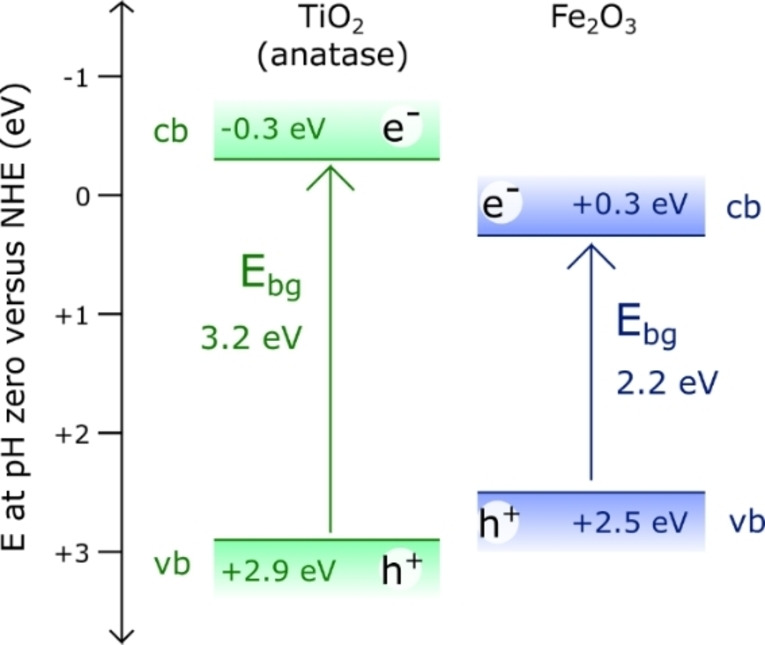
Band alignment of the anatase TiO_2_ and hematite Fe_2_O_3_ phases within the meso‐TiO_2_/Fe_2_O_3_ composite photocatalyst. Band energies are from the literature.^[64]^

The conduction band of anatase TiO_2_ is more negative than that of Fe_2_O_3_ (ca. −0.3 eV versus ca. +0.3 eV) whilst the valence band of anatase TiO_2_ is more positive than that of Fe_2_O_3_ (ca. +2.9 eV versus ca. +2.5 eV).^[62]^ As shown in Figure 3, these crystal phases form a straddling gap (type‐I) heterojunction, wherein the transfer of both electrons and holes from TiO_2_ to Fe_2_O_3_ is thermodynamically favourable.^[63]^ It was therefore important to assess whether charge transfer across the heterojunction of meso‐TiO_2_/Fe_2_O_3_ does indeed occur, given the potential enhancement in photocatalytic activity that this effect can generate.^[64]^

**Figure 4:**
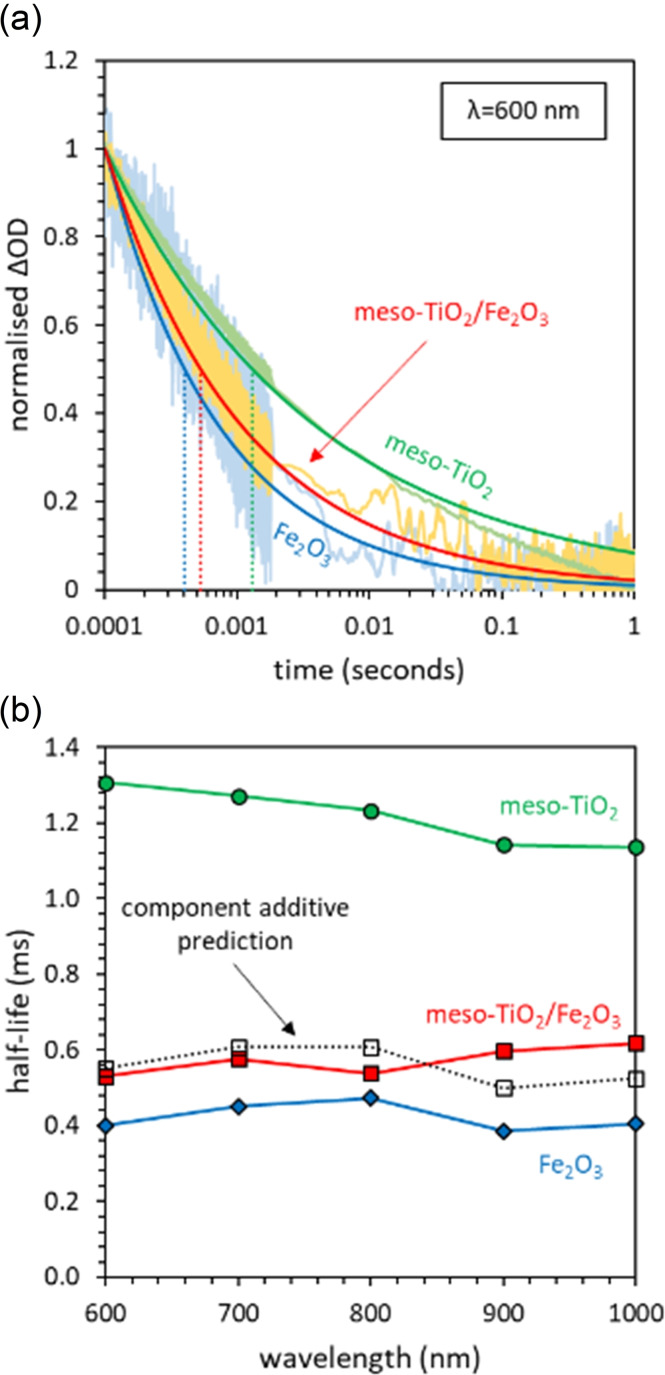
Identifying the role of the heterojunction formed in the composite meso‐TiO_2_/Fe_2_O_3_ photocatalyst using transient absorption spectroscopy (TAS) to probe charge carrier lifetimes. (a) Transient decay kinetics (with an example shown for the 600 nm probe) showed power‐law decays in all materials and at all probe wavelengths (λ=600–1000 nm), (b) A comparison of the transient absorption half‐lives observed experimentally with the half‐lives of meso‐TiO_2_/Fe_2_O_3_ predicted by component additivity (using meso‐TiO_2_ and Fe_2_O_3_ as end‐member reference samples).

Typical transient absorption decays for meso‐TiO_2_, Fe_2_O_3_ and the meso‐TiO_2_/Fe_2_O_3_ composite are presented in Figure 4a, with the full spectrum of wavelengths presented in Supporting Information Figure S23. Assuming component additivity, the product of the optical density and mass fraction of each component [Eq. (4)] estimates that 37 % of the absorbed laser pulse is absorbed by the meso‐TiO_2_ component, and that the remaining 63 % is absorbed by Fe_2_O_3_.

The linear combination of normalized transient absorption decays, weighted according to these results [Eq. (8)], closely predicts the experimentally observed transient absorption decay of meso‐TiO_2_/Fe_2_O_3_ across all wavelengths (Figure 4b). The charge carrier life‐times within meso‐TiO_2_/Fe_2_O_3_ (as a dry powder) are therefore no different than the life‐times expected for a mixture of non‐interacting meso‐TiO_2_ and Fe_2_O_3_ powders. This indicates that despite there being a thermodynamic driving force for the charge carriers formed in meso‐TiO_2_ to transfer into Fe_2_O_3_ and spatially separate (Figure 3), meso‐TiO_2_/Fe_2_O_3_ charge carrier lifetimes are no longer than those of single‐component meso‐TiO_2_ and Fe_2_O_3_ parent materials. Consequently, the TiO_2_‐Fe_2_O_3_ heterojunction within meso‐TiO_2_/Fe_2_O_3_ is not expected to improve photocatalytic activity. This is attributed to (i) the high parasitic absorption of light by Fe_2_O_3_ (∼63 %), whose charge carriers must overcome a high thermodynamic barrier to transfer into meso‐TiO_2_ and spatially separate, and (ii) limited interfacial contact between Fe_2_O_3_ and TiO_2_ phases, as indicated by our SEM images (Supporting Information Figure S5).

Whilst the charge carrier life times of titania‐iron oxide composites have been previously studied and modelled using TAS,^[65]^ to our knowledge, this is the first time that the component additive prediction of charge carrier lifetimes using single‐component reference samples has been used to identify the role of the heterojunction.

### Photocatalytic oxidation kinetics and the effect of coupling TiO_2_ with Fe_2_O_3_

#### The influence of light intensity on the initial rate of As(III) photocatalytic oxidation

**Figure 5:**
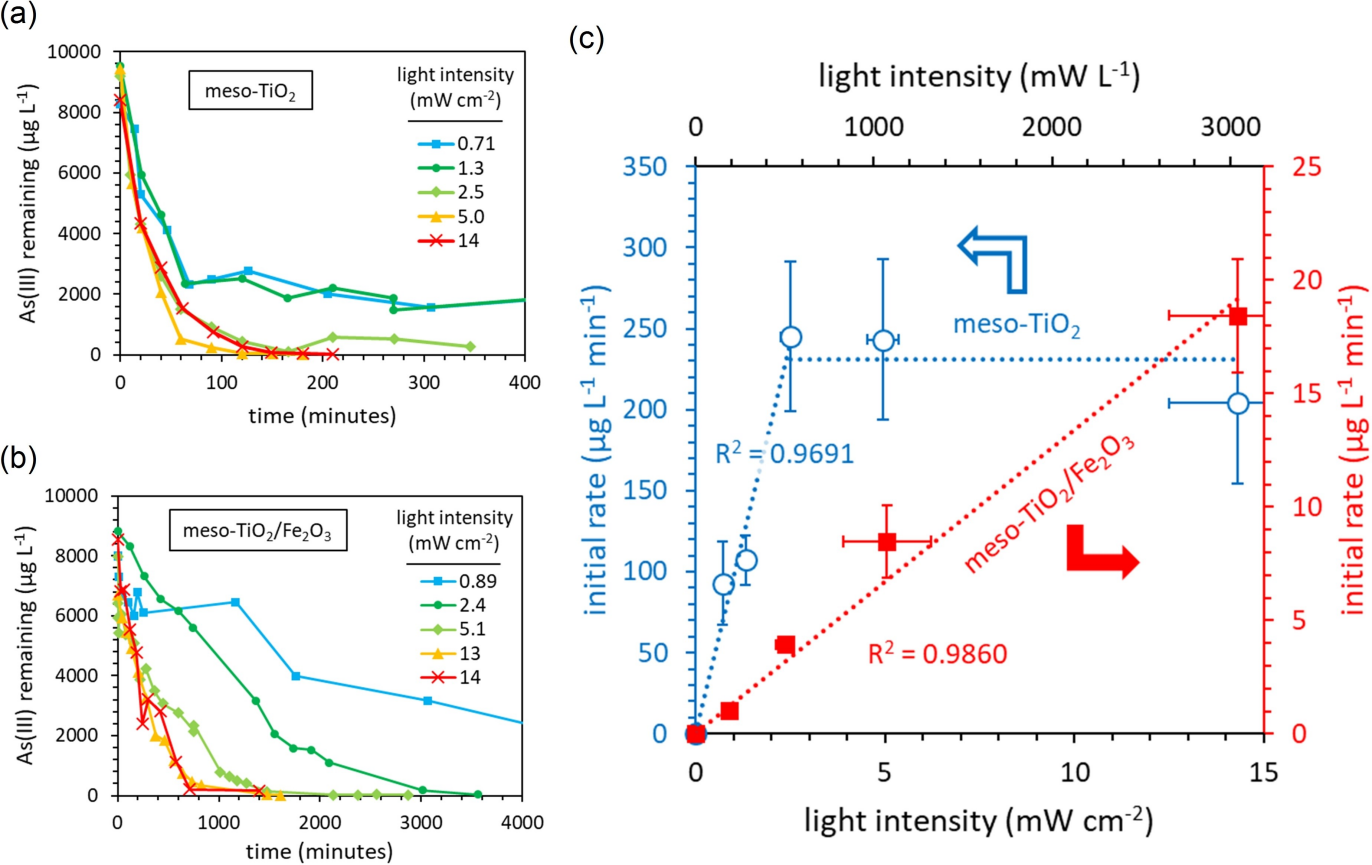
The influence of light intensity on the initial rate of As(III) photocatalytic oxidation in the presence of meso‐TiO_2_ and meso‐TiO_2_/Fe_2_O_3_ photocatalysts. The light intensity was varied by increasing and decreasing the distance between the UV lamp (λ=368 nm) and the photoreactor surface. The experimental conditions were 10 mg L^−1^ initial As(III), 0.1 g L^−1^ photocatalyst, 10 mM HEPES (pH 7.3±0.1), 14 mW cm^−2^ light intensity (λ=368 nm) and 100 mL total volume. Error bars indicate the standard deviation in the slope of the linear regression used to calculate initial rates. The average uncertainty in the calculated concentration of As(III) is 10 %.

Single‐component meso‐TiO_2_ and composite meso‐TiO_2_/Fe_2_O_3_ show different responses to changes in light intensity (Figure 5). A linear relationship between light intensity and the initial rate of As(III) oxidation is observed for meso‐TiO_2_/Fe_2_O_3_. In contrast, the meso‐TiO_2_ system initially shows a first‐order relationship between light intensity and the initial rate between 0 and 2.5 mW cm^−2^, after which the initial rate becomes independent of light intensity. Whilst this typically indicates mass transport limitations, we find that the As(III) concentration has no effect upon the initial rate (see following section). It therefore remains unclear whether the zero‐order relationship after 2.5 mW cm^−2^ is due to increased electron‐hole recombination or mass transport limitations. Similar results were observed by Dutta et al. (using 0.1 g L^−1^ Degussa P25 TiO_2_), with the slope of reaction rate as a function of light intensity decreasing at 6 mW cm^−2^.^[48]^ Dutta et al. found that overall the rate of reaction was proportional to I^0.23^, where I is the light intensity, raising possibilities of both increased electron‐hole recombination and mass transport limitations at high light intensity.

The oxidation of As(III) did not reach completion when using meso‐TiO_2_ at the lowest light intensities (0.71–1.3 mW cm^−2^). This could be a result of photocatalyst deactivation caused by the adsorption of As(V), as explored later. In contrast, all reactions proceeded to completion when using meso‐TiO_2_/Fe_2_O_3_, suggesting that the presence of a sorbent phase prevents catalyst deactivation, as proposed by Vaiano et al.^[66]^ The quantum yield (Φ) indicates the number of reactions completed per incident photon. At 2.5 mW cm^−2^ light intensity, the quantum yield of As(III) oxidation is greater when using meso‐TiO_2_ than when using meso‐TiO_2_/Fe_2_O_3_, with a 68 times difference (Φ=3.9±0.9 % versus Φ=0.057±0.017 % respectively). As light intensity is increased beyond 2.5 mW cm^−2^, this difference in quantum efficiency decreases: the quantum yield of meso‐TiO_2_ decreases with increasing light intensity (Φ=0.57±0.6 % at 14.3 mW cm^−2^) whilst the quantum yield of meso‐TiO_2_/FeO_3_ is maintained (Φ=0.051±0.010 % at 14.0 mW cm^−2^). UV‐Vis absorption spectroscopy shows that due to parasitic absorption by Fe_2_O_3_, the meso‐TiO_2_ component of the composite photocatalyst only absorbs 12 % of the total ultraviolet photons. Consequently, the relative rate of electron‐hole recombination within the TiO_2_ phase of meso‐TiO_2_/Fe_2_O_3_ at 14 mW cm^−2^ is theoretically equivalent to that of meso‐TiO_2_ with just 1.7 mW cm^−2^, which is within the linear range of initial rates versus light intensity. The linear relationship between light intensity and the initial reaction rate therefore extends to higher values of light intensity for the composite photocatalyst than pure meso‐TiO_2_, due to parasitic light absorption.

The difference in oxidation rates is too great to be explained by parasitic light absorption alone (discussed in detail later). We consequently investigated the kinetic mechanisms of both photocatalyst systems to identify whether Fe_2_O_3_‐coupling affects the reaction pathway of As(III) photooxidation. Initial rates were used to determine the order of reaction, and data at later times was then used to understand the role of As(V) catalyst deactivation and adsorption‐controlled kinetics.

#### The reaction order determined from initial rates

**Figure 6:**
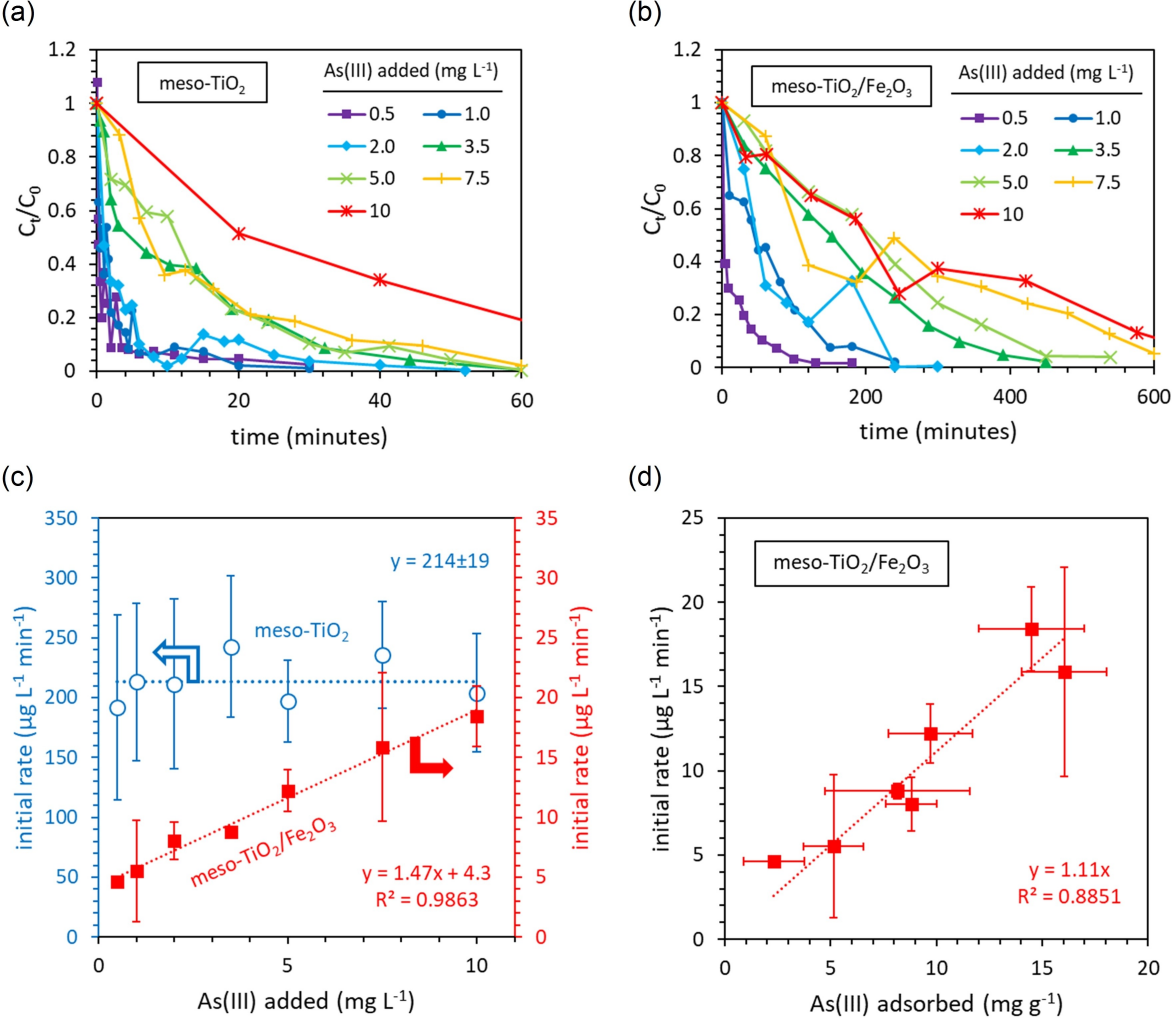
The influence of initial As(III) concentrations on photocatalytic oxidation kinetics in the presence of (a) meso‐TiO_2_ and (b) meso‐TiO_2_/Fe_2_O_3_ photocatalysts. (c) The influence of As(III) concentration on the initial rate. (d) The influence of adsorbed As(III) on the initial rate. The experimental conditions were 0.5–10 mg L^−1^ As(III), 0.1 g L^−1^ photocatalyst, 10 mM HEPES (pH 7.3±0.1), 14 mW cm^−2^ light intensity (λ=368 nm), and 100 mL total volume. Error bars indicate the uncertainty in the initial rate determined as the standard deviation of the slope in the linear regression fitted to the initial experimental kinetics. The average uncertainty in the calculated concentration of As(III) was 10 %.

Meso‐TiO_2_ and meso‐TiO_2_/Fe_2_O_3_ responded differently to changes in the concentration of As(III) (Figure 6). In the presence of meso‐TiO_2_, the initial rate was independent of the As(III) concentration (0.5–10 mg L^−1^ As(III) added). Zero‐order kinetics were also observed by Dutta et al. using TiO_2_.^[48]^ Zero‐order kinetics can be caused by saturation of the catalyst surface.^[67]^ However, our previous work found that at pH 7.0±0.1, monolayer coverage is incomplete when [As(III) (aq)]<10 mg L^−1[45]^ as per this study. Consequently, the observed zero‐order kinetics indicate that the rate determining step is linked to another process, such as the generation of holes, electrons or reactive oxygen species (ROS) intermediates.^[67]^ Phosphate can suppress photocatalysis reaction rates through competitive adsorption and surface‐site blocking, or enhance rates through the promotion of hydroxyl radical formation.^[40]^ The addition of phosphate increased meso‐TiO_2_ reaction rates and did not interfere with the charge scavenging behaviour of As(III) (Supporting Information Figure S14) suggesting that the rate is determined by the production of ROS, and not the availability of As(III).^[40]^

For meso‐TiO_2_/Fe_2_O_3_, a linear relationship is observed between the initial rate and the concentration of As(III) (Figure 6c). This relationship is imperfect due to an offset from the origin by a positive y‐intercept. Consequently, the system approximates pseudo‐first order (PFO) kinetics at high As(III) concentrations. In contrast, the first‐order relationship observed between the initial rate and the concentration of adsorbed As(III) is valid even at low As(III) concentrations (Figure 6d). This indicates that unlike meso‐TiO_2_, adsorbed As(III) is involved in the rate determining step when using meso‐TiO_2_/Fe_2_O_3_. The addition of phosphate (a) decreases As(III) adsorption and (b) suppresses reaction rates (Supporting Information Figure S14), providing further evidence that the kinetics of meso‐TiO_2_/Fe_2_O_3_ are controlled by the concentration of adsorbed As(III).^[40]^

#### Kinetic data at later times

**Figure 7:**
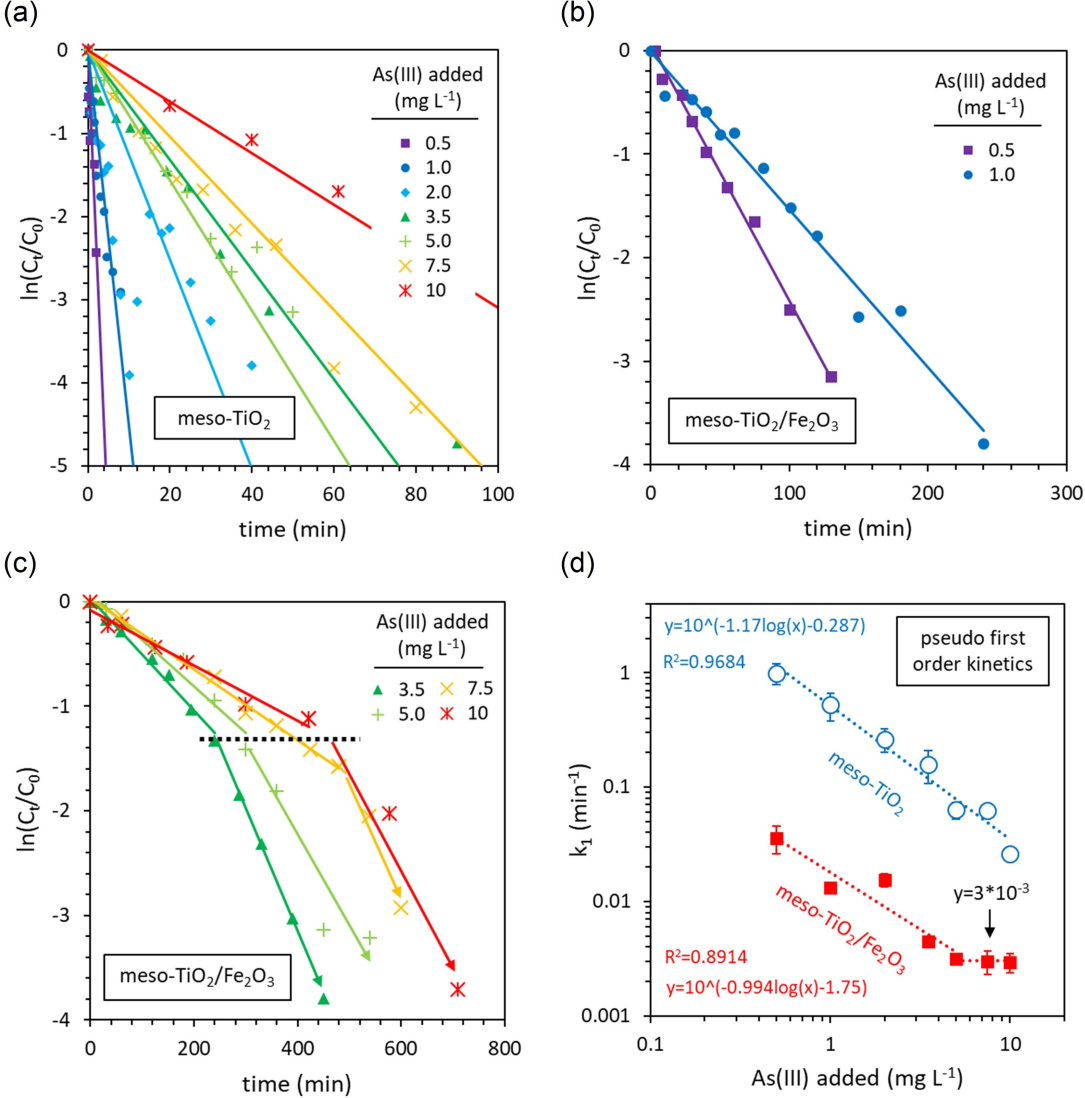
Linearisation of data at later times using pseudo first‐order kinetics. The successful linearisation of (a) meso‐TiO_2_, and (b) meso‐TiO_2_/Fe_2_O_3_ with 0.5 and 1 mg L^−1^ initial As(III). (c) The failed linearisation of meso‐TiO_2_/Fe_2_O_3_ data with ≥3.5 mg L^−1^ As(III). (d) The influence of As(III) concentrations on the PFO rate constant, k_1_. All values of k_1_ were calculated from the initial linear region of ln(C_t_/C_0_) versus time. The average R^2^ values of the linear regression of the PFO plots were 0.9327±0.075 for meso‐TiO_2_ and 0.9747±0.0297 for initial linear region for meso‐TiO_2_/Fe_2_O_3_.

Whilst initial rates established a zero‐order relationship between the concentration of As(III) and meso‐TiO_2_ photocatalytic oxidation kinetics (see previous section), the data at later times cannot be described by the zero‐order kinetic model as in all cases the reaction rate slows as As(III) is depleted, being best fit using a pseudo‐first order (PFO) kinetic model (Figure 7a). The combined analysis of initial rates and data at later times reveals that meso‐TiO_2_ follows disguised zero‐order kinetics.^[67]^

When using meso‐TiO_2_/Fe_2_O_3_, data at later times also disagrees with the analysis of initial rates. PFO linearization is successful when less than 2 mg L^−1^ As(III) is added (Figure 7b). However, linearization fails with more than 2 mg L^−1^ As(III), since the slope of ln(C_t_/C_0_) versus time increases in magnitude when ln(C_t_/C_0_)≈−1.5 (Figure 7c). This contradicts the analysis of initial rates which suggests that PFO kinetics should be best approximated at higher As(III) concentrations, rather than at low As(III) concentrations. The curvature in ln(C_t_/C_0_) as a function of time can also be observed in the study of Ferguson et al. (3.2 mg L^−1^ initial As(III) and Degussa P25 TiO_2_).^[50]^ In their study, no curvature was observed at lower initial As(III) concentrations of 0.19 mg L^−1^, similar to our results.

The influence of the As(III) concentration on the PFO rate constant (k_1_) is presented in Figure 7d. For meso‐TiO_2_, k_1_ decreases exponentially with increasing As(III) concentrations, in‐line with disguised zero‐order kinetics.^[67]^ For meso‐TiO_2_/Fe_2_O_3_, k_1_ decreases exponentially as the As(III) concentration is increased, up to 5 mg L^−1^, whereupon k_1_ stabilizes. With a stable k_1_ at high As(III) concentrations, this trend contradicts the failure of PFO kinetics to linearize data at later times (Figure 7c) but agrees with the analysis of initial rates in the previous section.

The following discussion reconciles data at later times with initial rates analysis by developing new rate equations to provide rate constants that are consistent across the 0.5–10 mg L^−1^ As(III) concentration range.

#### Disguised zero‐order kinetics of meso‐TiO_2_ explained by photocatalyst deactivation

The method of initial rates is widely considered to reveal the true reaction order, whilst data at later times can ‘disguise’ the true rate law.^[67]^ The true rate law will satisfy two conditions: (1) the initial rate is not influenced by the concentration of As(III), and (2) the absolute reaction rate decreases as oxidation progresses. Vaiano et al. reported deactivation of TiO_2_ due to the adsorption of As(V), reducing photocatalytic activity.^[41]^ We similarly identified incomplete oxidation of As(III) using meso‐TiO_2_ with low light intensities (Figure 5a). We therefore considered deactivation of meso‐TiO_2_ due to the presence of As(V) as an explanation for disguised zero‐order kinetics.

We developed and explored two new rate equations that depend upon the concentration of As(V). The first model uses C_0_ and C_t_ parameters, i. e. aqueous phase parameters only. This model is presented and discussed in Supporting Information section 6 and goes some way towards reconciling initial rates and data at later times (Supporting Information Figure S19). However, photocatalyst deactivation due to the presence of As(V) is likely to be caused by adsorbed As(V) blocking the solid‐solution interface.[^40^, ^41^] We therefore also constructed a rate law where only adsorbed As(V) decreases the reaction rate. The concentration of adsorbed As(V) was estimated using a surface complexation model (SCM) developed in our previous work.^[45]^ This model predicts adsorption as a function of pH and ionic strength, and crucially incorporates competitive adsorption, for example As(V) versus As(III). We considered the simple case that the reaction rate decreases linearly with the increasing concentration of adsorbed As(V), arriving at Equation S17. This rate equation is named the “SCM‐constrained As(V) model”.

**Figure 8:**
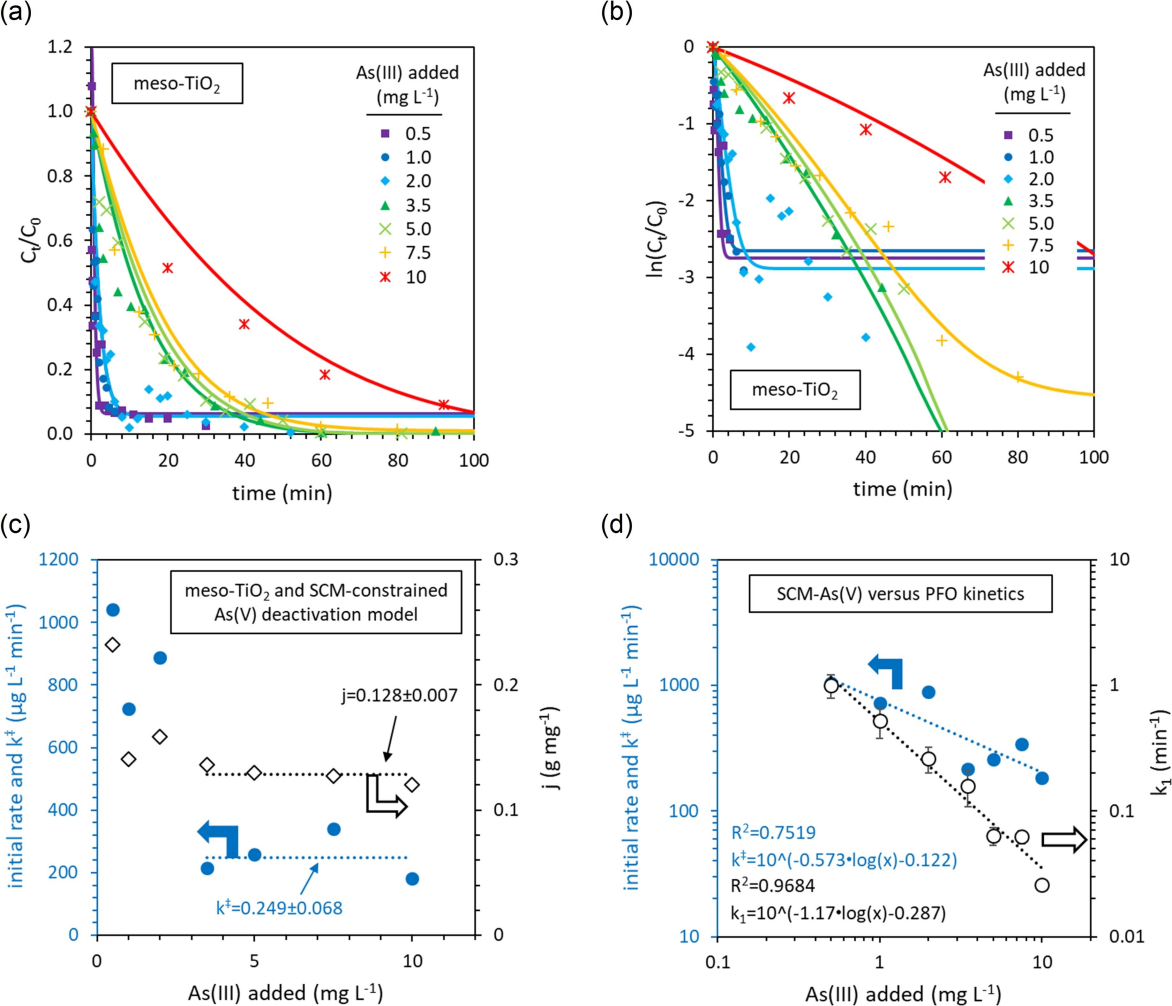
SCM‐constrained As(V) deactivation kinetics for the photocatalytic oxidation of As(III) in the presence of meso‐TiO_2_. (a, b) Comparison of the fitted model and experimental data. (c) The influence of the As(III) concentration on the initial rate, the rate constant k_≠_, and the sensitivity factor j. (d) Values of k^≠^ calculated from experimental data are less influenced by [As(III)] than the PFO rate constant k_1_.

The results of this model are presented in Figure 8. Firstly, the PFO shape of the data at later times is reproduced using this model (Figure 8a). It is only at the end of each reaction that PFO linearization fails (Figure 8b). This is because once the concentration of adsorbed As(V) reaches $\frac{1}{j}$ , the rate falls to zero and the reaction ceases. Secondly, when the initial concentration of As(III) ≥3.5 mg L^−1^, the initial rate is independent of the As(III) concentration (Figure 8c), providing the zero‐order kinetics identified by initial rates analysis. Within this concentration range, the sensitivity parameter, j, is also constant (0.131±0.009 g mg^−1^, with the reaction ceasing once the concentration of adsorbed As(V) reaches 7.6±0.5 mg g^−1^). The sensitivity of meso‐TiO_2_ to deactivation by As(V) adsorption is therefore independent of the concentration of As(III). This model fails when the initial concentration of As(III) is <3.5 mg L^−1^, since the rate constant k^≠^ increases with decreasing As(III) concentrations. Importantly, this As(V) deactivation model provides a rate constant that is significantly less dependent upon the initial concentration of As(III) than the PFO rate constant k_1_ (Figure 8d). The success of this preliminary model to reconcile initial rates and data at later times indicates that the disguised zero‐order kinetics of meso‐TiO_2_ are caused by As(V) deactivation of the catalyst.

Since initial rates are independent of the concentration of As(III) (Figure 6c) and the addition of phosphate enhances reaction rates (Supporting Information section 6), we suggest that the deactivation of meso‐TiO_2_ due to adsorbed As(V) is caused by As(V) interfering with the generation of ROS intermediates, rather than by As(V) blocking the access of As(III) to the catalyst surface.

#### First‐order kinetics of meso‐TiO_2_/Fe_2_O_3_ explained by the late stage partitioning of As(III) to the adsorbed phase

Langmuir‐Hinshelwood (LH) kinetics are often used to describe adsorption‐controlled catalysis,^[68]^ however, to our knowledge, only a couple of papers have considered LH kinetics for the photocatalytic oxidation of As(III).[^51^, ^52^] The LH rate equation is first‐order with respect to the concentration of adsorbed As(III), as calculated using the Langmuir adsorption isotherm model.^[68]^ LH kinetics successfully introduced some of the curvature in ln(C_t_/C_0_) observed for meso‐TiO_2_/Fe_2_O_3_ when the initial concentration of As(III) ≥3.5 mg L^−1^ (Supporting Information Figure S17). However, we encounter two problems: (1) the initial rates do not conform to linearized LH kinetics, and (2) the rate constant (k_LH_) decreases exponentially with increasing initial As(III) concentrations. The Langmuir‐Hinshelwood kinetic model notably does not consider interference by competitor ions,^[68]^ such as the As(V) that appears to deactivate meso‐TiO_2_.

**Figure 9:**
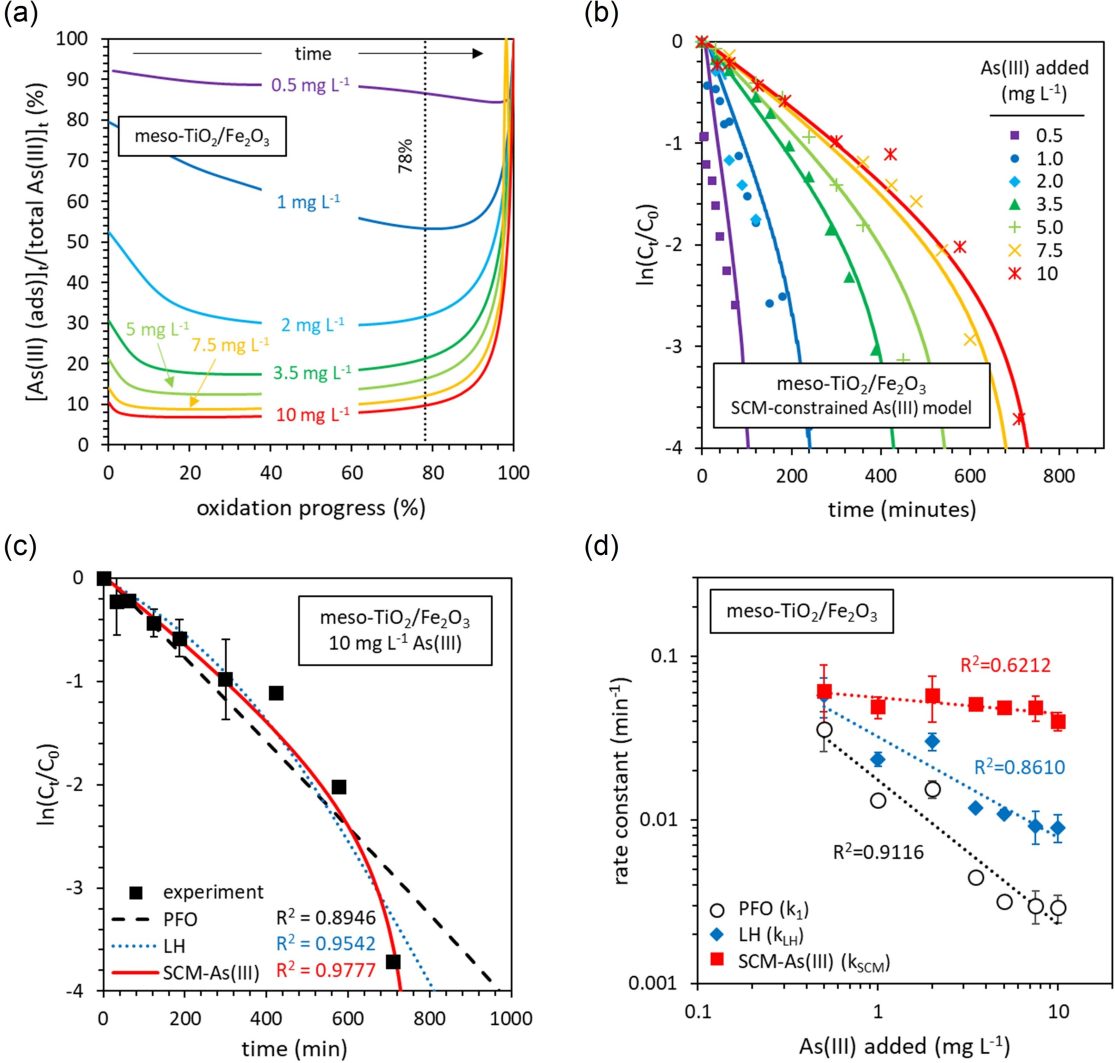
Explaining the kinetics of As(III) photocatalytic oxidation in the presence of meso‐TiO_2_/Fe_2_O_3_ using the SCM‐constrained As(III) kinetic model. (a) The SCM‐predicted partitioning of As(III) between adsorbed and aqueous phases, with different total arsenic concentrations, as photocatalytic oxidation progresses. The 78 % line is equivalent to ln(C_t_/C_0_)≈−1.5. The catalyst concentration was 0.1 g L^−1^, and the SCM methodology is presented in the Supporting Information. (b) The SCM‐constrained As(III) kinetic model fitted to data at later times in the form ln(C_t_/C_0_). (c) A comparison of models fitted to data at later times in the form lnC_t_/C_0_). (d) The influence of As(III) concentration on the rate constants calculated for each model.

We subsequently used surface complexation modelling (SCM) to investigate how the concentration of adsorbed As(III) changes as the photooxidation reaction progresses, using discrete TiO_2_ and Fe_2_O_3_ surface phases and including the competitive adsorption of As(V). This model predicts a significant increase in the partitioning of As(III) to the adsorbed phase as the final 20 % of As(III) is oxidized (Figure 9a). This coincides with the increased steepness in the linearized PFO plots of experimental data at ln(C_t_/C_0_)≈−1.5 (Figure 7c), suggesting that the experimental oxidation kinetics are explained by the partitioning of As(III) between aqueous and adsorbed phases.

We therefore modified the Langmuir‐Hinshelwood kinetic model so that the concentration of adsorbed As(III) is calculated using the SCM, thereby incorporating the competitive adsorption of As(V) (Eq. S18 and Figure 9b). Our new SCM‐constrained model best captures the observed curvature in plots of ln(C_t_/C_0_) versus time (Figure 9c). Langmuir‐Hinshelwood kinetics provide only some of the observed curvature, since whilst the Langmuir adsorption isotherm predicts increased partitioning of As(III) to the surface as oxidation progresses, there is no sharp feature after 78 % of As(III) has been oxidized (Supporting Information Figure S18). The SCM‐constrained model also greatly reduces the conditionality of the rate constant towards the initial As(III) concentration, compared with the PFO and LH kinetic models (Figure 9d). These results indicate that the photocatalytic oxidation kinetics of meso‐TiO_2_/Fe_2_O_3_ are controlled by the adsorption of As(III), competing with As(V) for surface sites.

#### A comparison of different mechanisms of As(III) oxidation

A valid rate equation will yield a rate constant that is independent of the initial As(III) concentration, i.e [Eq. (1)],

(1) $$\frac{\Delta log\left(k\right)}{\Delta log\left(\left[As\left(III\right)\;added\right]\right)}=0$$

**Figure 10:**
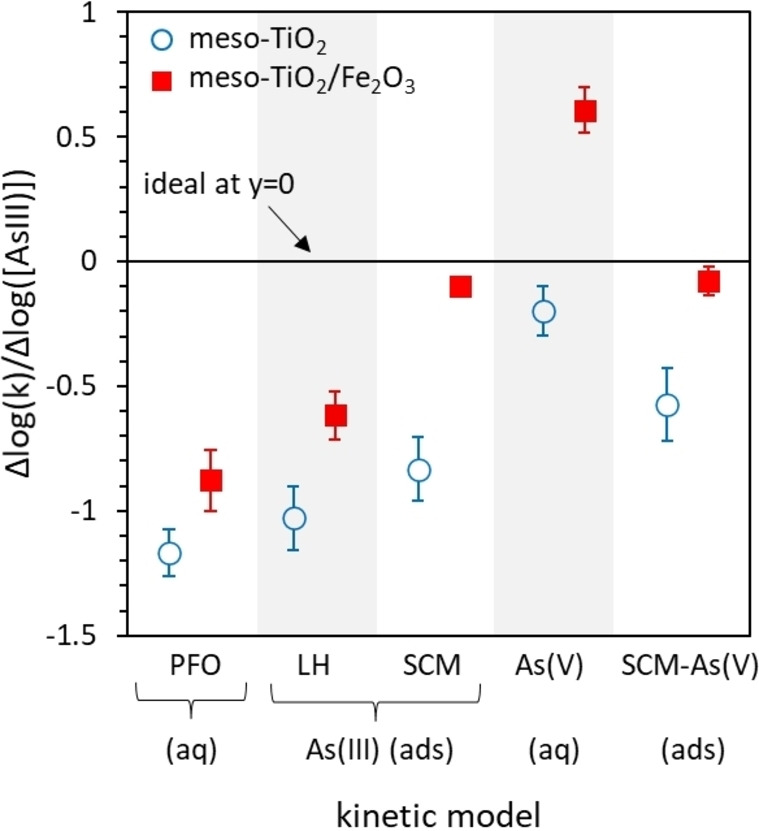
Rate constant dependence upon initial As(III) concentrations, for each kinetic model investigated in this work. Accurate rate equations will provide a rate constant that is independent of [As(III)], and thus the closer to zero that $\frac{\Delta log\left(k\right)}{\Delta log([As\left(III\right)]}$ is, the more valid the kinetic model is.

For meso‐TiO_2_, the As(V) deactivation model recreates the experimentally observed disguised zero‐order kinetics and yields the most consistent rate constants (Figure 10). Consistent rate constants cannot be obtained for meso‐TiO_2_ using (a) PFO kinetics or (b) As(III) adsorption‐controlled kinetics (e. g. LH kinetics). This mechanism is consistent with phosphate experiments, which suggest that the oxidation of As(III) proceeds via ROS intermediates (Supporting Information Figure S14), and that it is the generation of these ROS intermediates that determines the reaction rate.

In contrast, the most consistent rate constants for meso‐TiO_2_/Fe_2_O_3_ are achieved using the SCM‐constrained As(III) and As(V) adsorption models (Figure 10). Of these, only the SCM‐constrained As(III) adsorption rate equation gives the first‐order relationship between the concentration of adsorbed As(III) and initial rates that is observed experimentally (Figure 6d).

Despite possessing a greater adsorption capacity (and significant parasitic absorption, presumably decreasing the rate of ROS generation), the kinetics of meso‐TiO_2_/Fe_2_O_3_ depend on [As(III) (ads)], whilst the kinetics of meso‐TiO_2_ do not. To explain this, we consider the speciation of arsenic adsorbed onto the TiO_2_ and Fe_2_O_3_ surfaces of the composite photocatalyst. The SCM achieves the best fit to experimental data when covalently bonded, inner‐sphere (>SO)_2_AsOH is the major surface complex for Fe_2_O_3_‐sorbed As(III) at pH 7^[45]^ (where >S denotes the metal oxide surface). In contrast, meso‐TiO_2_ is best fit using a mix of inner‐sphere (>SO)_2_AsO^−^ and the weakly bound outer‐sphere (hydrogen‐bonded) >SOH_2_^+^–AsO(OH)_2_^−^ surface complex. Equilibrium constants for these reactions are greater for Fe_2_O_3_ (logK=5.3) than for meso‐TiO_2_ (logK=3.0 and 4.0 respectively). The As(III) surface complexes formed on the TiO_2_ surface are therefore weaker than those formed on the Fe_2_O_3_ surface, and TiO_2_‐sorbed As(III) is therefore more rapidly substituted for As(V) than Fe_2_O_3_‐sorbed As(III).

**Figure 11:**
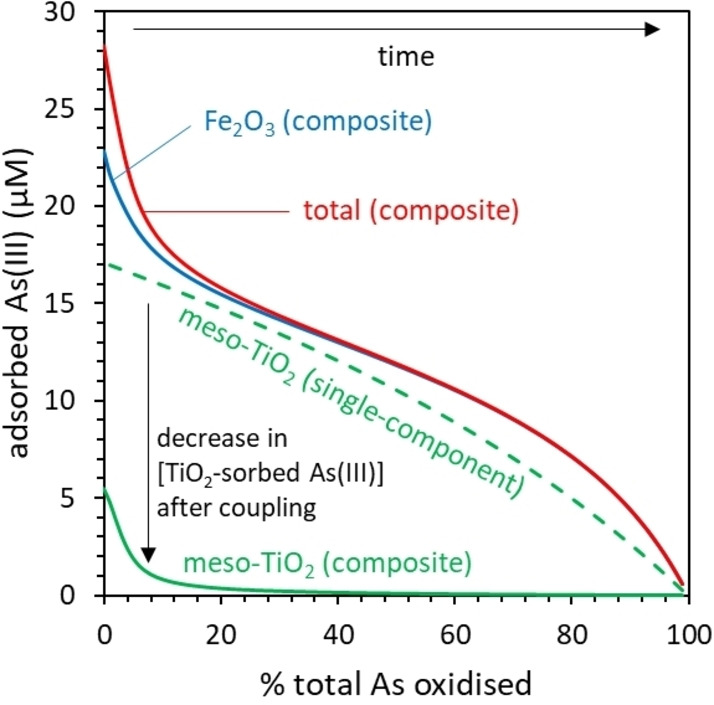
Changes in the concentration of adsorbed As(III) with progressive oxidation, predicted using a component additive surface complexation model (CA‐SCM). The conditions are 10 mg L^−1^ initial As(III), 0.1 g L^−1^ photocatalyst, 0.01 M NaCl, pH 7.

Consequently, whilst meso‐TiO_2_ shows an approximately linear decrease in the concentration of TiO_2_‐sorbed As(III) with increasing reaction progress, As(III) adsorbed on the minority TiO_2_ surface phase of meso‐TiO_2_/Fe_2_O_3_ is rapidly replaced by As(V) (Figure 11). The model predicts that after 10 % of all As(III) has been oxidized, the concentration of TiO_2_‐sorbed As(III) is twenty times less in the meso‐TiO_2_/Fe_2_O_3_ system than the meso‐TiO_2_ system (with an initial As(III) concentration of 10 mg L^−1^). After 20 % of all As(III) has been oxidized, this difference increases to a factor of 40. Consequently, whilst the kinetics of single‐component meso‐TiO_2_ depend principally upon the generation of ROS, the availability of adsorbed As(III) becomes a rate limiting factor for the meso‐TiO_2_/Fe_2_O_3_ system (Figure 11).

Despite the SCM predicting that the As(III) adsorbed on the TiO_2_ surface is rapidly substituted for As(V), meso‐TiO_2_/Fe_2_O_3_ does not show signs of As(V) deactivation: the reaction proceeds to completion in Figure 5b, and the initial rates are best described by the As(III) adsorption model rather than the As(V) deactivation model. Whilst the SCM predicts that the Fe_2_O_3_ sorbent phase does not prevent TiO_2_ from becoming saturated with As(V), the sorbent phase may prevent catalyst fouling by maintaining a high concentration of adsorbed As(III) at the overall composite surface. For instance, in the meso‐TiO_2_/Fe_2_O_3_ system, there is 23 times more As(III) adsorbed on Fe_2_O_3_ versus TiO_2_ after 10 % oxidation, increasing to a factor of 43 after 20 % oxidation (Figure 11). The surface diffusion of ROS intermediates has been reported (with distances of up to tens of microns)^[69]^ raising the possibility that ROS generated by the TiO_2_ phase migrates to oxidize the more prevalent Fe_2_O_3_‐sorbed As(III).

### Concept for the photocatalytic oxidation of As(III) using meso‐TiO_2_/Fe_2_O_3_

**Figure 12:**
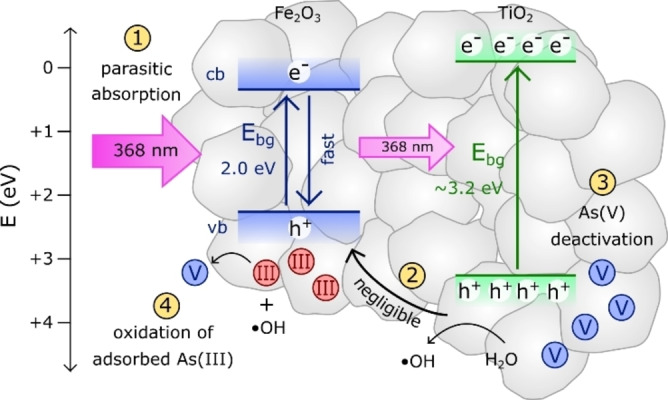
Concept schematic illustrating the photocatalytic oxidation of As(III) by meso‐TiO_2_/Fe_2_O_3_. (1) The majority of incident photons (88 % at 368 nm) are parasitically absorbed by the Fe_2_O_3_ component. (2) Despite being thermodynamically favourable, the transfer of positive holes from the valence band of meso‐TiO_2_ to Fe_2_O_3_ is insignificant. (3) The adsorption of As(V) deactivates the photocatalyst. (4) Reactive oxygen species (ROS) such as the hydroxyl radical oxidise adsorbed As(III), the majority of which is found adsorbed to the Fe_2_O_3_ surface. As(III) is represented by red circles and As(V) by blue circles.

Figure 12 shows a schematic model illustrating the photocatalytic oxidation of As(III) by meso‐TiO_2_/Fe_2_O_3_. Firstly, the majority of incident photons (88 % at 368 nm) are parasitically absorbed by the Fe_2_O_3_ component with insignificant oxidation of As(III).^[18]^ Secondly, despite being thermodynamically favourable, charge transfer from meso‐TiO_2_ to Fe_2_O_3_ is insignificant, with the TiO_2_‐Fe_2_O_3_ heterojunction prepared under this synthesis method failing to enhance photocatalysis. Thirdly, the adsorption of As(V) deactivates the photocatalyst, principally by preventing the generation of reactive oxygen species (ROS) intermediates. Finally, due to the low availability of As(III) adsorbed on the TiO_2_ surface, ROS potentially migrates to the Fe_2_O_3_ phase before inducing the oxidation of Fe_2_O_3_‐sorbed As(III). In this model, it appears that incorporation of the Fe_2_O_3_ sorbent phase does not prevent photocatalyst deactivation by suppressing the adsorption of As(V) onto the TiO_2_ surface,^[66]^ but rather by maintaining a local concentration of As(III) on the neighbouring Fe_2_O_3_ surface.

The relative importance of these mechanisms can be evaluated semi‐quantitatively. When light power ≤2.5 mW cm^−2^ the difference in reaction rates is greatest (∼60 times) whilst the TiO_2_ electron‐hole recombination rates in meso‐TiO_2_ and meso‐TiO_2_/Fe_2_O_3_ are equivalent. Firstly, the component‐additive TAS model developed in this work indicates that the TiO_2_−Fe_2_O_3_ heterojunction provides zero increase to the rate of As(III) oxidation. In contrast, UV‐Vis absorption spectroscopy indicates that parasitic light absorption accounts for 88 % of the observed difference in reaction rates. After considering parasitic light absorption, a 7.5±1.4 factor difference in oxidation rates remains unexplained. Photocatalytic activity is often linked to surface area^[70]^ and we previously identified that only 32±1 % of the exposed meso‐TiO_2_/Fe_2_O_3_ surface is meso‐TiO_2_.^[45]^ Assuming a linear relationship between surface area and photocatalytic activity, the iron oxide surface coating would account for up to 68 % of the difference in reaction rates. After considering possible surface area effects, a 2.4±0.5 factor difference in rates remains unaccounted for and is therefore attributed to the different reaction mechanisms, where the availability of adsorbed As(III) becomes a limiting factor once meso‐TiO_2_ is coupled with Fe_2_O_3_ (as indicated by our application of surface complexation modelling). Consequently, parasitic absorption is likely the principle mechanism determining photocatalytic oxidation rates in the TiO_2_/Fe_2_O_3_ composites, with the change in rate law acting as a secondary influence.

## Conclusion

This study investigated, for the first time, the mechanisms by which coupling a TiO_2_ photocatalyst with an iron oxide sorbent phase influences reaction kinetics in the photocatalytic oxidation of As(III). Our key finding is that oxidation kinetics are decreased after sorbent‐coupling, primarily due to parasitic light absorption by the Fe_2_O_3_ sorbent phase, and secondarily due to changes in the rate law, from being limited by the generation of ROS intermediates (disguised zero‐order kinetics) to being limited by the availability of adsorbed As(III) (first‐order kinetics). Moreover, our TAS studies show that the TiO_2_‐Fe_2_O_3_ heterojunction formed by this synthesis method does not effectively separate charge carriers.

To evaluate how material coupling influences As(III) oxidation kinetics, we developed new hybrid experimental‐modelling approaches and our key innovations are: (i) The quantification of parasitic absorption using UV‐Vis spectroscopy and component additivity; (ii) Evaluation of the role of the heterojunction using a combination of transient absorption spectroscopy (TAS) and component additivity; (iii) The first application of a multi‐sorbate surface complexation model (SCM) to constrain competitive adsorption‐controlled kinetics more accurately than the single‐sorbate Langmuir‐Hinshelwood model; and (iv) The first application of a As(V)‐deactivation kinetic model to explain disguised zero‐order kinetics in the photocatalytic oxidation of As(III). These approaches should help understand the influence of mineral coupling in other composite photocatalyst systems, and the SCM‐constrained kinetic model should be used in place of Langmuir‐Hinshelwood kinetics when modelling As(III) oxidation due to the competitive adsorption of As(V).

Current sorbent media (e. g. iron oxides) could be readily substituted with meso‐TiO_2_/Fe_2_O_3_, maintaining high arsenic removal capacities whilst imparting modest photocatalytic capabilities. This may be advantageous for single‐step, single‐reactor treatments, simplifying construction and maintenance of treatment plants^[46]^ given that maintenance is a major reason for the failure of current treatment plants.^[15]^ However, when it is necessary to improve oxidation kinetics (and thus energy efficiency), it will be important to reduce the parasitic light absorption of the sorbent phase, for example replacing Fe_2_O_3_ with a wide bandgap sorbent such as aluminum oxide (Al_2_O_3_, 6 eV)^[71]^ which is already used as an arsenic sorbent.^[15]^ Potentially, TiO_2_ (bandgap 3.0‐3.2 eV) can be substituted for photocatalysts such as graphitic carbon nitride (g‐C_3_N_4_, 2.7 eV) which not only absorbs visible light, but is also a poor arsenic sorbent and therefore should not suffer from the same As(V) deactivation as TiO_2_.[^51^, ^72^] In the case of titania‐iron oxide composites, alternative synthesis routes and composite architectures (e. g. Janus particles) should be investigated to achieve charge carrier separation across the heterojunction.^[73]^ Whilst efficient removal of As(V) using meso‐TiO_2_/Fe_2_O_3_ after 9 regeneration cycles has been demonstrated previously,^[28]^ future engineering studies should also investigate potential changes to photocatalytic oxidation kinetics after regeneration of the photocatalyst, and correlate any changes in the kinetics to material properties (e. g. surface characterization using SEM and XPS).[^74^, ^75^]

## Experimental Section

### Materials and reagents

All chemicals were reagent or analytical grade (as detailed in Supporting Information Table S1). Stock solutions of As(III) and As(V) (1000 mg L^−1^) were prepared from As_2_O_3_ and Na_2_HAsO_4_ ⋅ 7H_2_O respectively and stored in opaque plastic bottles, refrigerated at 3 °C to prevent changes in the speciation. Solutions of 10 mM HEPES were prepared from HEPES free acid (powder). Groundwater was collected from an arsenic‐contaminated area of West Bengal, India as described in our previous work^[76]^ and characterized in Supporting Information section 1.2.

### Synthesis of materials

Meso‐TiO_2_ and meso‐TiO_2_/Fe_2_O_3_ were synthesized following the procedure of Zhou et al.^[28]^ as per our previous work.[^45^, ^46^] First, meso‐TiO_2_ was prepared via a sol‐gel procedure using P123 as a structure‐directing agent: a solution of ethanolic P123 (1 g in 12 mL) was added dropwise to Ti(IV) n‐butoxide in concentrated HCl (2.7 g in 3.2 g). After stirring to evaporate the solvent under ambient conditions, the sol‐gel product was calcined at 350 °C for four hours and crushed. Secondly, meso‐TiO_2_ was coupled with Fe_2_O_3_, aiming to give a 1 : 1 mass ratio of TiO_2_ to Fe_2_O_3_: meso‐TiO_2_ (1.5 g) was added to a solution of 0.60 M ethanolic iron nitrate (30 mL) and after evaporating the solvent at 50 °C with stirring, the product was calcined at 300 °C for six hours and crushed. A Fe_2_O_3_ reference sample was prepared similarly, albeit without addition of meso‐TiO_2_.

### Materials characterisation

The crystal phases of meso‐TiO_2_/Fe_2_O_3_ and the reference samples were identified by X‐ray diffraction (XRD) using a PANalytical MPD. Crystallite diameters were estimated from the peak broadening using the Scherrer equation (Supporting Information equation S1). The BET‐specific surface area and pore size were determined using Brunauer‐Emmett‐Teller (BET) and Barrett‐Joyner‐Halenda (BJH) analysis respectively, from N_2_ adsorption‐desorption isotherms measured using a Quantachrome Autosorb iQ. All samples were outgassed at 200 °C for 20 h prior to analysis. The elemental composition was determined by X‐ray fluorescence (XRF) using a PANalytical Epsilon 3 XLE, and the mass ratio between meso‐TiO_2_ and Fe_2_O_3_ components was calculated as described in Supporting Information section 2.4.

### Component additive analysis of UV‐Vis absorbance

UV‐Vis spectra were recorded using a Shimadzu UV‐2700 spectrophotometer, equipped with an integrating sphere, and analysed using the Beer‐Lambert law and the Kubelka‐Munk function as described in Supporting Information section 1.5.

To quantify the extent of parasitic absorption, a component additive prediction of absorbance (A) was calculated for the meso‐TiO_2_/Fe_2_O_3_ composite photocatalyst, using a linear combination of the data collected for meso‐TiO_2_ and Fe_2_O_3_ reference samples, weighted according to the mass fraction of each component:

(2) $$A_{comp}=m_{a}A_{b}+{m_{b}A}_{b}$$

where *comp* denotes the composite photocatalyst, *a* and *b* denote the reference samples (i. e. meso‐TiO_2_ and Fe_2_O_3_), A_comp_ is the predicted absorbance of the composite photocatalyst, m_i_ is the mass fraction of component *i* within the composite photocatalyst (calculated using XRF), and A_i_ is the experimentally determined absorbance of the single‐component reference sample *i*.^[77]^

The extinction coefficient of the composite photocatalyst, ϵ_comp_, was calculated similarly:

(3) $$\epsilon_{comp}={m_{a}\epsilon }_{a}+m_{b}\epsilon_{b}$$

where ϵ_i_ is the experimentally calculated extinction coefficient of the single‐component reference sample *i* (L g^−1^ cm^−1^).

The proportion of incident photons absorbed by each component (meso‐TiO_2_ or Fe_2_O_3_) within the composite was then estimated using the equation:

(4) $$\theta_{i}\;=\frac{m_{i}A_{i}}{A_{comp}}$$

where θ_i_ represents the fraction of the total photons (at the given wavelength) absorbed by the composite photocatalyst that are absorbed by component *i*, and A_i_ is the absorbance (or extinction coefficient) of component *i*.

### Transient absorption spectroscopy (TAS)

TAS spectra were recorded as described in Supporting Information section 1.6. Treating meso‐TiO_2_ and Fe_2_O_3_ phases within the composite photocatalyst as non‐interacting components, a component additive prediction was calculated to assess the possibility of charge transfer across the TiO_2_−Fe_2_O_3_ heterojunction. First, to determine transient absorption half‐lives more accurately, transient absorption decay curves were modelled using a power‐law decay function with the equation:

(5) $${\left[h^{+}\right]}_{t}=At^{-\alpha }$$

where [h^+^]_t_ is the concentration of holes at time t (represented by the change in optical density, ΔOD), t is time (seconds), and A and α are fitting parameters.^[78]^ Parameters A and α were optimized to improve the goodness of fit (R^2^) between modelled and experimental data. Data before 0.1 and after 100 ms were excluded from data fitting, due to interference from the laser pulse and loss of material suspension respectively.

TAS spectra were normalized to unity at t=100 μs, with the normalized value of A calculated via the rearrangement of Equation (5), giving:

(6) $$A_{normalised}=\frac{1}{0.0001^{-\alpha }}$$

Transient absorption half‐lives (t_1/2_) were then calculated using rearrangement of Equation (5) to give:

(7) $$t_{1/2}={\left(\frac{1}{2}\cdot \frac{1}{A_{normalised}}\right)}^{-\frac{1}{\alpha }}$$

The component additive prediction of meso‐TiO_2_/Fe_2_O_3_ transient absorption decay was calculated using a linear combination of meso‐TiO_2_ and Fe_2_O_3_ power law decay functions. First, the distribution of the absorbed laser pulse at λ=355 nm between meso‐TiO_2_ and Fe_2_O_3_ components was estimated as the product of each component's optical density at λ=355 nm and its mass fraction (Equation (4), incorporating Equations S7 and S19). The component additive prediction of transient absorption decay was then calculated using the formula:

(8) $${\Delta OD\left(t,\lambda \right)}_{comp}={\theta \left(0,355\;nm\right)}_{a}{\Delta OD\left(t,\lambda \right)}_{a}+{{\theta \left(0,355\;nm\right)}_{b}\Delta OD\left(t,\lambda \right)}_{b}$$

where ΔOD(t,λ)_comp_ is the change in optical density (i. e. the transient absorption) of the composite photocatalyst at time t and wavelength λ, θ(0,355 nm)_*i*_ is the proportion of the laser pulse absorbed by component *i* (Equation (4)), and ΔOD(t,λ)_*i*_ is the experimentally observed transient absorption of component *i* at time *t* and wavelength *λ*.

### Photocatalytic oxidation kinetics

The photocatalytic oxidation of As(III) was measured in a 100 mL beaker with a diameter of 5.4 cm placed on top of a magnetic stirrer, illuminated overhead using a horizontal ultraviolet lamp (λ=368 nm, 18 mW). The light intensity across the sample surface area was measured using a power meter (PM100, Thorlabs) connected to a power sensor (S120UV, Thorlabs). The photoreactor was housed within an opaque black plastic box and a UV‐transparent fused‐silica lid was used to prevent evaporation. To vary the light power, the distance between the lamp and the sample was varied between 2.5 and 31 cm (0.7–14 mW cm^−2^). A diagram of this set‐up is presented in Supporting Information Figure S1.

Powders were suspended (0.1 g L^−1^) in 100 mL media (10 mM HEPES, with or without addition of 10 mg L^−1^ phosphate) and spiked with As(III) (0.5–10 mg L^−1^). Suspensions were adjusted to pH 7.3±0.1 through the addition of small volumes of 0.01 or 1 M HCl and NaOH, since the As(III) contaminated waters in South Asia are typically between pH 7 and 8.[^33^, ^34^, ^35^] Suspensions were magnetically stirred overnight in the dark to achieve equilibrium adsorption. The UV lamp was warmed up for ten minutes before the sample was irradiated. The time at which the suspension was first irradiated was designated t=0. At regular time intervals, 1 mL aliquots were extracted from the suspension and filtered using a 0.45 μm nylon membrane to remove solids. Samples were kept in the dark prior to analysis to prevent further photooxidation.

Concentrations of As(III) and total As were determined using anodic stripping voltammetry (ASV) and a method based upon established procedures,[^76^, ^79^, ^80^] described in detail in Supporting Information section 1.8. Oxidation progress was monitored using [As(III) (aq)] rather than [As(V) (aq)] as this is expected to give a more accurate measure of oxidation kinetics, especially at low total arsenic concentrations.^[81]^ The average uncertainty in the measurement was 10 %.

### Kinetic modelling

The kinetics of As(III) oxidation in the presence of meso‐TiO_2_ and meso‐TiO_2_/Fe_2_O_3_ were investigated using two methods for corroboration: the method of initial rates and analysis of data at later times.^[82]^ Initial rates were calculated by fitting a linear regression to the initial linear region observed when [As(III) (aq)] is plotted as a function of time. Data at later times was analysed using pseudo‐first order (PFO) kinetics, Langmuir‐Hinshelwood (LH) kinetics, a new As(V) deactivation model, and a new As(III) adsorption‐constrained kinetic model, which uses the surface complexation model (SCM) developed in a previous work.^[45]^ Full details of the modelling are presented in Supporting Information section 1.10.

### Total arsenic removal

Total arsenic removal experiments were prepared as per the kinetic experiments (0.1 g L^−1^ photocatalyst, pH 7.3±0.1, 100 mL volume), however only 1 mg L^−1^ As(III) was added. The background media was either 10 mM HEPES, 10 mM HEPES spiked with 50 mg L^−1^ PO_4_^3−^, or a natural groundwater sampled from an arsenic contaminated region of West Bengal. After stirring in the dark overnight to achieve equilibrium adsorption, the *pre‐oxidation* sample was collected. After photooxidation using ultraviolet light was complete, the *post‐oxidation* sample was collected (after a minimum of 30 minutes irradiation for meso‐TiO_2_ and a minimum of 120 minutes for meso‐TiO_2_/Fe_2_O_3_, after which <20 μg L^−1^ As(III) (aq) remained). The concentration of total As was determined using ASV, and total arsenic removal was calculated by subtracting the concentration of aqueous arsenic remaining from the concentration of arsenic initially added.

## Conflict of interest

The authors declare no conflict of interest.

1

## Supporting information

As a service to our authors and readers, this journal provides supporting information supplied by the authors. Such materials are peer reviewed and may be re‐organized for online delivery, but are not copy‐edited or typeset. Technical support issues arising from supporting information (other than missing files) should be addressed to the authors.

Supporting Information

## Data Availability

The data that support the findings of this study are openly available in Zenodo at 10.5281/zenodo.3975646, reference number 3975646.
