# Supplementary material for: Parasitic Light Absorption, Rate Laws and Heterojunctions in the Photocatalytic Oxidation of Arsenic(III) Using Composite TiO2/Fe2O3
Source: Chemistry. 2022 Feb 24;28(16):e202104181. doi: 10.1002/chem.202104181 (PMC9306794; doi:10.1002/chem.202104181)
Supplement: Supplementary file 1 — Supporting Information [file CHEM-28-0-s001.pdf]

Chemistry–A European Journal

Supporting Information

Parasitic Light Absorption, Rate Laws and Heterojunctions in the Photocatalytic Oxidation of Arsenic(III) Using Composite TiO_2/Fe_2O_3

Jay C. Bullen,* Hany F. Heiba, Andreas Kafizas, and Dominik J. Weiss*

Table of Contents

1. Experimental 2
1.1. Reagents 2
1.2. Sampling and chemical characterisation of natural groundwater 2
1.3. X-ray diffraction (XRD) and the Scherrer equation 3
1.4. Electron microscopy 3
1.5. UV-Vis absorption spectroscopy 3
1.6. Transient absorption spectroscopy (TAS) 5
1.7. Set-up and characterisation of photoreactor 5
1.8. Detection of arsenic using anodic stripping voltammetry (ASV) 6
1.9. Quantum yield calculations 7
1.10. Kinetic modelling 7
2. Materials characterisation 10
2.1. Confirmation of crystal phases using X-ray diffraction (XRD) 10
2.2. Transmission electron microscopy (TEM) 10
2.3. Scanning electron microscopy (SEM) 11
2.4. Quantifying the composition of meso-TiO_2/Fe_2O_3 using X-ray fluorescence (XRF) 13
2.5. N_2 adsorption-desorption isotherms for determination of the BET-specific surface area and BJH pore size 14
3. Set-up of photooxidation kinetic experiments 16
3.1. Ultraviolet absorbance by powder suspensions 16
3.2. Operating conditions and constraints 18
3.3. Control experiments 19
4. The influence of phosphate on the initial rate of As(III) photocatalytic oxidation 19
5. Langmuir-Hinshelwood kinetics 21
5.1. Adsorption isotherm fitting 21
5.2. Linearised Langmuir-Hinshelwood kinetics and initial rate analysis 21
5.3. Non-linear Langmuir-Hinshelwood kinetics 23
5.4. Langmuir adsorption isotherm prediction of As(III) distribution 23
6. As(V) deactivation kinetics: a simple model using C_0 and C_t parameters only 24
7. Surface complexation modelling (SCM) 26
7.1. SCM parameters 26
7.2. SCM-constrained kinetics versus pseudo-first order (PFO) and Langmuir-Hinshelwood (LH) kinetics 27
8. Transient absorption spectroscopy (TAS) 29
8.1. Component additivity of optical density 29
8.2. Transient absorption of dry powders 30
8.3. The influence of phosphate on transient absorption 32
9. References 33

1. Experimental

1.1. Reagents

Table S1: Reagents used in this work

Reagent	Name	Manufacturer	Grade/purity	CAS #
PEG-PPG-PEG	PEG Pluronic® P-123	Aldrich	-	9003-11-6
C_2H_5OH	Ethanol	VWR	ACS/Puriss p.a.	64-17-5
Ti(IV) n-butoxide	Titanium butoxide	ACROS Organics	99%	5593-70-4
HCl (conc)	Hydrochloric acid	ACROS Organics	ACS reagent grade ca. 37%	7647-01-0
$Fe(NO_3)_3.9H_2O$	Iron(III) nitrate nonahydrate	Sigma-Aldrich	ACS reagent grade, >98%	7782-61-8
$Na_2HAsO_4 \cdot 7H_2O$	Sodium arsenate dibasic heptahydrate	Sigma	ACS reagent	10048-95-0
As_2O_3	Arsenic trioxide	Aldrich	99%	1327-53-3
As (aq)	Arsenic standard	Fluka	1000 ± 4 mg L^{-1} traceCERT	-
1.0 M NaOH	Sodium hydroxide	Honeywell Fluka	-	1310-73-2
1.0 M HCl	Hydrochloric acid	Honeywell Fluka	-	7647-01-0
HNO_3	Concentrated nitric acid	-	Parboiled	7697-37-2
$C_8H_{18}N_2O_4S$	HEPES free acid	Amresco	High purity grade	7365-45-9
$HNa_2PO_4.7H_2O$	Sodium phosphate dibasic heptahydrate	Acros organics	99+% for analysis	7782-85-6
CH_3OH	Methanol	VWR	≥99.8%	67-56-1
$AgNO_3$	Silver nitrate	Sigma-Aldrich	≥99.0%	7761-88-8

1.2. Sampling and chemical characterisation of natural groundwater

The natural groundwater (*STN2*) was collected from a deep well in the village of Chakudanga (23°04'57.7"N 88°36'09.0"E) in West Bengal, India. The tube well was pumped for 6-15 minutes before sampling. The sample was preserved by acidifying to pH 2 with HCl. A partial characterisation of this sample is given in Table S2. Similar groundwaters collected from the same village are characterised in our previous work [1], displaying a pH of 7.2-8.4, and a UV-Vis absorbance at 254 nm of 0.022-0.046, suggesting the presence of humic acid-like dissolved organic matter. The pH of the acidified groundwater was adjusted to 7.3 ±0.1 with the addition of NaOH prior to the addition of As(III) and TiO_2.

Table S2: Partial chemical characterisation of the natural groundwater used in this work, before spiking with 1 mg L^{-1} As(III). The sample was acidified to approximately pH 2 with the addition of concentrated HCl during sampling but neutralised before the experiment. Each species was determined using ICP-MS and a multielement standard as described elsewhere [1].

Species	STN2 (West Bengal deep well)
Total As (μg L^{-1})	16.3 ± 5.2
Ca^{2+} (mg L^{-1})	22
Na^{+} (mg L^{-1})	1041
Mg^{2+} (mg L^{-1})	32
K^{+} (mg L^{-1})	4.3
Fe^{2+} (mg L^{-1})	4.8
Mn^{2+} (mg L^{-1})	0.49
Sr^{2+} (mg L^{-1})	0.20
Ba^{2+} (mg L^{-1})	0.17

1.3. X-ray diffraction (XRD) and the Scherrer equation

Crystallite diameters were estimated from XRD patterns using the Scherrer equation:

$$t = \frac{K\,\lambda}{\beta\cos(\theta)}$$

Equation S1

where t is the crystallite size (Å), K is the shape factor, λ is the X-ray wavelength (Å), β is peak broadening (i.e. the full width at half maximum, FWHM) in radians, and θ is the Bragg angle (°). The X-ray wavelength, λ, was set as the 50:50 average of the copper $K\alpha_1$ and $K\alpha_2$ lines (1.541874 Å). The shape factor was set to 0.94 (assuming spherical crystallites with cubic symmetry). Peak broadening due to the instrument was set as 0.09 (2°θ) and subtracted from the FWHM when determining β.

1.4. Electron microscopy

Powder samples were studied by scanning electron microscopy (SEM) using the LEO Gemini 1525 FEGSEM with 5.0 kV electron high tension (EHT) voltage. The constituent crystallites of powder samples were studied by transmission electron microscopy (TEM) using the JEOL JEM-2100F.

1.5. UV-Vis absorption spectroscopy

UV-visible transmission and reflection spectra were measured using a Shimadzu UV-2700 spectrophotometer, equipped with an integrating sphere, in the wavelength range of 190-1400 nm. Dry powders were measured in reflection mode, being held between glass slides, and referenced against $BaSO_4$. Powders suspended in Milli-Q water (0.01-10 g L^{-1} powder concentration) were measured in both transmission and reflection modes using a 0.1 or 0.2 cm cuvette (QC). Transmission and reflectance spectra were referenced against an air-filled cuvette. Reflectance and transmission spectra of water blanks were subtracted during data processing.

The absorbance of suspended powders was calculated using the proportion of incident light transmitted through the sample (including forward-scattered light captured by the integrating sphere):

$$A = -\log\left(\frac{I_t}{I_0}\right)$$

Equation S2

where I_t is the intensity of the transmitted light and I_0 is the intensity of the incident light. To account for diffuse reflection from the suspended powders (and back-scattered light captured by the integrating sphere), Equation S2 was modified to give the following:

$$A = -\log\left(\frac{I_t + I_r}{1 - I_{blank-r}}\right)$$

Equation S3

where I_t is the proportion of transmitted light, I_r is the proportion of reflected light, and $I_{blank-r}$ is the proportion of incident light reflected by the blank cuvette filled with water.

Extinction coefficients were determined using the Beer-Lambert Law:

$$A = \varepsilon cl$$

Equation S4

where A is absorbance (dimensionless); ε is the wavelength-dependent extinction coefficient (L g^{-1} cm^{-1}), c is the powder concentration (g L^{-1}); and l is the path length (cm) [2]. The Beer-Lambert law was then used to predict the proportion of incident photons at 368 nm absorbed as a function of solid concentration and path length.

For dry powders, the band-gap was determined using the Kubelka-Munk function:

$$F\left(R_\infty\right) = \frac{(1 - R_\infty)^2}{2R_\infty}$$

Equation S5

where R_∞ is the absolute reflectance of an infinitely thick layer of sample, i.e. the measured diffuse reflectance [3]. Photon energy, hν (eV), was calculated as:

$$h\nu = \frac{hc}{\lambda} \cdot 1.602 \cdot 10^{-19}$$

Equation S6

where h is Planck's constant (6.626x10^{-34} m^2 kg^{-1} s^{-1}), ν is frequency (s^{-1}), c is the speed of light (2.998x10^8 m s^{-1}), lambda is photon wavelength (m) and 1.602x10^{-19} is the conversion factor between joules and electron volts. A Tauc plot was used, with $(F(R_\infty)h\nu)^{1/2}$ as a function of hν, and the bandgap was identified by extrapolating the steep linear regions of the Tauc plot to where the background absorption is intercepted [4].

1.6. Transient absorption spectroscopy (TAS)

The experimental set-up and method was based upon that reported by Jiamprasertboon et al. [5], using a 355 nm laser, however several alterations were made. (1) The frequency of the laser pulse was increased to ~1 Hz. (2) The diffuse reflectance was measured between 600 and 1000 nm, in 100 nm increments. (3) Each kinetic trace was calculated from the average of 100 laser pulses, except for experiments in As(III) suspensions. Here, in order to minimise the systematic error of progressive As(III) photooxidation under the laser pulse, an average of 10 pulses was collected at each wavelength (in a randomised order), with triplicate measurements (a total of 30 pulses at each wavelength). (4) Samples were measured (a) in air, (b) in methanol and in 2 mM aqueous $AgNO_3$ and (c) in 10 mM HEPES (pH 7.3±0.1) with 0-50 mg L^{-1} As(III) and 0 or 50 mg L^{-1} phosphate.

The change in optical density (ΔOD) at time t, and wavelength λ, is related to the change in the intensity of transmitted or reflected light through the expression:

$$\Delta\mathrm{OD}(t,\lambda) = -\log\left(1 + \frac{\Delta I(t,\lambda)}{I_0(\lambda)}\right)$$

Equation S7

Where ΔOD(t, λ) is the change in optical density at time t, and at wavelength λ, ΔI is the change in the intensity of transmitted or reflected light at time t, and at wavelength λ, and I_0 is the intensity of transmitted or reflected light before the laser pulse [6].

1.7. Set-up and characterisation of photoreactor

The photoreactor was a 100 mL beaker located on top of a magnetic stirrer and illuminated overhead by a horizontal ultraviolet lamp (λ =368 nm, 18 mW). The design is illustrated in Figure S1. The light intensity across the sample surface area was measured using a power meter (PM 100, S120 UV, Thorlabs) connected to a power sensor (S120UV, Thorlabs). The photoreactor was housed within an opaque black plastic box and a UV-transparent fused-silica lid was used to prevent evaporation.

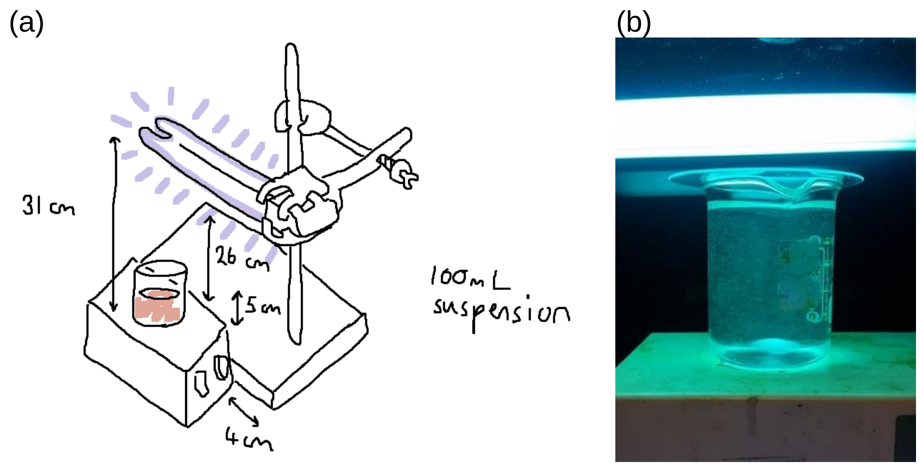

Figure S1: Photoreactor design. (a) To investigate the influence of photon flux on As(III) oxidation kinetics, the distance between the lamp and the suspension was adjusted using a retort stand. For all other experiments, the lamp was fixed 2 cm above the suspension surface. (b) Photograph showing the photocatalyst suspension, irradiated from above, stirred magnetically, and a fused-silica glass lid to prevent water loss due to evaporation.

The variance in the light intensity delivered by the lamp across the surface of the suspension was <1% (at the shorted distance of 2.5 cm) (Figure S2a). The power-law decrease in light intensity with the increasing distance between the lamp and the surface of the suspension after 2 cm (Figure S2b), indicates that the lamp can be approximated as a point source irradiating the suspension from above, ignoring lateral irradiation. This data was used to constrain the light intensity in the photocatalytic oxidation experiments. Initial experiments fixed the lamp in place with a retort stand, to facilitate adjustment of the distance between lamp and suspension, so that photon flux could be varied. When a power series had been established, the lamp was fixed in place (2 cm above the suspension surface).

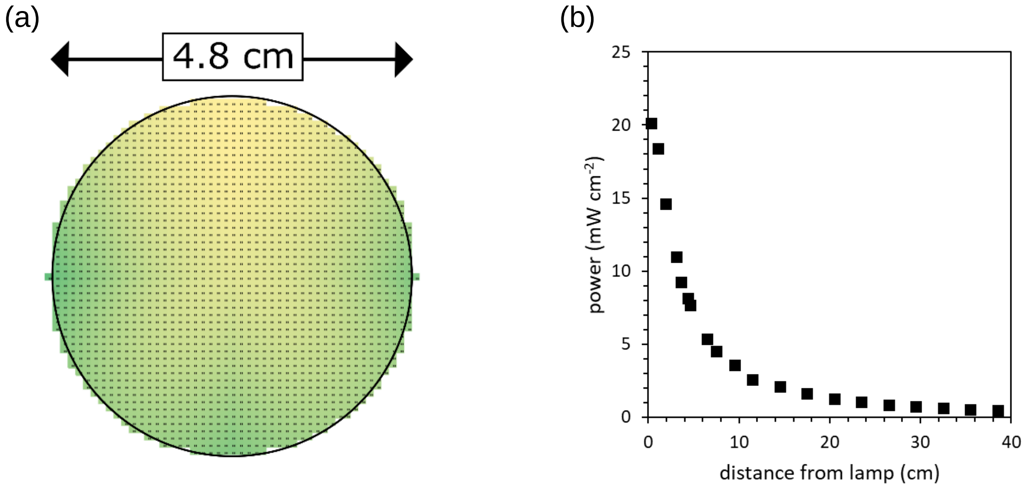

Figure S2: Characterisation of light intensity. (a) The light intensity was measured at five points across the surface of the suspension (four evenly spaced points along the circumference of the beaker and one in the centre) with the lamp located 2.5 cm above the beaker. The light intensity between these points was interpolated using a polynomial function, and the results suggested that the variance in light intensity was <1%. (b) The intensity of the light delivered to the suspension was measured as a function of the distance between the lamp and the surface of the beaker. Linear interpolation between data points was used to calculate the light intensity in each photocatalytic oxidation experiment.

1.8. Detection of arsenic using anodic stripping voltammetry (ASV)

As(III) and total As were determined by anodic stripping voltammetry (ASV) using the Metrohm 663 VA stand with the IME663 interface, and the General Purpose Electrochemical System (GPES) software. The procedure was modified from previously published works [1] [7]. The working electrode was a 25 µm diameter, 5 mm long gold microwire. Iridium wire and Ag/AgCl/KCl (3M) electrodes were used for the auxiliary and reference electrodes respectively.

The analytical scan consisted of 5 seconds conditioning at +0.7 V, 20 seconds deposition at -0.7 V (for the detection of As(III)) or -1.3 V (for the detection of total As) followed by a 1 second hold at -0.5 V. In the anodic stripping step, the potential was swept from -0.5 V to +0.7 V with a scan rate of 1 V s^{-1}. A square wave stripping profile was used, with a step potential of 8 mV, a square wave amplitude of 50 mV, and a frequency of 50 Hz. A background scan was made under identical conditions except for the deposition time, which was reduced to 1 second. The background scan was subtracted from the analytical scan before data processing.

As(III) was detected after the addition of 10 mM HCl and 40 µM hydrazine to prevent oxidation [8] [9]. Total As was detected after the addition of 0.1 M HCl, without hydrazine. The peak derivative was used

for quantification. Arsenic concentrations were determined using the method of standard additions, with a minimum of 2 additions of As(III) in each case and a minimum of three repeat scans between additions [10].

The working electrode was conditioned in 0.5 M H_2SO_4 at the start of each day by imposing a potential of -2.5 V for 30 seconds, followed by a cyclic voltammetry (CV) scan between -0.2 V and +1.5 V with a scan rate of 1 V s^{-1} [9].

1.9. Quantum yield calculations

The quantum yield (Φ) was calculated by dividing the incident light intensity by the initial rate. The calculation is expressed as:

$$\Phi\ (\%) = 100 \cdot \left(\frac{\mathrm{jSA}}{\mathrm{V}} \cdot \frac{\lambda}{\mathrm{hc}} \cdot \frac{1}{\mathrm{A}}\right) \div \left(\frac{\mathrm{r_i}}{\mathrm{M_{As}} \cdot 10^6}\right)$$

Equation S8

where j is the lamp power (J cm^{-2} s^{-1}), SA is the surface area of the beaker (cm^2), V is the volume of the beaker (L), λ is the wavelength of the incident photons (m), h is the Planck constant (6.63 x 10^{-3} m^2 kg^{-1} s^{-1}), c is the speed of light (3.00 x 10^8 m^2 s^{-1}), A is the Avogadro constant (6.02 x 10^{23} mol^{-1}), r_i is the initial rate of As(III) oxidation in µg L^{-1} s^{-1} (ranging between 0.02 and 4 µg L^{-1} s^{-1}) and M_{As} is the molar mass of As at 74.9 g mol^{-1}. In this study *j* was varied between 2.4 and 14.3 mW cm^{-2} (0.0024 to 0.0143 J cm^{-2} s^{-1}); SA was 21 cm^3; V was 0.1 L; and λ was 368 nm (3.68 x 10^{-7} m).

1.10. Kinetic modelling

The kinetics of As(III) oxidation in the presence of meso-TiO_2 and meso-TiO_2/Fe_2O_3 photocatalysts were investigated using two methods for corroboration: the method of initial rates and analysis of data at later times [11].

Initial rates were calculated by fitting a linear regression to the initial linear region observed when [As(III) (aq)] was plotted as a function of time. Data at later times was analysed using pseudo-first order, Langmuir-Hinshelwood, As(V) deactivation, and surface complexation model-constrained kinetic models.

Pseudo first-order (PFO) kinetics follow the rate equation:

$$\frac{\mathrm{dC}}{\mathrm{dt}} = -\mathrm{k_1 C_t}$$

Equation S9

where C is the concentration of aqueous As(III) (i.e. [As(III) (aq)]), t is time (min), k_1 is the PFO rate constant (min^{-1}) and C_t is the concentration of aqueous As(III) at time *t* [12]. The integrated solution to this equation gives the linear form:

$$\ln\left(\frac{\mathrm{C_t}}{\mathrm{C_0}}\right) = -\mathrm{k_1 t}$$

Equation S10

where C_0 is the concentration of aqueous As(III) at t=0. The PFO rate constant, k_1, was calculated from the slope of $\ln(C/C_0)$ as a function of time.

The Langmuir-Hinshelwood rate equation is first-order with respect to the concentration of adsorbed substrate (which is calculated using the Langmuir adsorption isotherm model). Given that a significant proportion of total As(III) is adsorbed to the photocatalyst surface at any given time, Langmuir-Hinshelwood kinetics were used to calculate the rate of change in the total concentration of As(III) remaining (rather than aqueous As(III) only), with the following rate equation:

$$\frac{d[\text{total As(III)}]}{dt} = k\frac{K_L C_t}{1 + K_L C_t}$$

Equation S11

where

$$[\text{total As(III)}]_t = [\text{As(III) (aq)}]_t + [\text{As(III) (ads)}]_t$$

Equation S12

and all concentrations take the units *mg L⁻¹*. k (mg L^{-1} min^{-1}) is the rate constant, and K_L is the Langmuir constant (L mg^{-1}) [13]. $[\text{As(III) (ads)}]_t$ is calculated at each point in time using the Langmuir adsorption isotherm:

$$[\text{As(III) (ads)}]_t = Q_{max} C_s \frac{K_L C_t}{1 + K_L C_t}$$

Equation S13

where Q_{max} is the monolayer adsorption capacity (mg g^{-1}) and C_s is the concentration of suspended photocatalyst (0.1 g L^{-1}). K_L and Q_{max} were calculated in this work by fitting the adsorption isotherms we reported previously [14] as demonstrated in the Supplementary Information (SI section 7.1).

The parameter *k* is a convolution of the Langmuir-Hinshelwood rate constant, k_{LH} (min^{-1}), and the monolayer adsorption capacity, Q_{max} (mg g^{-1}). The rate constant k_{LH} is thus obtained through the equation:

$$k_{LH} = \frac{k}{Q_{max} C_s}$$

Equation S14

Two different kinetic models were used to test possible As(V) deactivation of meso-TiO_2. The first model uses C_0 and C_t, the same aqueous phase parameters as PFO kinetics. The equation is:

$$\frac{dC}{dt} = k^{\dagger}\left(1 - \left(1 - \frac{C_t}{C_0}\right)\right)$$

Equation S15

where C is the concentration of aqueous As(III), $k^{\dagger}$ is the rate constant (μg L^{-1} min^{-1}), and C_t and C_0 are the concentrations of aqueous As(III) at time *t* and t=0 respectively. This equation reduces to:

$$\frac{dC}{dt} = k^{\dagger}\left(\frac{C_t}{C_0}\right)$$

Equation S16

The second model (the SCM-constrained As(V) model) uses the rate equation:

$$\frac{\mathrm{d[As(III)\ (total)]}}{\mathrm{dt}} = \mathrm{k}^{\ddagger}\left(1 - \mathrm{j}\frac{\mathrm{[As(V)\ (ads)]_t}}{\mathrm{C_s}}\right)$$

Equation S17

where $k^{\ddagger}$ is the rate constant (µg L^{-1} min^{-1}), j is a sensitivity factor reflecting the extent to which the presence of As(V) supresses the reaction (g mg^{-1}), and $[As(V)\ (ads)]_t$ is the concentration of adsorbed As(V) at time *t* (mg L^{-1}). The concentration of adsorbed As(V) at each point in time was calculated using a surface complexation model (SCM).

A SCM was previously developed for all materials used in this study [14]. New equilibrium constants for the competitive adsorption of phosphate were calculated using literature data. SCM calculations were performed using Visual MINTEQ 3.1. A further discussion of the method, as well as the SCM parameters used, is provided in the Supplementary Information (SI section 9.1).

The SCM was further used to model the [As(III) (ads)]-controlled kinetics of meso-TiO_2/Fe_2O_3, incorporating the competitive adsorption of As(V). The rate equation of the SCM-constrained As(III) model was:

$$\frac{\mathrm{d[total\ As(III)]}}{\mathrm{dt}} = \mathrm{k_{SCM}[As(III)\ (ads)]_t}$$

Equation S18

where k_{SCM} is the rate constant (min^{-1}), and $[As(III)\ (ads)]_t$ is the concentration of adsorbed As(III) at time *t* (mg L^{-1}), calculated by the SCM.

Values of k_{LH}, $k^{\dagger}$, $k^{\ddagger}$, and k_{SCM} were calculated by non-linear regression, minimising the sum of squares between experimental and modelled values of $\ln(C_t/C_0)$ [15]. These parameters were fit to $\ln(C_t/C_0)$ rather than C_t/C_0 to ensure that the model captured the observed kinetics at low values of C_t as well as at high values of C_t.

Uncertainties in the initial rate were calculated from the standard error in the slope of the linear regression in the initial linear region of each kinetic experiment. Uncertainties in the rate constants calculated using the integrated PFO kinetics were calculated using the standard error of the linear regression fit to $\ln(C_t/C_0)$ versus time. Uncertainties in the rate constants calculated by non-linear regression were estimated from the uncertainties in the initial rate.

2. Materials characterisation

2.1. Confirmation of crystal phases using X-ray diffraction (XRD)

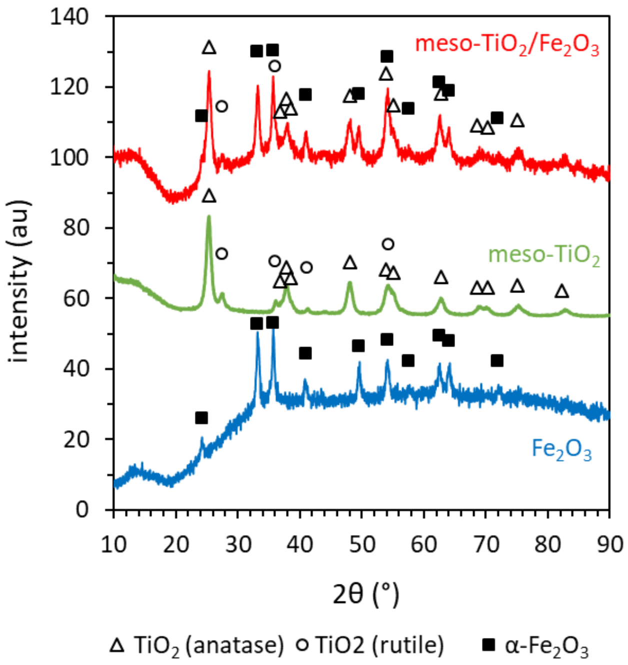

Figure S3: XRD patterns confirm the crystal phases present in meso-TiO_2, meso-TiO_2/Fe_2O_3 and Fe_2O_3 powders. Patterns have been shifted in the y-axis for clarity. The JCPDS reference codes are 01-073-1764 (anatase TiO_2), 01-077-0441 (rutile TiO_2) and 01-079-1741 (hematite).

2.2. Transmission electron microscopy (TEM)

(a) (b)

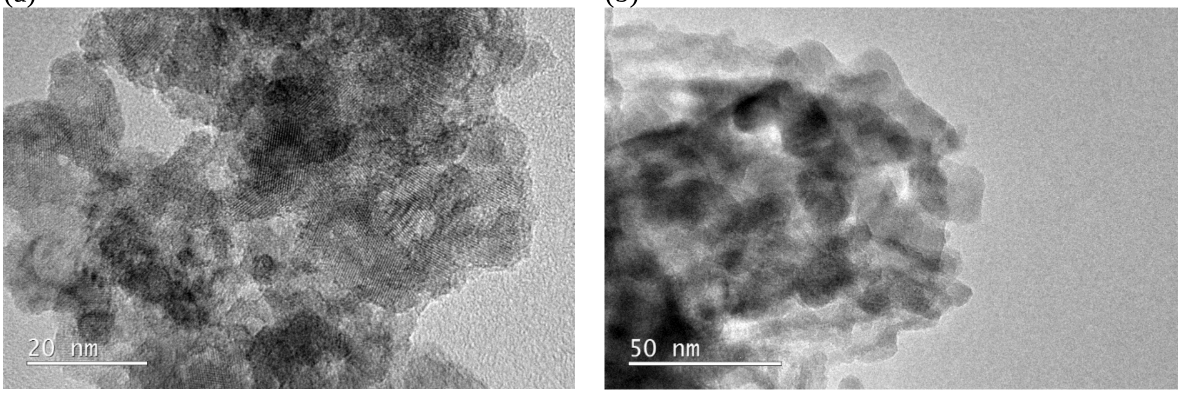

Figure S4: Transmission electron microscopy (TEM) images of (a) meso-TiO_2 and (b) meso-TiO_2/Fe_2O_3 showing the crystallites with a diameter of approximately 10 nm.

2.3. Scanning electron microscopy (SEM)

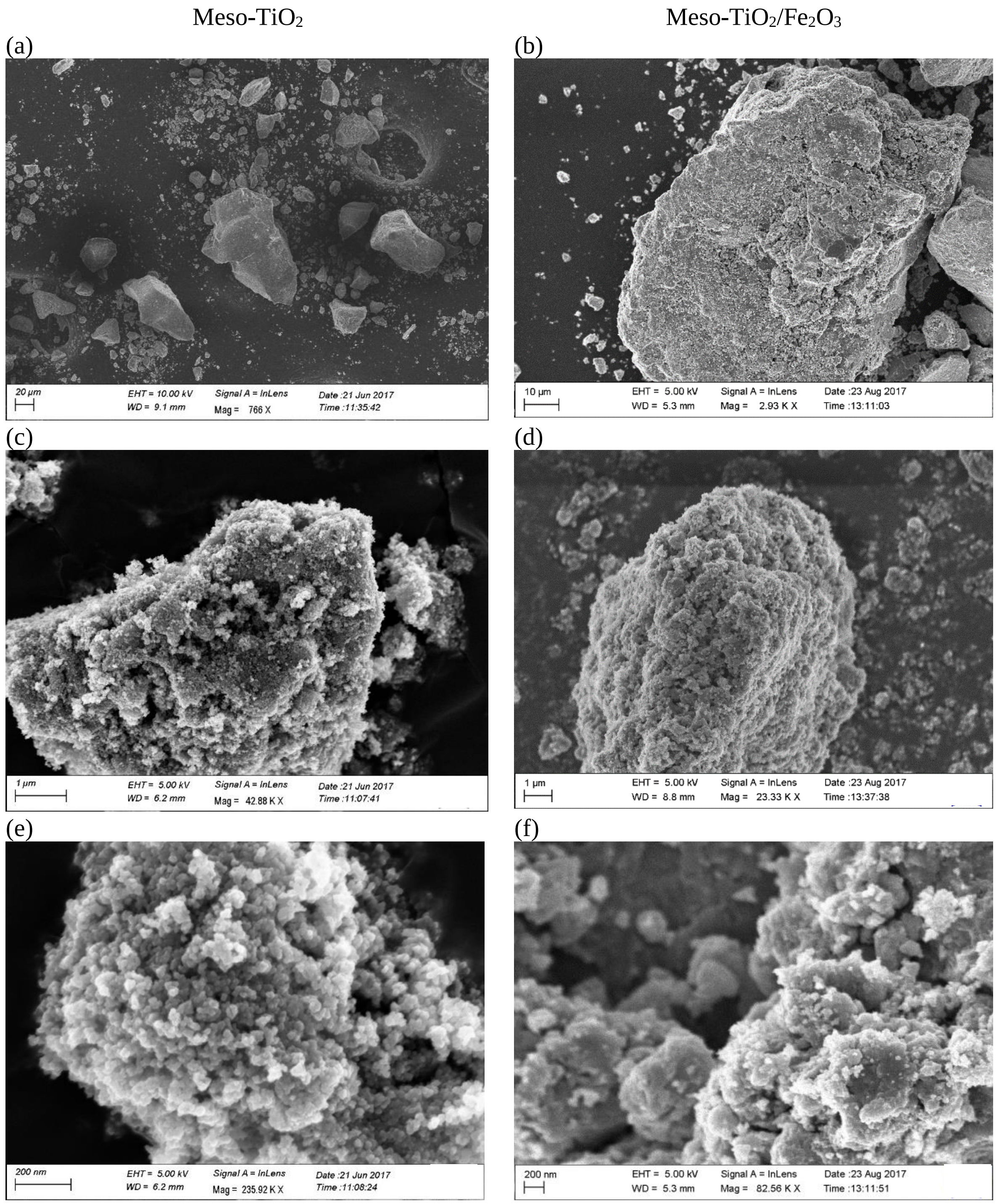

Figure S5: Scanning electron microscopy (SEM) comparison of the morphologies of meso-TiO_2 and meso-TiO_2/Fe_2O_3.

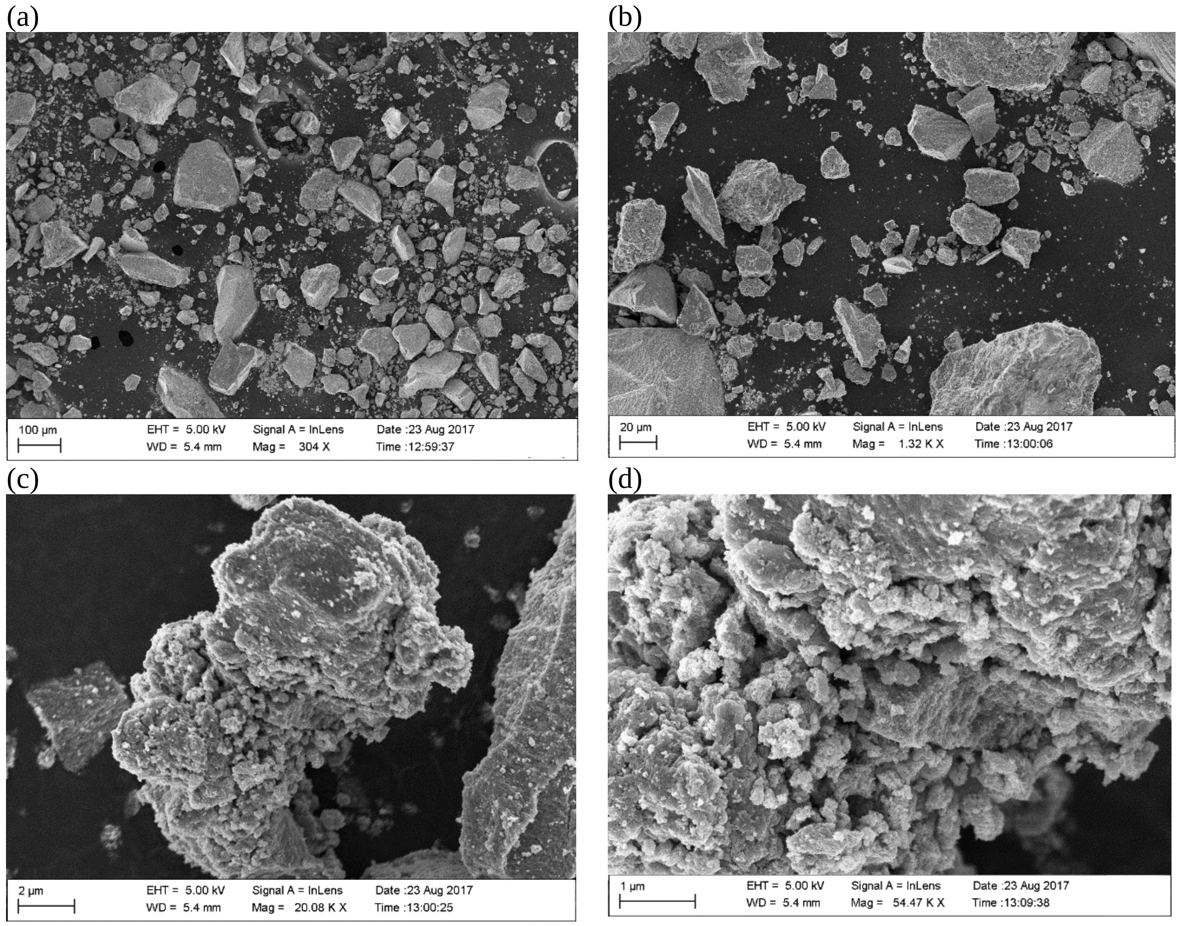

Figure S6: Scanning electron microscopy (SEM) images of the Fe_2O_3 reference sample.

2.4. Quantifying the composition of meso-TiO_2/Fe_2O_3 using X-ray fluorescence (XRF)

The relative abundances of meso-TiO_2 and Fe_2O_3 components within the bulk material of meso-TiO_2/Fe_2O_3 were calculated using the elemental mass percentages returned using X-ray fluorescence (XRF) and the formula:

$$m(a)_{composite} = \frac{I(a)_{composite}}{I(a)_A} \div \left(\frac{I(a)_{composite}}{I(a)_A} + \frac{I(b)_{composite}}{I(b)_B}\right)$$

Equation S19

where *a* is the component of interest (i.e. Fe_2O_3 or meso-TiO_2) and *b* is the second component (*i.e.* meso-TiO_2 or Fe_2O_3), A and B are the reference samples (defined as being composed of 100% component *a* and *b* respectively), m(*a*) is the mass fraction of component *a*, and I(*i*) is the intensity (*i.e.* the mass percentage) of component *i* within the spectrum [14].

Uncertanties were calculated using the formula:

$$\text{error}\ (\%) = \left|100 \cdot \left(\frac{I(a)_{composite}}{I(a)_A} + \frac{I(b)_{composite}}{I(b)_B} - 1\right)\right|$$

Equation S20

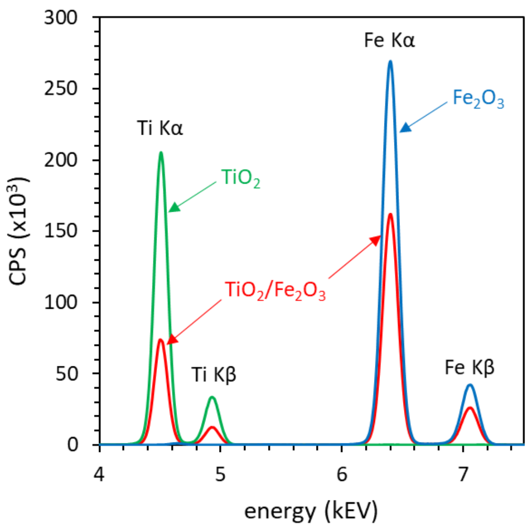

Figure S7: XRF spectra for single-component meso-TiO_2 (green), single-component Fe_2O_3 (blue) reference samples and the meso-TiO_2/Fe_2O_3 composite (red). Spectra have been cropped to the 4-7 keV region to display the Ti and Fe peaks that were used for determining the relative abundance of TiO_2 and Fe_2O_3 within composite meso-TiO_2/Fe_2O_3 using Equation S19.

Table S3: XRF quantification of the bulk composition of meso-TiO_2/Fe_2O_3.

	atomic percent		
	end-member reference samples		meso-TiO_2/Fe_2O_3
Element	meso-TiO_2	Fe_2O_3	
Fe	0.0	97.9	51.5
Ti	77.5	<LoD	32.5
	mass fraction of each component within meso-TiO_2/Fe_2O_3 (%)		
Component	based on a single end-member reference sample	normalised to unity	uncertainty
Fe_2O_3	52.6	55.6	±3.0
TiO_2	41.9	44.4	±2.4

2.5. N_2 adsorption-desorption isotherms for determination of the BET-specific surface area and BJH pore size

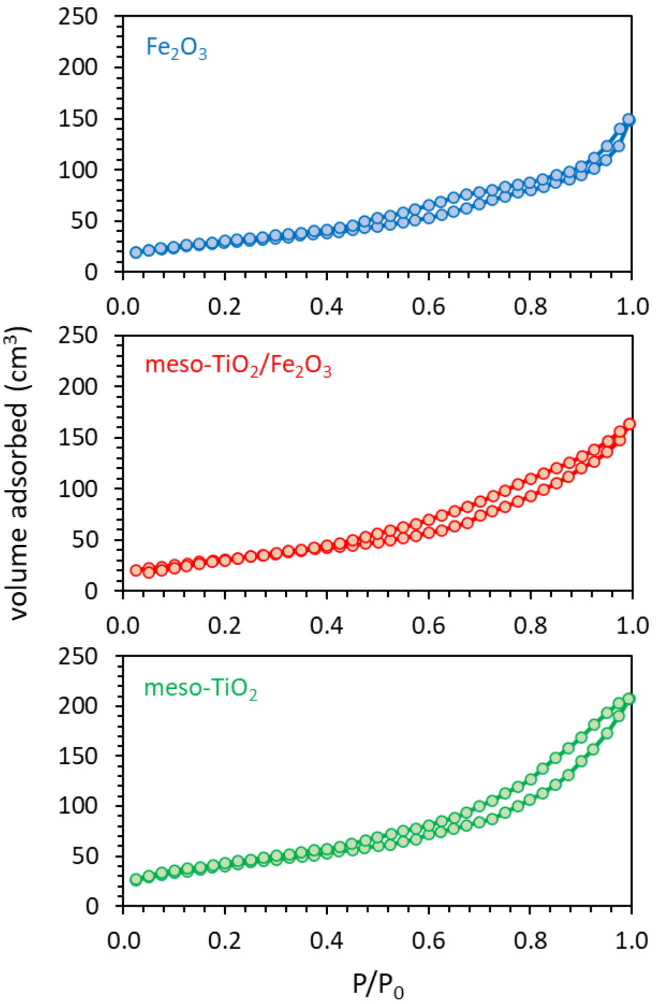

Figure S8: Nitrogen adsorption-desorption isotherms for single-component Fe_2O_3 (blue) and meso-TiO_2 (green) reference samples and the meso-TiO_2/Fe_2O_3 composite photocatalyst (red) collected under standard room temperature and pressure. After outgassing samples and immediately prior to collecting N_2 adsorption-desorption isotherms, material masses were 0.099, 0.0900 and 0.0924 g for Fe_2O_3, meso-TiO_2/Fe_2O_3 and meso-TiO_2 samples respectively. In all cases, the adsorption branch of the hysteresis loop was found at lower y-axis values than the desorption branch.

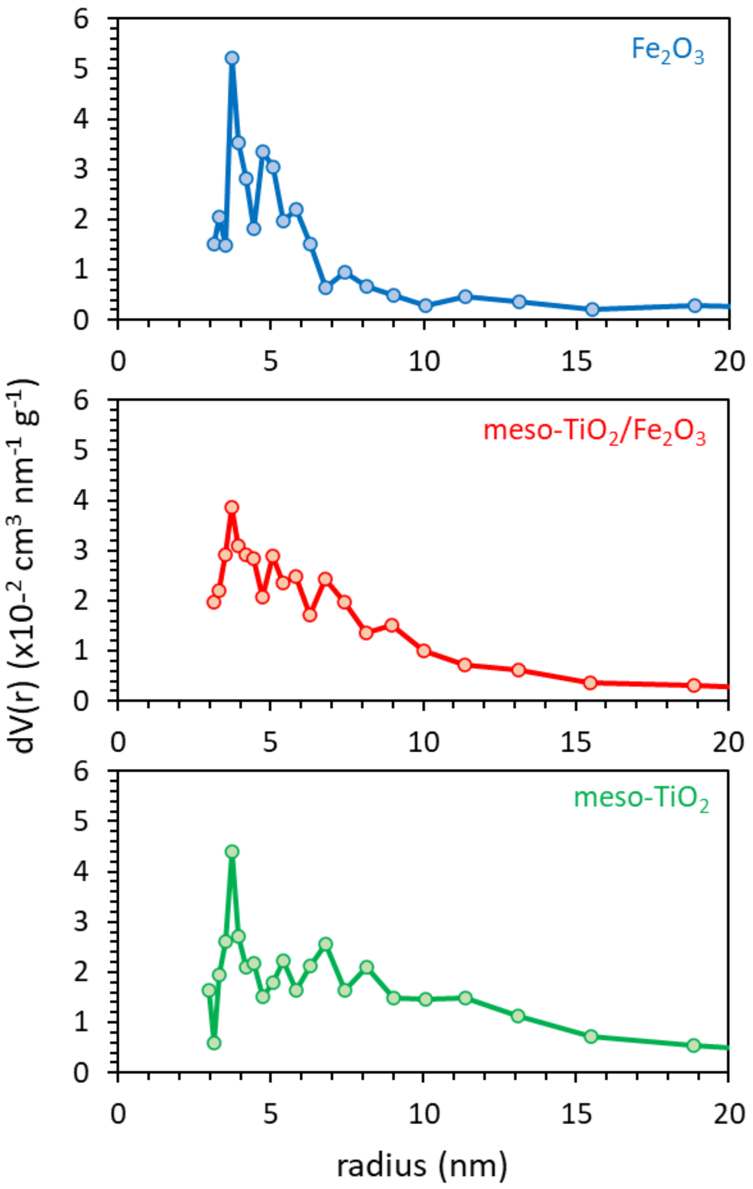

Figure S9: BJH pore size distribution profiles determined from the N_2 adsorption-desorption isotherms in Figure S8.

3. Set-up of photooxidation kinetic experiments

UV-Vis absorbance data was used to identify an appropriate concentration of powdered photocatalyst to use for oxidation kinetic experiments. The total mass balance of arsenic was monitored during a test experiment using a combination of anodic stripping voltammetry and ICP-MS, to verify the experimental set-up. Control experiments were conducted, using aqueous solutions in the dark and under ultraviolet irradiation, and using photocatalyst suspensions in the dark.

3.1. Ultraviolet absorbance by powder suspensions

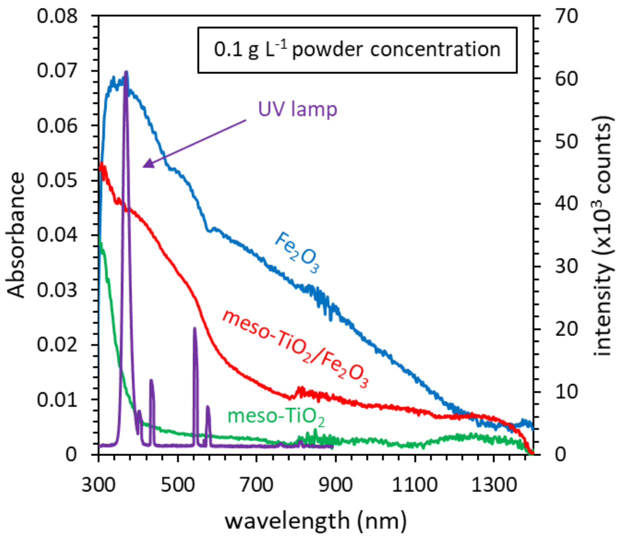

Figure S10: UV-vis absorption spectra of 0.1 g L^{-1} suspensions of each sample (0.1 g L^{-1} was the photocatalyst concentration used in each kinetic experiment) are compared with the spectral output of the 18W ultraviolet lamp used in the kinetic experiments (with the peak centred at λ=368 nm).

In this work, a soft ultraviolet lamp ("UV-A") was used to irradiate photocatalyst suspensions. The spectral output of this lamp was characterised by a major peak centred at λ=368 nm. This lies just within the band edge of meso-TiO_2, and significantly within the band edge of meso-TiO_2/Fe_2O_3 (Figure S10).

Calculations were made using the Beer-Lambert Law to determine an appropriate photocatalyst concentration for subsequent photooxidation kinetic experiments. The wavelength of the ultraviolet lamp was centred at 368 nm. The absorption coefficient, ε, was calculated as a function of powder concentration. This data is presented in (Figure S11a) and shows a slight decrease in ε with increasing powder concentration, due to increased diffuse reflectance of incident photons. This effect was stronger in the meso-TiO_2 containing samples, due to the high refractive index of titania. The extinction coefficient was used to estimate the percentage of incident photons (λ=368 nm) absorbed as a function of both the concentration of powder, and the depth of the suspension (Figure S11b-c). Based on these results, 0.1 g L^{-1} was chosen as the powder concentration, and a 100 mL beaker, giving a depth of 5.6 cm once filled, was chosen as the photoreactor vessel.

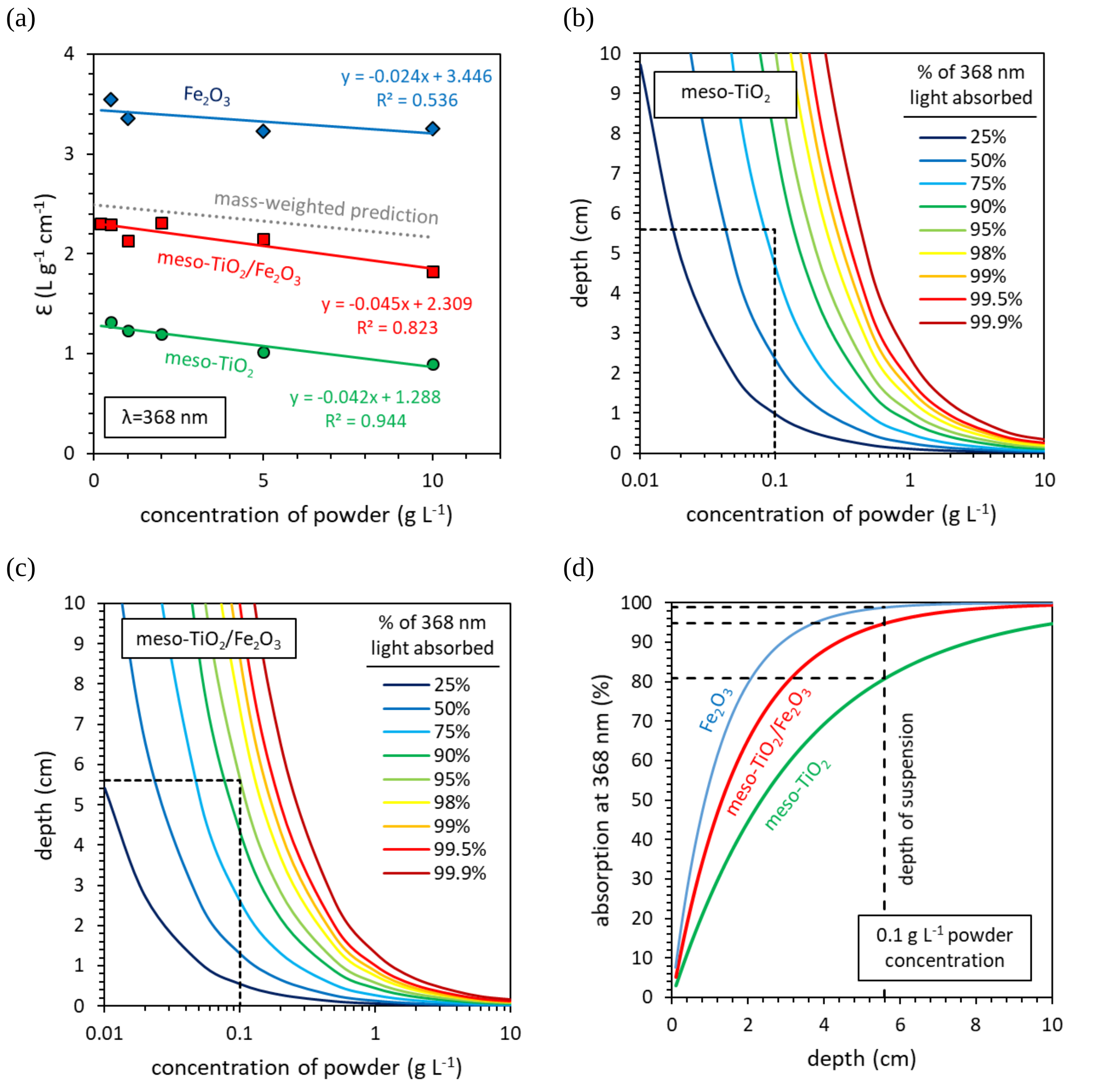

Figure S11: UV-Vis spectroscopy of meso-TiO_2, meso-TiO_2/Fe_2O_3 and Fe_2O_3 used to develop and characterise the photoreactor set-up. (a) Comparison of the extinction coefficient (ε) at different concentrations of suspended solid. The percentage of incident photons (λ=368 nm) absorbed by photocatalyst suspensions as a function of photocatalyst concentration and depth of the reactor vessel is also presented for (b) meso-TiO_2, and (c) meso-TiO_2/Fe_2O_3. A comparison of the percentage of 368 nm radiation absorbed by 0.1 g L^{-1} suspensions of meso-TiO_2, meso-TiO_2/Fe_2O_3, and Fe_2O_3 is presented in (d). Dashed black lines indicate the depth of the suspension used in the kinetic experiments (with a total volume 100 mL). In all cases, dry powder samples were suspended in Milli-Q water without pH adjustment.

In this experimental set-up, the results estimated that meso-TiO_2 suspensions absorb 80.9% of incident photons at 368 nm (Figure S11d). Meso-TiO_2/Fe_2O_3 absorbed 14.8% more photons (94.9% of incident photons). Fe_2O_3 was predicted to absorb 98.8% of incident photons. It was thus anticipated that the majority of incident photons would be absorbed by the suspension in all experiments, and that the supply of photons to meso-TiO_2 and meso-TiO_2/Fe_2O_3 suspensions (i.e. the volumetric rate of photon absorption, VRPA) can be approximated as equivalent in this work The difference between the true VPRA of meso-TiO_2 and meso-TiO_2/Fe_2O_3 is likely smaller than the calculated 14.8%, given that diffuse reflectance from the magnetic stirrer plate beneath the reactor vessel has not been included.

3.2. Operating conditions and constraints

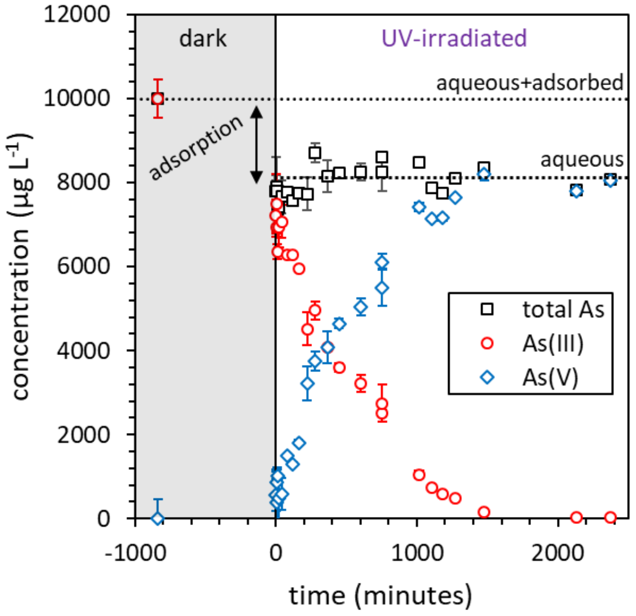

Figure S12: Total mass balance during photocatalytic oxidation. As(III) was determined using anodic stripping voltammetry, and total As was determined by ICP-MS. As(V) was calculated by the subtraction of As(III) from total As. Negative time indicates where the suspension was stirred in the dark to achieve equilibrium adsorption, with t=0 representing when the suspension was first irradiated with the UV lamp. The experimental conditions were 0.1 g L^{-1} meso-TiO_2, pH 7.4 (10 mM HEPES buffer solution), 10 mg L^{-1} initial As(III) concentration, 368 nm wavelength irradiation, and a photon flux of 5.1 mW cm^{-2}, or 107 mW L^{-1}.

Experiments with an initial As(III) concentration of 1 mg L^{-1} indicated that up to 66% of arsenic was removed with just 0.1 g L^{-1} photocatalyst (discussed in the main text). Furthermore, adsorption significantly increased after photooxidation. Consequently, to minimise the interference of adsorption reactions on the determination of photooxidation kinetics, the initial concentration of As(III) was increased to 10 mg L^{-1}. Whilst this is approximately ten times greater than environmentally occurring arsenic concentrations [16] [17], test experiments showed that with 10 mg L^{-1} As(III), less than 20% of the total arsenic is adsorbed (Figure S12). Furthermore, the proportion of total arsenic adsorbed before and after photooxidation is similar (Figure S12) (potentially due to enhanced As(III) adsorption at high concentrations due to multilayer adsorption/surface precipitation reactions). Photocatalytic oxidation was thus better separated from adsorption processes, for the purposes of determining oxidation kinetics. Since the proportion of total arsenic adsorbed showed no significant changes during the course of photooxidation under these conditions, only As(III) was monitored during the subsequent experiments.

3.3. Control experiments

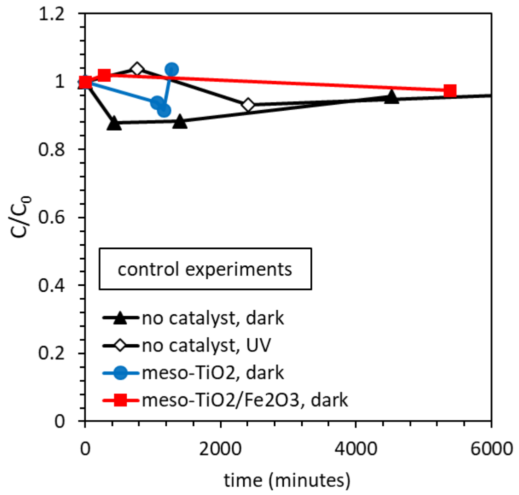

Figure S13: Photooxidation kinetic control experiments. Aqueous solutions, with and without ultraviolet irradiation, and 0.1 g L^{-1} photocatalyst suspended in the dark. The experimental conditions were 10 mg L^{-1} initial As(III), 0 or 0,1 g L^{-1} of photocatalyst, 0.01 M HEPES, pH 7.3±0.1, 368 nm wavelength ultraviolet irradiation, and a photon flux of either 0 or 14.3 mW cm^{-2} (0 or 259 mW L^{-1}).

Photooxidation kinetic control experiments are presented in Figure S13. In the absence of the heterogeneous photocatalyst, no oxidation of As(III) was observed for solutions of 10 mM HEPES, both in the dark and when irradiated with 14.3 mW cm^{-1} ultraviolet radiation (λ=368 nm) (Figure S13). Direct, aqueous phase photooxidation of As(III) was therefore eliminated as a potential interference for photocatalytic oxidation kinetics, within the timescale of 0-4000 minutes. Similarly, no oxidation of As(III) was observed for photocatalyst suspensions in the dark (Figure S13), indicating that no significant oxidation of As(III) occurred during the preparation of each photocatalytic oxidation experiment, where suspensions were stirred in the dark overnight to achieve equilibrium adsorption.

4. The influence of phosphate on the initial rate of As(III) photocatalytic oxidation

Addition of phosphate was used to further probe the mechanism of As(III) photocatalytic oxidation using the two photocatalysts. Phosphate is known to supress arsenic adsorption through competitive adsorption onto mineral surfaces [18] [19], and 10 mg L^{-1} is sufficient to achieve monolayer coverage at pH 7 [20]. By blocking access to the photocatalyst surface, phosphate should supress photocatalytic oxidation of As(III) when adsorbed As(III) is involved in the rate determining step [21].

The addition of 10 mg L^{-1} phosphate decreased adsorption of As(III) onto meso-TiO_2 by 19% (Figure S14a), however since only a small proportion of the initial 10 mg L^{-1} As(III) was adsorbed by 0.1 g L^{-1} meso-TiO_2, the associated uncertainty is large. Surface complexation modelling (SCM) was therefore used to confirm the result, predicting a 5% decrease in [As(III) (ads)]. The addition of phosphate affected the adsorption of As(III) onto meso-TiO_2/Fe_2O_3 much more strongly, with decreases of 62% and 36% calculated using experimental and SCM data respectively.

The initial rate of As(III) photooxidation in the presence of meso-TiO_2/Fe_2O_3 decreased by 43% when 10 mg L^{-1} phosphate was added (Figure S14b). This is similar in magnitude to the 62% decrease in As(III) adsorption, and suggests that the rate of reaction is controlled by the concentration of As(III) adsorbed onto the photocatalyst surface, in agreement with the main text.

The opposite effect was seen for meso-TiO_2: the initial rate of As(III) photooxidation increased by 56% upon addition of 10 mg L^{-1} phosphate, despite the 22% decrease in adsorbed As(III). This suggests that the rate of photocatalytic oxidation is not controlled by the concentration of adsorbed As(III) under these experimental conditions, in agreement with the zero-order kinetics identified in the main text. It is reported that under certain conditions phosphate can enhance photocatalysis, either by the formation of hydrogen-bonded complexes between adsorbed phosphate and H_2O facilitating charge transfer and the subsequent generation of intermediate reactive oxygen species (ROS), such as •OH and H_2O_2, or by the attraction between adsorbed phosphate (which is negatively charged at pH 7) and valence band holes, improving charge extraction [21].

TAS measurements showed that As(III) scavenges charge carriers from meso-TiO_2, increasing transient absorption lifetimes. The half-lives of transient absorption did not decrease after addition of phosphate; As(III) scavenged charge carriers just as effectively (Figure S14c). Consequently, TAS provides further evidence that site-blocking does not supress the photocatalytic oxidation of As(III) using meso-TiO_2, suggesting that the rate law depends on charge extraction and the generation of ROS intermediates rather than the concentration of adsorbed As(III).

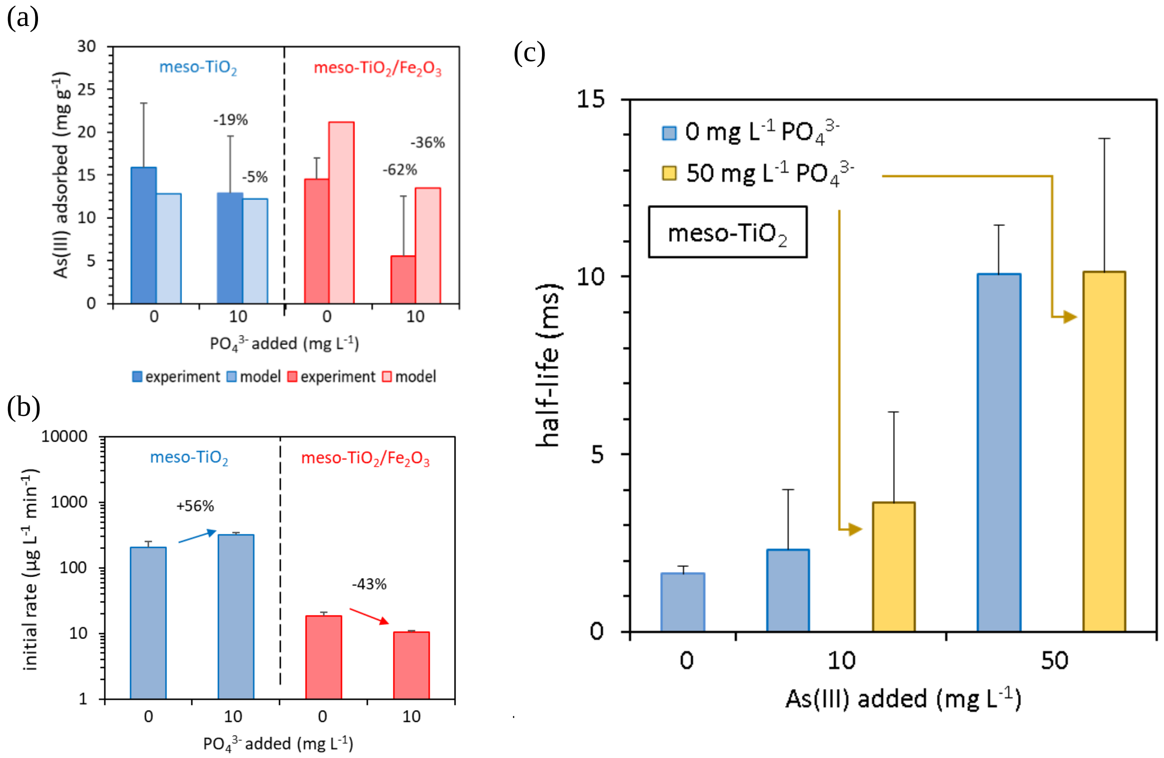

Figure S14: The influence of phosphate (10 mg L^{-1}) on (a) the adsorption of As(III), and (b) initial rates in the photocatalytic oxidation of As(III). The experimental conditions were 10 mg L^{-1} As(III), 0.1 g L^{-1} photocatalyst, 10 mM HEPES (pH 7.3±0.1), 14 mW cm^{-2} light intensity (λ=368 nm), and 100 mL total volume. (c) The influence of phosphate (10 mg L^{-1}) on the transient absorption half-lives of 10 and 50 mg L^{-1} As(III) in the presence of meso-TiO_2 (1 g L^{-1}) in 10 mM HEPES (pH 7.3±0.1). The results presented are averages of measurements taken at λ=600, 700, 800, 900 and 1000 nm and error bars indicate the standard deviation.

5. Langmuir-Hinshelwood kinetics

5.1. Adsorption isotherm fitting

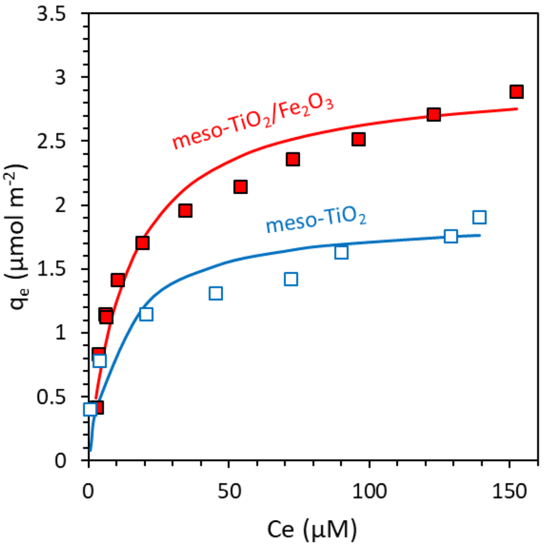

Figure S15: A sub-selection of the adsorption isotherms reported previously [14] *were fit in the C_e range of 0-11 mg L^{-1} (0-150 µM) range to obtain Langmuir adsorption isotherm parameters for the region before significant multilayer adsorption had occurred. C_e denotes the concentration of aqueous As(III) at equilibrium, whilst q_e denotes the concentration of adsorbed As(III) at equilibrium. Experimental conditions were 1 g L^{-1} photocatalyst, pH 7.0±0.1 and 0.01 M NaCl. The adsorption isotherm parameters obtained were K_L = 1.18 ±0.55 L mg^{-1} and Q_{max} = 15.7 ±1 mg g^{-1} for meso-TiO_2 and K_L = 0.93 ±0.14 L mg $^{-1}$ and Q_{max} = 22.8 ±0.7 mg g^{-1} for meso-TiO_2/Fe_2O_3.*

5.2. Linearised Langmuir-Hinshelwood kinetics and initial rate analysis

Analysis of initial rates using Langmuir-Hinshelwood kinetics was achieved using the following two linearised equations:

$$\frac{1}{\text{rate}} = \frac{1}{K_L k} \cdot \frac{1}{C} + \frac{1}{k}$$

Equation S21

$$\frac{C}{\text{rate}} = \frac{1}{k} \cdot C + \frac{1}{K_L k}$$

Equation S22

where the reaction rate is the measured decrease in [As(III) (aq)] with time, K_L is the Langmuir parameter, reflecting the binding affinity between the substrate and the catalyst surface (L mg^{-1}), and k is the rate constant (mg L^{-1} min^{-1}) [13]. These two equations were labelled as 'linearisation A' and 'linearisation B' respectively. Values of the Langmuir-Hinshelwood rate constant, k_{LH} (min^{-1}), reported in this work were thus obtained by taking out the Q_{max} term, using the equation:

$$k_{LH} = \frac{k}{Q_{max} C_s}$$

Equation S23

where C_s is the concentration of suspended photocatalyst (0.1 g L^{-1}).

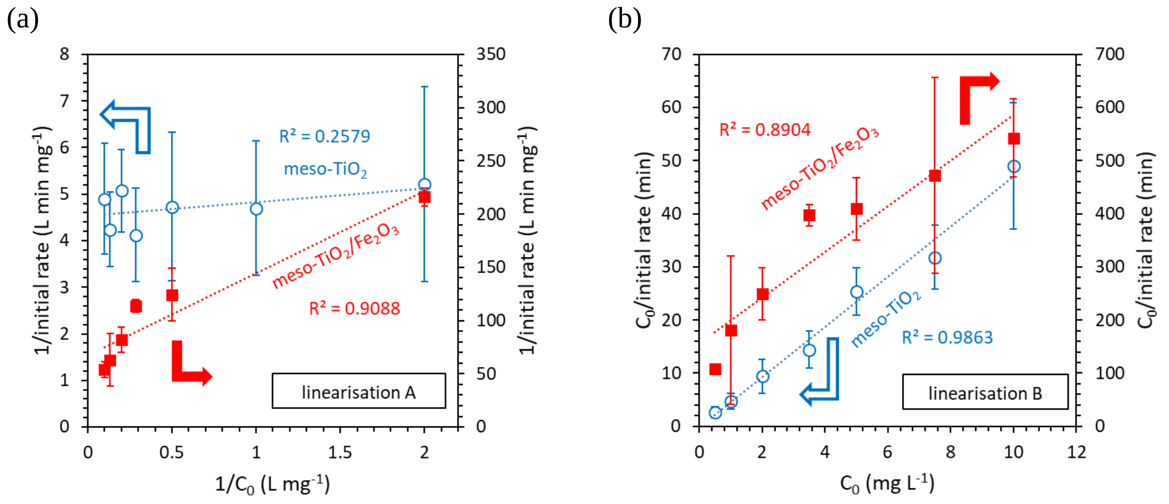

Figure S16: The relationship between initial As(III) concentration and the initial rate of As(III) oxidation kinetics, plotted in two linearised forms of the Langmuir-Hinshelwood kinetic model [13].

The analysis of initial rates using linearisation of the Langmuir-Hinshelwood rate equation yielded physically insignificant results for meso-TiO_2, owing to the zero-order dependence previously established in the main text. Under the 'linearisation A' approach, the coefficient of determination (R^2) in the linear regression was only 0.2579 (Figure S16a). In the 'linearisation B' approach, a negative y-intercept, yielding a physically meaningless negative value of K_L was achieved, unless the data point at C_0 was excluded to alter the slope of the linear regression (Figure S16b). Langmuir adsorption constants, K_L, obtained using the two linearisation approaches were much larger than the value obtained from experimental adsorption isotherms (Figure S15, 15.2±12.0 and 7.15±21 versus 1.18±0.55 L mg^{-1}), to approximate the zero-order relationship. The large difference between K_L values indicates the failure of the Langmuir-Hinshelwood model to describe photocatalytic oxidation using meso-TiO_2 [13].

More physically meaningful results were achieved for meso-TiO_2/Fe_2O_3. The k_L value obtained using 'linearisation A' agreed well with the value obtained from adsorption isotherms (0.843±0.2 versus 0.93±0.14 L mg^{-1}), although K_L was only 0.274±0.089 when calculated using 'linearisation B'. However, the quality of the linear regression was poor ($R^2 \approx 0.90$), owing to a change in steepness at C_0=3.5 mg L^{-1}. The effect was significant, for instance using 'linearisation B', the slope obtained is four times steeper using data in the range C_0=0-3.5 mg L^{-1}, than if using data in the range $C_0 \geq 3.5$ mg L^{-1} (Figure S16b).

5.3. Non-linear Langmuir-Hinshelwood kinetics

(a) (b)

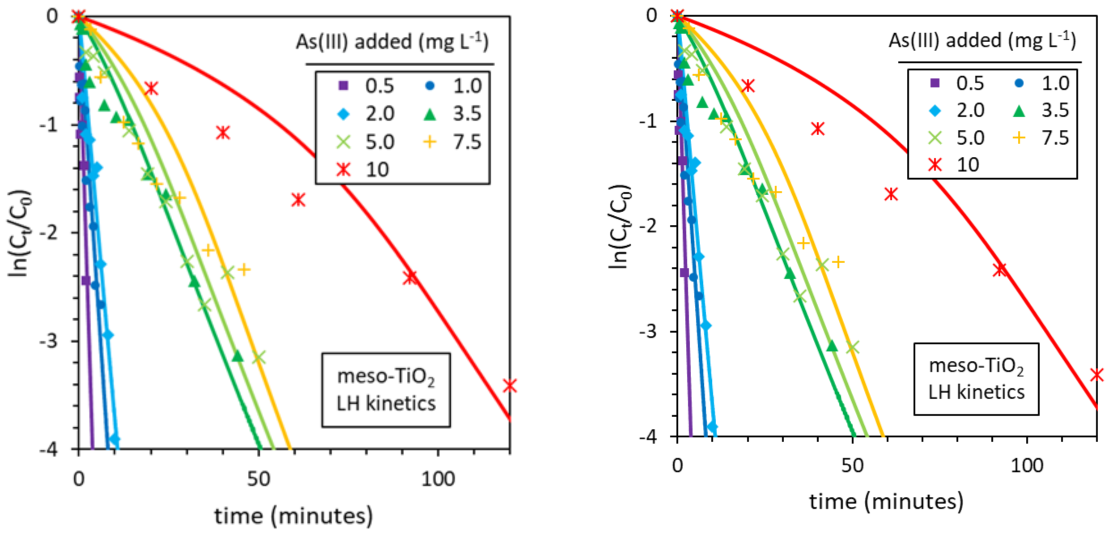

Figure S17: The Langmuir-Hinshelwood kinetic model fit to experimental data at later times. The Langmuir adsorption constant, K_L, was calculated from experimental adsorption isotherms previously reported [14]*, using only the data in the range of C_e = 0-10 mg L^{-1} to cover the experimental range in this work, and reduce the error in K_L due to multilayer sorption (Figure S15).*

5.4. Langmuir adsorption isotherm prediction of As(III) distribution

Whilst increased partitioning of total As(III) to the adsorbed phase is predicted by the Langmuir adsorption isotherm (agreeing with the SCM), the transition from low to high adsorption partitioning is relatively gentle, and with no significant feature appearing around the 78% mark which corresponds to where curvature was observed in the experimental data at $\ln(C/C_0)$=-1.5 (Figure S18). The effect of oxidation progress on adsorption partitioning is similar across all values of C_0 above 2 mg L^{-1}. There is little significant difference between calculated results for meso-TiO_2 and meso-TiO_2/Fe_2O_3.

(a) (b)

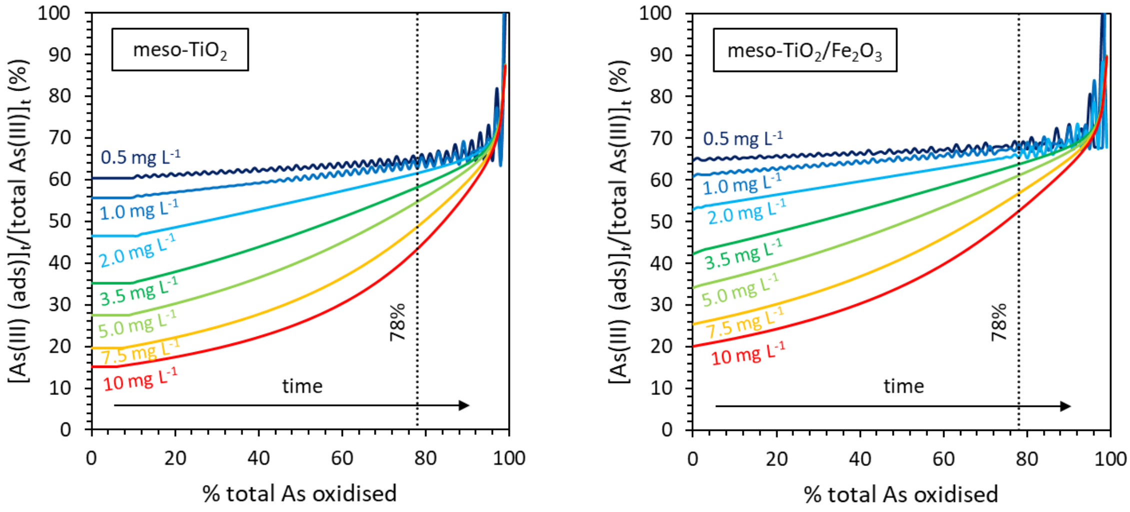

Figure S18: The percentage of total As(III) present in the adsorbed phase as a function of increasing photooxidation. Results were obtained using the Langmuir adsorption isotherm model with the previously determined K_L and Q_{max} parameters.

6. As(V) deactivation kinetics: a simple model using C_0 and C_t parameters only

If we construct a rate law where the rate depends on the concentration of As(V) present, rather than the concentration of As(III), it is evident that initial rate analysis will show zero-order kinetics, but data at later times will not. This is because at t=0, there is no As(V), and the concentration of As(V) continually increases through each experiment. If our rate is supressed by the presence of As(V) then the shape of the pseudo-first order kinetic model obtained from the experimental data can be emulated.

For instance, we can consider a scenario where the absolute rate decreases linearly with increasing concentrations of As(V). The reaction would cease once all arsenic is present as As(V). Since the fraction of total As converted to As(V) at time *t* is equal to $1-\frac{C_t}{C_0}$, we arrive at the following rate equation:

$$\frac{\mathrm{dC}}{\mathrm{dt}} = \mathrm{k}^{\dagger}\left(1-\left(1-\frac{\mathrm{C_t}}{\mathrm{C_0}}\right)\right)$$

Equation S24

where C is the concentration of aqueous As(III), $k^{\dagger}$ is the rate constant (μg L^{-1} min^{-1}), and C_t and C_0 are the concentrations of aqueous As(III) at time *t* and t=0 respectively. This equation reduces to:

$$\frac{\mathrm{dC}}{\mathrm{dt}} = \mathrm{k}^{\dagger}\left(\frac{\mathrm{C_t}}{\mathrm{C_0}}\right)$$

Equation S25

and consequently, relates to the original pseudo-first order rate equation through the expression:

$$\mathrm{k}^{\dagger} = \mathrm{kC_0}$$

Equation S26

This simple rate law therefore satisfies both conditions needed for the rate law: (1) the rate constant is independent of the concentration of As(III), since the term $\frac{C_t}{C_0}$ in Equation S25 is always 1 at t=0, regardless of the concentration of As(III) added, and (2) the shape of pseudo-first order kinetics will be observed in the serial data, since the rate is proportional to C_t.

Application of this rate law to the experimental data is presented in Figure S19. The rate law successfully reconstructed the observed pseudo-first order behaviour, with reaction rates decreasing as time increases. The influence of C_0 upon the rate constant was a lot weaker than in the PFO model (discussed in the main text). Firstly, the trend for an exponential decrease in the rate constant with increasing C_0 is a lot weaker (R^2=0.4348 versus R^2=0.9684 in the PFO model). Secondly and more importantly, the slope of log($k^{\dagger}$) versus C_0 (-0.197±0.101) is significantly shallower than the slope of log(k_1) versus C_0 (-1.17±0.09). This suggests that photocatalyst deactivation due to presence of As(V) is a probable cause for the apparent first-order kinetics observed in the data at later times.

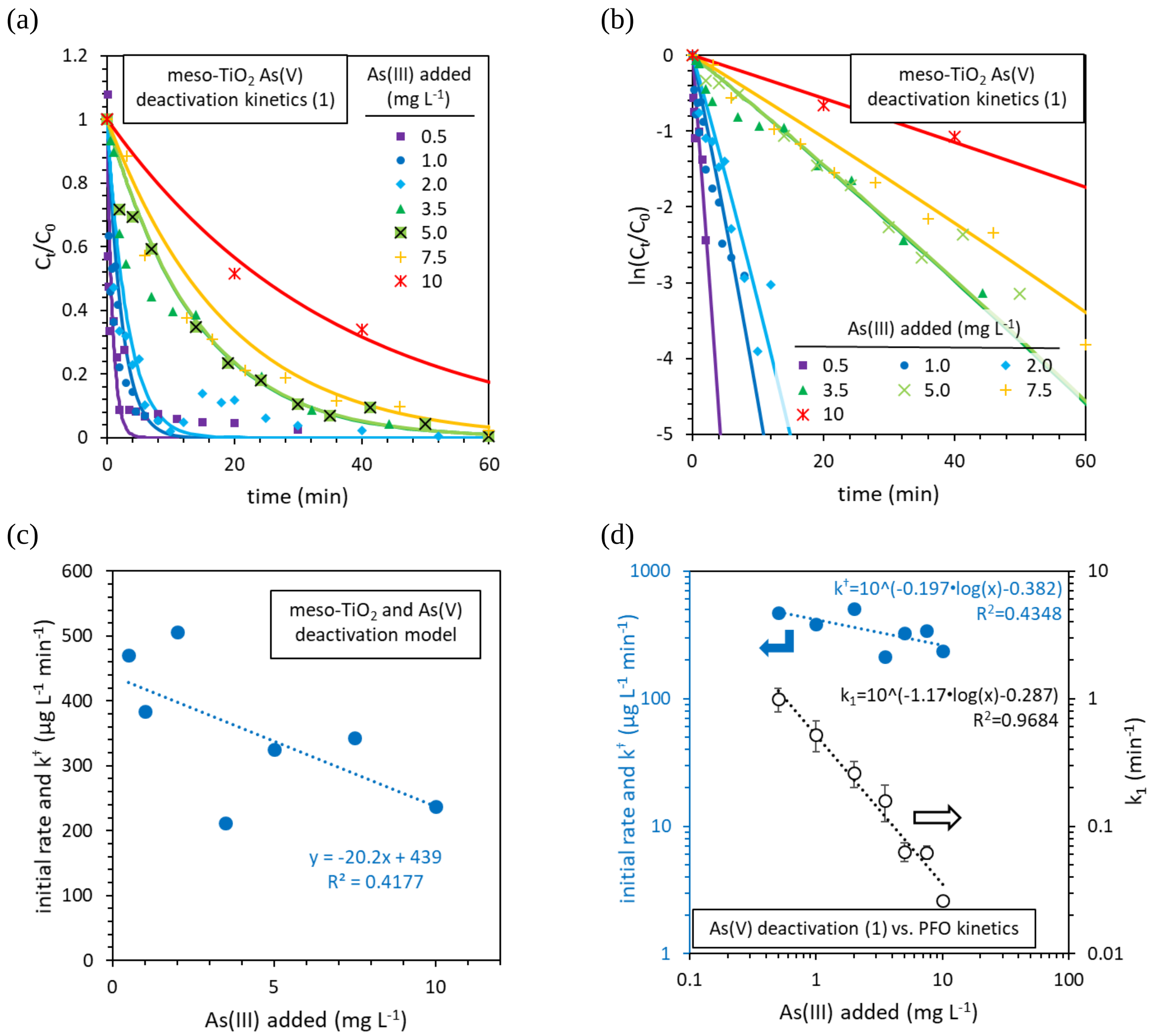

Figure S19: Application of the rate law given in Equation S24 to experimental data using the single-component meso-TiO_2 photocatalyst. Rate constant $k^\dagger$ was obtained by minimising the sum of squares between the model and experimental observations of $\ln(C/C_0)$. The goodness of fit was calculated with R^2 = 0.8991, 0.7964, 0.9155, 0.9641, 0.9513, 0.9708, 0.9957 for the 0.5, 1, 2, 3.5, 5, 7.5 and 10 mg L^{-1} initial As(III) data sets.

7. Surface complexation modelling (SCM)

7.1. SCM parameters

Table S4: All parameters used for surface complexation modelling in Visual MINTEQ. Surface charge parameters and arsenic surface complexation constants were taken from our previous work using FITEQL and re-optimised for Visual MINTEQ by re-fitting potentiometric titrations and adsorption edges [14] *and phosphate surface complexation constants were calculated by fitting pH adsorption edges reported elsewhere* [22] [23]. *Log(K^0) values are reported for each surface complexation reaction, along with the change in the surface potential. Meso-TiO_2 and meso-TiO_2/Fe_2O_3 suspensions were modelled in 0.01 M NaCl, at pH 7, with total arsenic was varied between 0.5 and 10 mg L^{-1}, as per the experimental work. The distribution of As(III) and As(V) was varied between 100% As(III) and 100% As(V) in 1% increments.*

Property/reaction	Value		$\Delta\psi$
pH	7		-
Ionic strength (M)	0.01		-
NaCl (M)	0.01		-
	Fe_2O_3	Meso-TiO_2	
Surface area (m^2 g^{-1})	103.4	110.1	-
Concentration of solid (g L^{-1})	0.068	0.032	-
Site density (sites nm^{-2})	4.0	3.0	-
Concentration of sites (μM)	46.7	17.5	-
Inner capacitance, C_1 (F m^{-2})	0.8	1.3	-
Outer capacitance, C_2 (F m^{-2})	0.2	0.2	-
$>SOH + H^+ = >SOH_2^+$	5.48	1.50	$\psi_0 - \psi_\beta$
$>SOH = >SO^- + H^+$	-12.3	-8.10	$-\psi_0+\psi_\beta$
$>SOH + H^+ + Cl^- = >SOH_2^+$---$Cl^-$	8.98	4.63	$\psi_0-\psi_\beta$
$>SOH + Na^+ = >SO^-$---$Na^+ + H^+$	-9.3	-5.5	$-\psi_0+\psi_\beta$
$2 >SOH + H_3AsO_3 = (>SO)_2AsOH + 2H_2O$	5.3	-	0
$2 >SOH + H_3AsO_3 = (>SO)_2AsO^- + H^+ + 2H_2O$	-0.5	3.0	$-\psi_\beta$
$>SOH + H_3AsO_3 = >SOH_2^+$---$AsO(OH)_2^-$	5.7	4.0	$\psi_0-\psi_\beta$
$2 >SOH + AsO_4^{3-} + 3H^+ = (>SO)_2AsO_2H + 2H_2O$	29.6	-	0
$2 >SOH + AsO_4^{3-} +2H^+ = (>SO)_2AsO_2^- + 2H_2O$	27.8	28.7	$-\psi_\beta$
$>SOH + AsO_4^{3-} +H^+ = >SOAsO_3^{2-}$	24.0	26.6	$-2\psi_\beta$
$2 >SOH + PO_4^{3-} + 3H^+ = (>SO)_2PO_2H + 2H_2O$	29.7	26.7	0
$2 >SOH + PO_4^{3-} +2H^+ = (>SO)_2PO_2^- + 2H_2O$	28.7	28.9	$-\psi_\beta$
$>SOH + PO_4^{3-} +H^+ = >SOPO_3^{2-}$	22.9	22.7	$-2\psi_\beta$

7.2. SCM-constrained kinetics versus pseudo-first order (PFO) and Langmuir-Hinshelwood (LH) kinetics

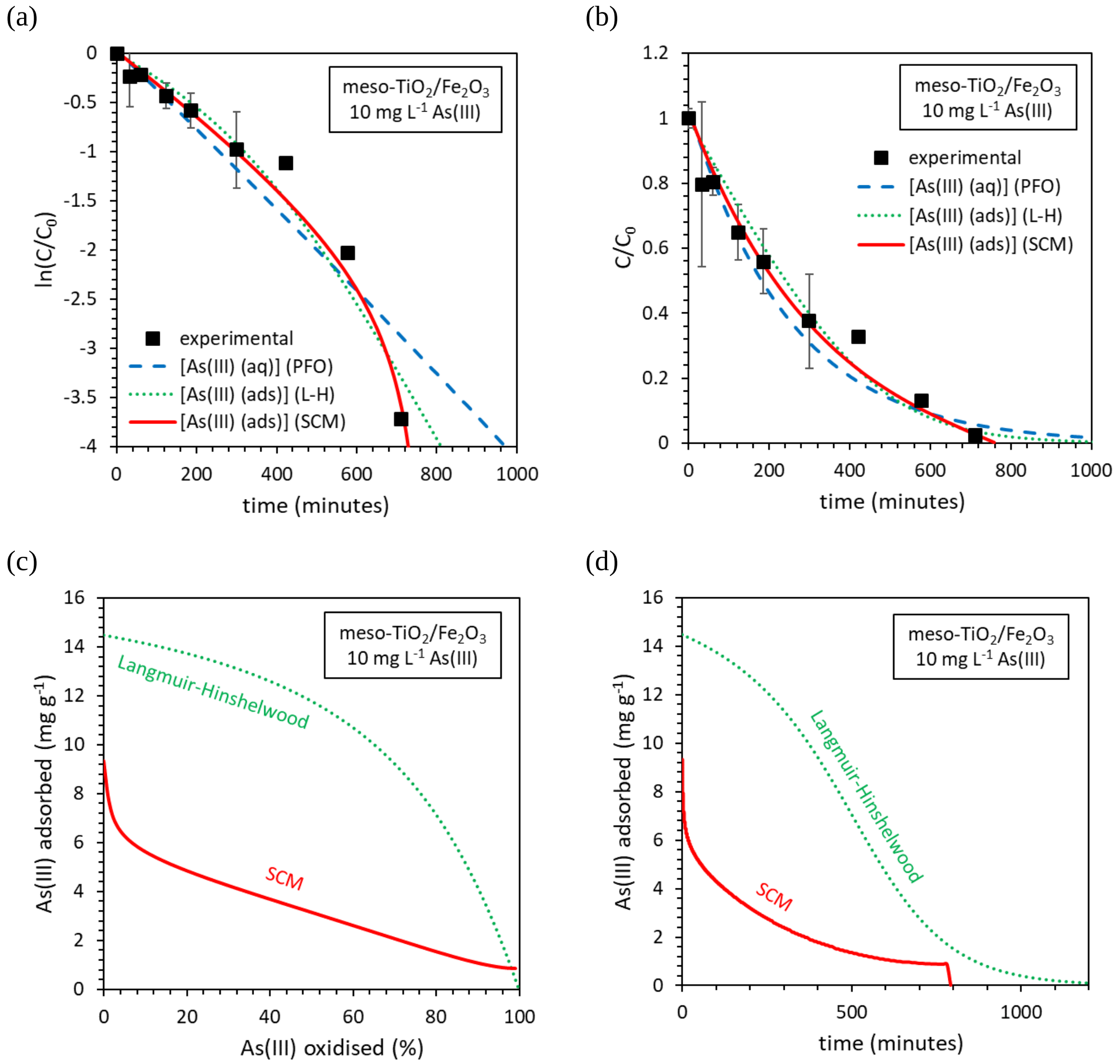

Figure S20: Comparison of meso-TiO_2/Fe_2O_3 SCM-constrained kinetics with pseudo-first order (PFO) and Langmuir-Hinshelwood (LH) kinetics.

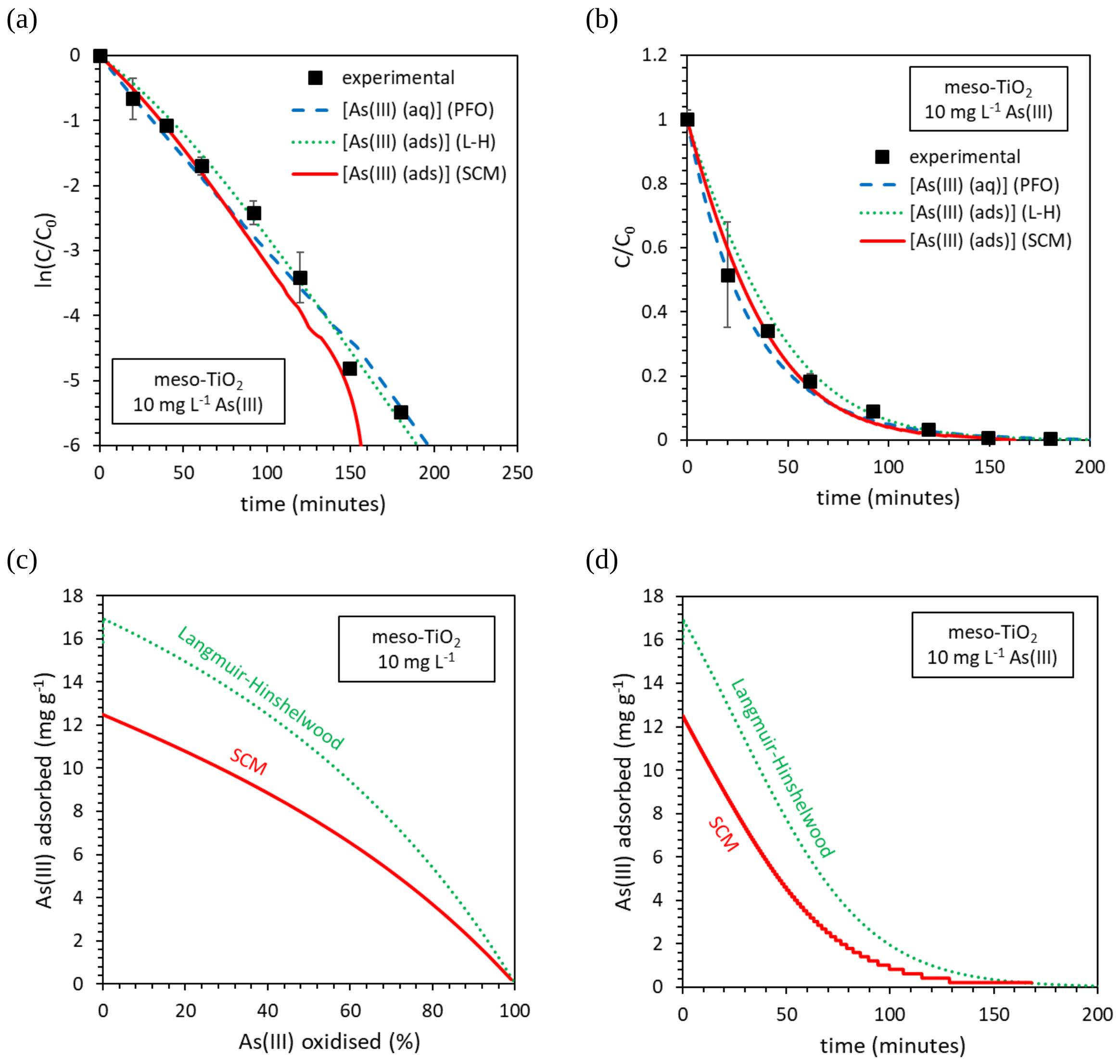

Figure S21: Comparison of meso-TiO_2 SCM-constrained kinetics with pseudo-first order (PFO) and Langmuir-Hinshelwood (LH) kinetics.

8. Transient absorption spectroscopy (TAS)

8.1. Component additivity of optical density

The optical density of meso-TiO_2/Fe_2O_3 in the absence of photoexcitation was predicted for all wavelengths above 600 nm using a linear combination of meso-TiO_2 and Fe_2O_3 reflectance data, weighted according to their mass fraction (calculated using XRF in section 0), indicating component additivity.

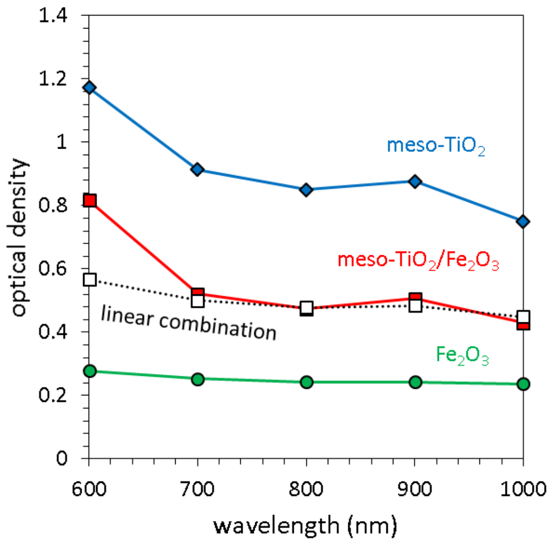

Figure S22: The component additivity of optical density, calculated from the diffuse reflectance of dry powders.

8.2. Transient absorption of dry powders

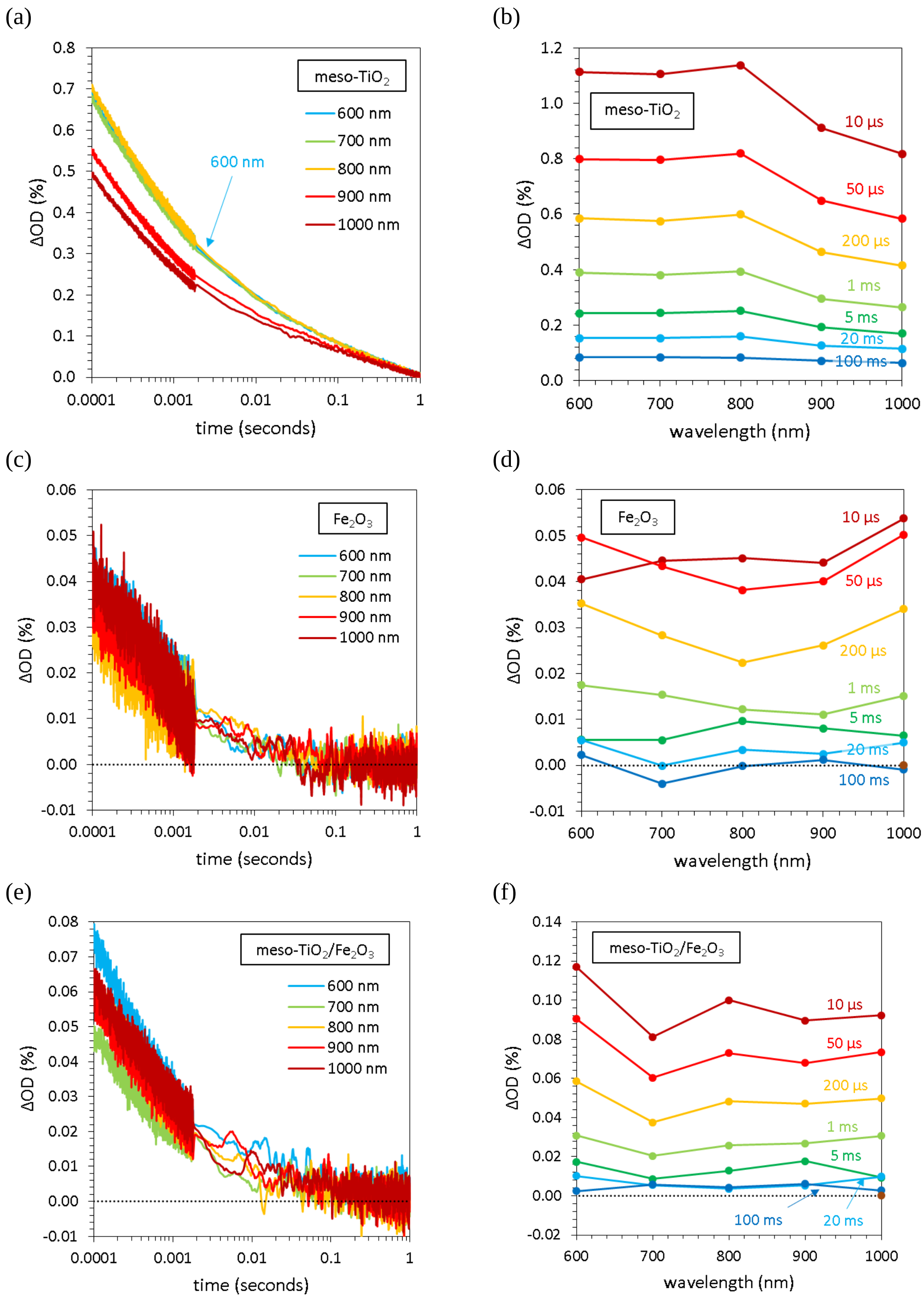

Figure S23: Transient absorption spectroscopy of dry powders, with decay kinetics on the left and spectra on the right. (a-b) meso-TiO_2, (c-d) meso-TiO_2/Fe_2O_3, and (e-f) Fe_2O_3. The operating conditions were λ_{exc} = 355 nm, ~1.0 mJ cm^{-2} pulse^{-1}, 6 ns pulse width, laser repetition rate ~1 Hz.

Table S5: Power law decay parameters obtained by fitting normalised data for dry powders using Origin. The half-life was calculated by rearranging the power law decay function into the form $t = \left(\frac{0.5}{A}\right)^{-\frac{1}{\alpha}}$*. Data was normalised to give ΔOD=1 at t=0.1 ms.*

sample	wavelength (nm)	A	A (normalised)	α	R^2	half-life (ms)
meso-TiO_2	600	0.059 ±0.001	0.0834 ±0.0007	0.27 ±0.002	0.994	1.310
	700	0.0567 ±0.0008	0.0812 ±0.0006	0.273 ±0.002	0.996	1.270
	800	0.0573 ±0.0009	0.0787 ±0.0006	0.276 ±0.002	0.996	1.230
	900	0.0409 ±0.0004	0.0727 ±0.0003	0.285 ±0.001	0.998	1.140
	1000	0.0365 ±0.0004	0.0724 ±0.0003	0.285 ±0.001	0.998	1.140
meso-TiO_2/Fe_2O_3	600	0.0017 ±0.0001	0.0218 ±0.0004	0.416 ±0.008	0.969	0.530
	700	0.00127 ±0.00009	0.0261 ±0.0006	0.396 ±0.009	0.964	0.576
	800	0.00143 ±0.00009	0.0226 ±0.0004	0.412 ±0.008	0.974	0.539
	900	0.0017 ±0.0001	0.0282 ±0.0006	0.388 ±0.009	0.958	0.598
	1000	0.002 ±0.0001	0.0301 ±0.0005	0.38 ±0.007	0.975	0.618
Fe_2O_3	600	0.00045 ±0.00007	0.0101 ±0.0004	0.5 ±0.02	0.907	0.401
	700	0.00057 ±0.00008	0.0145 ±0.0005	0.46 ±0.02	0.919	0.452
	800	0.00049 ±0.0001	0.0164 ±0.0009	0.45 ±0.02	0.804	0.472
	900	0.00032 ±0.00006	0.0088 ±0.0004	0.51 ±0.02	0.882	0.385
	1000	0.059 ±0.001	0.0834 ±0.0007	0.27 ±0.002	0.994	1.310

8.3. The influence of phosphate on transient absorption

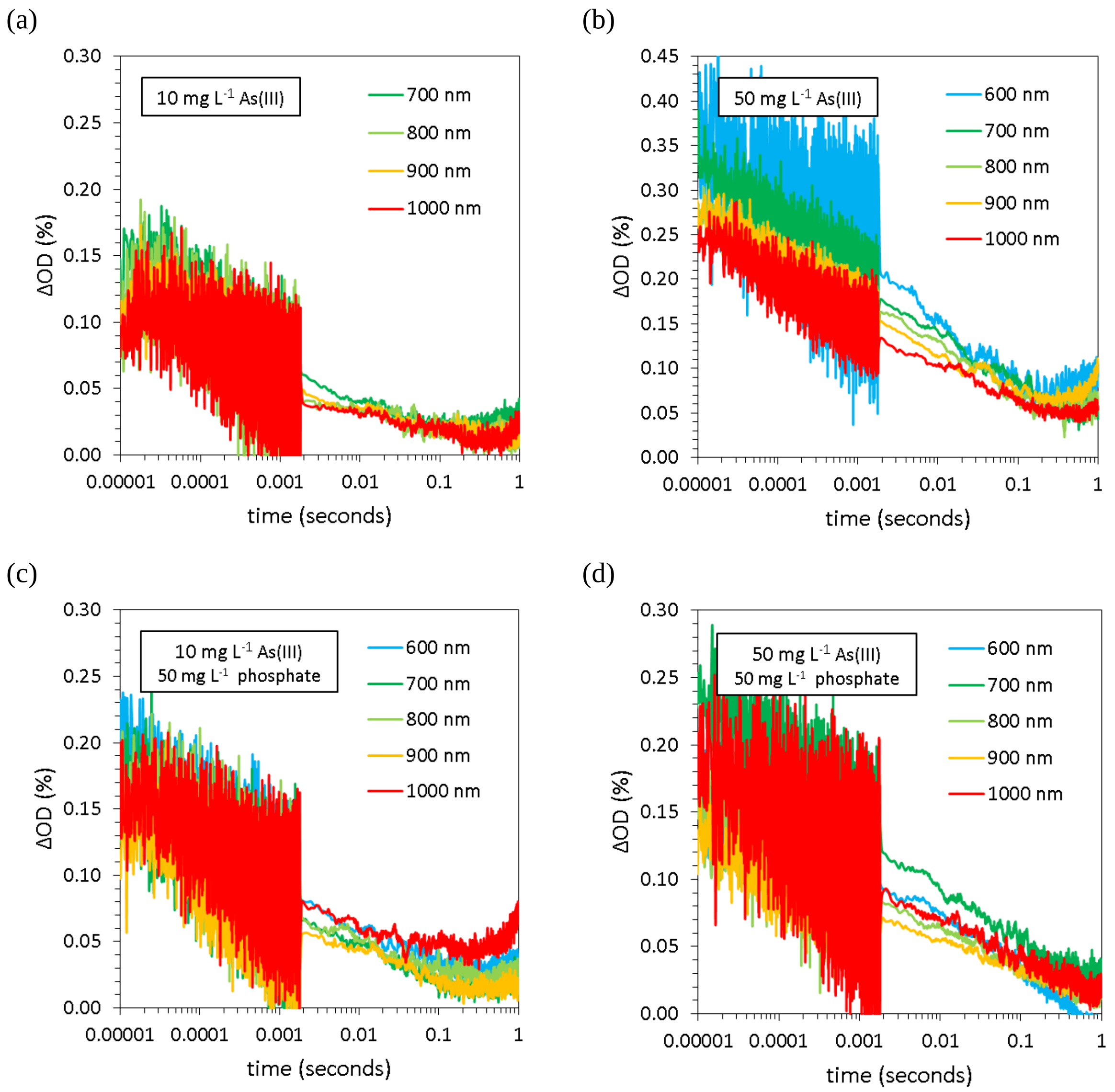

Figure S24: The influence of phosphate on the transient absorption of meso-TiO_2 suspensions. Meso-TiO_2 (1 g L^{-1}) was suspended in 10 mM HEPES (pH 7.3±0.1), containing (top) 0 or (bottom) 50 mg L^{-1} phosphate, and (left) 10 or (right) 50 mg L^{-1} As(III). (a) 10 mg L^{-1} As(III) and 0 mg L^{-1} phosphate, (b) 50 mg L^{-1} As(III) and 0 mg L^{-1} phosphate, (c) 10 mg L^{-1} As(III) and 50 mg L^{-1} phosphate, (d) 50 mg L^{-1} As(III) and 50 mg L^{-1} phosphate. The operating conditions were λ_{exc} = 355 nm, ~1.0 mJ cm^{-2} $pulse^{-1}$, 6 ns pulse width, laser repetition rate ~1 Hz.

9. References

[1] J.C. Bullen, A. Torres-huerta, P. Salaün, J.S. Watson, S. Majumdar, R. Vilar, D.J. Weiss, Portable and rapid arsenic speciation in synthetic and natural waters by an As(V)-selective chemisorbent, validated against anodic stripping voltammetry, Water Res. 175 (2020) 115650. https://doi.org/10.1016/j.watres.2020.115650.

[2] P. Atkins, J. De Paula, Atkins' Physical Chemistry, 9th ed., Oxford University Press, 2009. https://doi.org/10.1021/ed056pA260.1.

[3] I. Tunc, M. Bruns, H. Gliemann, M. Grunze, P. Koelsch, Bandgap determination and charge separation in Ag@TiO2 core shell nanoparticle films, Surf. Interface Anal. 42 (2010) 835–841. https://doi.org/10.1002/sia.3558.

[4] J. Tauc, Optical properties and electronic structure of amorphous Ge and Si, Mater. Res. Bull. 3 (1968) 37–46. https://doi.org/10.1016/0025-5408(68)90023-8.

[5] A. Jiamprasertboon, A. Kafizas, M. Sachs, M. Ling, A.M. Alotaibi, Y. Lu, T. Siritanon, I.P. Parkin, C.J. Carmalt, Heterojunction α-Fe2O3/ZnO Films with Enhanced Photocatalytic Properties Grown by Aerosol-Assisted Chemical Vapour Deposition, Chem. - A Eur. J. 25 (2019) 11337–11345. https://doi.org/10.1002/chem.201902175.

[6] P.H. Ernest, Transient spectroscopic studies of photocatalysts for CO2 and proton reduction, Imperial College London, 2015.

[7] A. Cheng, R. Tyne, Y.T. Kwok, L. Rees, L. Craig, C. Lapinee, M. D'Arcy, D.J. Weiss, P. Salaün, Investigating Arsenic Contents in Surface and Drinking Water by Voltammetry and the Method of Standard Additions, J. Chem. Educ. 93 (2016) 1945–1950. https://doi.org/10.1021/acs.jchemed.6b00025.

[8] P. Salaün, B. Planer-Friedrich, C.M.G. van den Berg, Inorganic arsenic speciation in water and seawater by anodic stripping voltammetry with a gold microelectrode, Anal. Chim. Acta. 585 (2007) 312–322. https://doi.org/10.1016/j.aca.2006.12.048.

[9] P. Salaün, K.B. Gibbon-Walsh, G.M.S. Alves, H.M.V.M. Soares, C.M.G. van den Berg, Determination of arsenic and antimony in seawater by voltammetric and chronopotentiometric stripping using a vibrated gold microwire electrode, Anal. Chim. Acta. 746 (2012) 53–62. https://doi.org/10.1016/j.aca.2012.08.013.

[10] K. Gibbon-Walsh, P. Salaün, M.K. Uroic, J. Feldmann, J.M. McArthur, C.M.G. Van Den Berg, Voltammetric determination of arsenic in high iron and manganese groundwaters, Talanta. 85 (2011) 1404–1411. https://doi.org/10.1016/j.talanta.2011.06.038.

[11] C. Vallance, C. Vallance, Determining the rate law and obtaining mechanistic information from experimental data, in: An Introd. to Chem. Kinet., Morgan and Claypool, 2017. https://doi.org/10.1088/978-1-6817-4664-7ch4.

[12] M.E. Kirby, J.C. Bullen, M.D. Hanif, H.F. Heiba, F. Liu, G.H.R. Northover, E. Resongles, D.J. Weiss, Determining the Effect of pH on Iron Oxidation Kinetics in Aquatic Environments: Exploring a Fundamental Chemical Reaction to Grasp the Significant Ecosystem Implications of Iron Bioavailability, J. Chem. Educ. (2019). https://doi.org/10.1021/acs.jchemed.8b01036.

[13] B. Ohtani, Chapter 10 - Photocatalysis by inorganic solid materials: Revisiting its definition, concepts, and experimental procedures, in: Adv. Inorg. Chem., 2011: pp. 395–430. https://doi.org/10.1016/B978-0-12-385904-4.00001-9.

[14] J.C. Bullen, J.P.L. Kenney, S. Fearn, A. Kafizas, S. Skinner, D.J. Weiss, Improved Accuracy in Multicomponent Surface Complexation Models Using Surface-Sensitive Analytical Techniques: Adsorption of Arsenic onto a TiO2/Fe2O3 Multifunctional Sorbent, J. Colloid Interface Sci. 580 (2020) 834–849. https://doi.org/10.1016/j.jcis.2020.06.119.

[15] W. Hu, J. Xie, H.W. Chau, B.C. Si, Evaluation of parameter uncertainties in nonlinear regression using Microsoft Excel Spreadsheet, Environ. Syst. Res. 4 (2015). https://doi.org/10.1186/s40068-015-0031-4.

[16] J.M. McArthur, D.M. Banerjee, K.A. Hudson-Edwards, R. Mishra, R. Purohit, P. Ravenscroft, A. Cronin, R.J. Howarth, A. Chatterjee, T. Talukder, D. Lowry, S. Houghton, D.K. Chadha, Natural organic matter in sedimentary basins and its relation to arsenic in anoxic ground water: The example of West Bengal and its worldwide implications, Appl. Geochemistry. 19 (2004) 1255–1293. https://doi.org/10.1016/j.apgeochem.2004.02.001.

[17] S.J. Hug, O.X. Leupin, M. Berg, Bangladesh and Vietnam: Different groundwater compositions require different approaches to arsenic mitigation, Environ. Sci. Technol. 42 (2008) 6318–6323. https://doi.org/10.1021/es7028284.

[18] F. Frau, D. Addari, D. Atzei, R. Biddau, R. Cidu, A. Rossi, Influence of major anions on As(V) adsorption by synthetic 2-line Ferrihydrite. Kinetic investigation and XPS study of the competitive effect of bicarbonate, Water. Air. Soil Pollut. 205 (2010) 25–41. https://doi.org/10.1007/s11270-009-0054-4.

[19] M. Kanematsu, T.M. Young, K. Fukushi, P.G. Green, J.L. Darby, Arsenic(III, V) adsorption on a goethite-based adsorbent in the presence of major co-existing ions: Modeling competitive adsorption consistent with spectroscopic and molecular evidence, Geochim. Cosmochim. Acta. 106 (2013) 404–428. https://doi.org/10.1016/j.gca.2012.09.055.

[20] M. D'Arcy, D. Weiss, M. Bluck, R. Vilar, Adsorption kinetics, capacity and mechanism of arsenate and phosphate on a bifunctional TiO2-Fe2O3 bi-composite, J. Colloid Interface Sci. 364 (2011) 205–212. https://doi.org/10.1016/j.jcis.2011.08.023.

[21] A. Katz, A. Mcdonagh, L. Tijing, H.K. Shon, Technology Fouling and Inactivation of Titanium Dioxide- Based Photocatalytic Systems Fouling and Inactivation of Titanium, Crit. Rev. Environ. Sci. Technol. 3389 (2015). https://doi.org/10.1080/10643389.2014.1000763.

[22] S.A. Kang, W. Li, H.E. Lee, B.L. Phillips, Y.J. Lee, Phosphate uptake by TiO2: Batch studies and NMR spectroscopic evidence for multisite adsorption, J. Colloid Interface Sci. 364 (2011) 455–461. https://doi.org/10.1016/j.jcis.2011.07.088.

[23] X. Huang, G.D. Foster, R. V. Honeychuck, J.A. Schreifels, The maximum of phosphate adsorption at pH 4.0: Why it appears on aluminum oxides but not on iron oxides, Langmuir. 25 (2009) 4450–4461. https://doi.org/10.1021/la803302m.
